# Antimicrobial Activity and DFT Studies of a Novel Set of Spiropyrrolidines Tethered with Thiochroman-4-one/Chroman-4-one Scaffolds

**DOI:** 10.3390/molecules27030582

**Published:** 2022-01-18

**Authors:** Nourhène Chouchène, Amani Toumi, Sarra Boudriga, Hayet Edziri, Mansour Sobeh, Mohamed A. O. Abdelfattah, Moheddine Askri, Michael Knorr, Carsten Strohmann, Lukas Brieger, Armand Soldera

**Affiliations:** 1 Laboratory of Heterocyclic Chemistry Natural Product and Reactivity (LR11ES39), Department of Chemistry, Faculty of Science of Monastir, University of Monastir, Monastir 5019, Tunisia; nourhene.chouchene23@gmail.com (N.C.); amanitoumi45@gmail.com (A.T.); moheddine.askri@fsm.rnu.tn (M.A.); 2 Laboratoire des Maladies Transmissibles et des Substances Biologiquement Actives, Faculté de Pharmacie, Monastir 5000, Tunisia; jaziri_hayet@yahoo.fr; 3 AgroBioSciences Research, Mohammed VI Polytechnic University, Lot 660–Hay MoulayRachid, Ben Guerir 43150, Morocco; mansour.sobeh@um6p.ma; 4 College of Engineering and Technology, American University of the Middle East, Kuwait; Mohamed.AbdelMoety@aum.edu.kw; 5 Institut UTINAM-UMR CNRS 6213, Université Bourgogne Franche-Comté, 16 Route de Gray, 25030 Besançon, France; 6 Faculty of Chemistry, Inorganic Chemistry, Technical University Dortmund, Otto-Hahn-Strasse 6, 44227 Dortmund, Germany; carsten.strohmann@tu-dortmund.de (C.S.); lukas.brieger@tu-dortmund.de (L.B.); 7 Laboratory of Physical Chemistry of Matter, Department of Chemistry, Université de Sherbrooke, Sherbrooke, QC J1K 2R1, Canada

**Keywords:** [3+2] cycloaddition, thiochroman-4-one, chroman-4-one, spiropyrrolidine, crystal structure, DFT

## Abstract

A novel series of 14 spiropyrrolidines bearing thiochroman-4-one/chroman-4-one, and oxindole/acenaphthylene-1,2-dione moieties were synthesized and characterized by spectroscopic techniques, as well as by three X-ray diffraction studies, corroborating the stereochemistry. Quantum chemical calculations studies, using the DFT approach, were performed to rationalize the stereochemical outcome. These *N*-heterocycles were evaluated for their antibacterial and antifungal activities against some pathogenic organisms. Several compounds displayed moderate to excellent activity towards the screened microbe strains in the study compared to Amoxicillin (AMX), Ampicillin (AMP), and Amphotericin B. Furthermore, a structural activity relationship (SAR) was established considering the synthesized compounds. Pharmacokinetic studies reveal that these derivatives exhibit an acceptable predictive ADMET profile (Absorption, Distribution, Metabolism, Excretion and Toxicity) and good drug-likeness.

## 1. Introduction

The emergence of drug-resistant pathogens has threatened human ability to treat common infections, and, thus, antimicrobial resistance (AMR) became a health threat all over the globe. The rapid spread of *Staphylococcus aureus*, for instance, is especially alarming, since it is considered to be among the most common causes of infections in health-care facilities [1,2]. Furthermore, patients suffering with infections caused by methicillin-resistant *Staphylococcus aureus* (MRSA) are more likely to die, compared to others infected with drug-sensitive infections, by about 64% [3,4]. Consequently, antimicrobial medications including antibiotics are getting more and more ineffective and, thus, infections become difficult or nearly impossible to treat. This lowered efficacy urgently increases the demand for new therapeutic agents.

**Figure 1:**
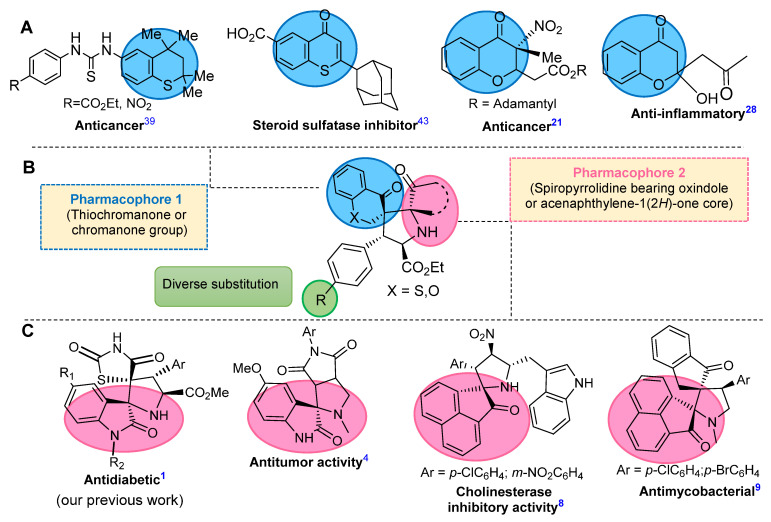
(**A**) Biologically relevant thiochroman, thiochromanone, and chromanone derivatives, (**C**) Biologically relevant spiropyrrolidines bearing oxindole or acenaphthylene-1(2*H*)-one moieties, and (**B**) Our target compounds.

In recent years, spiropyrrolidine scaffolds, especially those fused to oxindole or acenaphthylene-1(2*H*)-one moieties, and occurring in a huge number of pharmacological entities and natural compounds with significant bioactive properties, have attracted the attention of synthetic chemists [5,6,7,8,9,10,11,12,13] (Figure 1).

On the other hand, 4-chromanone derivatives represent privileged scaffolds in heterocyclic chemistry and for drug discovery. They are used as versatile intermediates for the synthesis of many natural products [14,15,16,17,18,19] and constitute interesting building blocks in drug design and organic synthesis [20,21,22,23]. They also exhibit significant biological activities allowing their use as anticancer [24,25,26,27], antifungal and antibacterial [28,29,30,31], anti-inflammatory [32,33], antidiabetic [34,35], anti-leishmanial (caused by protozoan parasites) [36,37], and insecticidal agents [38] (Figure 1).

Thiochromanones, the thio-analogs of chromanones, feature interesting scaffolds, which have been reported to exert a plethora of pronounced biological and pharmacological properties including antimicrobial [39,40,41,42], anticancer [43,44,45,46], human steroid sulfatase inhibition [47,48], *α*-adrenergic antagonist [49], antiviral [50], and insecticidal activities [51] (Figure 1).

**Scheme 1:**
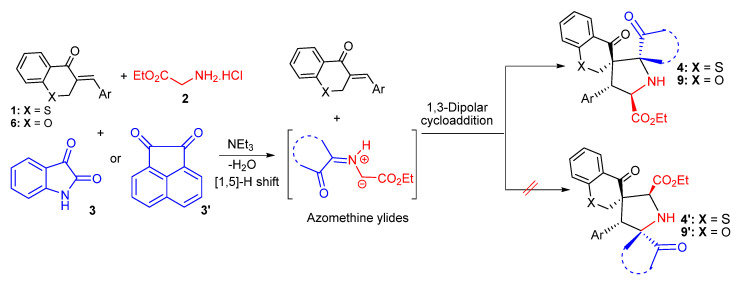
Synthesis scheme for the target compounds **4** and **9**.

In light of the significance of spiropyrrolidine and thiochromanone/chromanone heterocycles in medicinal chemistry, we felt a great potential to combine these two moieties into one single scaffold to design a novel class of hybrid spiroheterocycles, which may exhibit interesting biological profiles. The latter were obtained via multicomponent reactions (MCRs) of arylidene thiochroman-4-ones/chroman-4-ones, glycine ethyl ester, and cyclic 1,2-diketones (isatin derivatives or acenaphthenequinone) (Scheme 1). We are aware that related spiropyrrolidine(s) fused with thiochroman-4-one/chroman-4-one moieties have been prepared by Subramaniyan [52] and Rani [53], however their pharmacological profile has, up to now, never been explored. 

In continuation of our research interests in the synthesis of novel bioactive *N*-spiroheterocycles [5,54,55,56,57], we herein report the synthesis of diverse thiochromanone/chromanone substituted spiropyrrolidines (Figure 1). The latter were obtained via multicomponent reactions (MCRs) of arylidene thiochroman-4-ones/chroman-4-ones, glycine ethyl ester, and cyclic 1,2-diketones (isatin derivatives or acenaphthenequinone) (Scheme 1). These new scaffolds were tested in vitro for their antimicrobial potential. DFT calculations were carried out to obtain the optimized molecular structures of the resulting spiranic thiochromanone/chromanone-linked spiropyrrolidines. Furthermore, frontiers molecular orbitals (FMO) and molecular electrostatic potential (MEP) calculations were performed for the structural exploration of these compounds. A drug-likeness analysis was also performed.

## 2. Results and Discussion

### 2.1. Synthetic Chemistry

The synthetic strategy for our target compounds **4** and **9** is shown in Scheme 1. It is based on a multicomponent process involving the 1,3-dipolar cycloaddition reaction between exocyclic arylated alkenes derived from thiochromanone **1** or chromanone **6** and azomethine ylides. The latter were generated in situ from condensation of glycine ethyl ester **2** and a 1,2-diketone, namely isatin **3** or acenaphthenequinone **3′** (Scheme 1). 

At the onset of our investigation, we attempted to access the hybrid thiochromanone-grafted spiropyrrolidines according to our recently established reaction conditions for MCR of arylidene rhodanines, glycine ethyl ester, and cyclic 1,2-diketones [5]. For that purpose, an equivalent amount of 3-benzylidenethiochroman-4-one **1a**, glycine ethyl ester **2**, and isatin **3** was refluxed in acetonitrile in the presence of triethylamine for 2 h. The reaction smoothly afforded the desired spiroheterocycle **4a** in 80% yield (Table 1). Subsequently, a variety of dipolarophiles **1** were subjected to these reaction conditions. As shown in Table 1, this process is applicable to a wider series of arylidene thiochromanones **1** bearing electronically different substituents, or groups, on the phenyl ring, affording in good yields the targeted spiropyrrolidine products **4b**–**d** in a high regio- and diastereoselective manner as racemic mixtures. To expand the scope of this protocol, we further examined the multicomponent reaction with acenaphthenequinone **3′** as an alternative 1,2-diketone, instead of isatin **3** (Table 1), to synthetize an original spiropyrrolidines scaffold bearing an acenaphthylen-1(2*H*)-one core. The azomethine ylide derived from acenaphthenequinone seemed to be well appropriate for this process, affording the corresponding exo-spirropyrrolidine-chromanones **4e** and **4f** with good yields. Since this straightforward multicomponent [3+2] cycloaddition worked efficiently, we then studied the scope of the reaction using an oxygen surrogate, namely 5-arylidene chroman-4-ones **6**, under the same reaction conditions (Table 2).

The MCR proceeded readily providing the spiropyrrolidines exo-**9a**–**h** as the only detectable diastereoisomers (TLC monitoring) with good to excellent yield (70–94%), regardless of the electronic nature of the *p*-substituent at the aryl group (H, OCH_3_, CH_3_, and Cl) of the dipolarophile **6**. On the other hand, the steric property of the acenaphthenequinone **3′** had almost a negligible effect on the efficiency of the 1,3-dipolar cycloaddition reaction, which proceeded smoothly producing the acenaphthylen-1(2*H*)-one spiropyrrolidine-chromanones **9e**–**h** in excellent yields. All compounds are stable and were obtained as colorless or yellow solids.

#### 2.1.1. Spectroscopic and Crystallographic Characterization of Cycloadducts **4** and **9**

**Figure 2:**
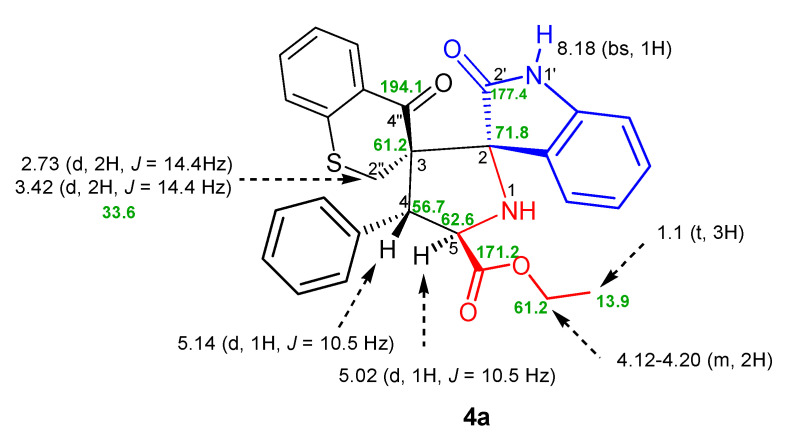
^1^H (black) and ^13^C {^1^H} NMR (green) chemical shifts (ppm) of the selected compound **4a** (see also Figure S3 in the Supporting Material).

**Figure 3:**
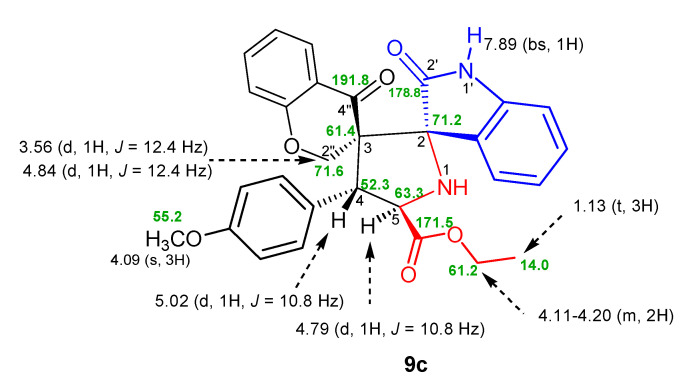
^1^H (black) and ^13^C {^1^H} NMR (green) chemical shifts (ppm) of the selected compound **9c** (see also Figure S20 in the Supporting Material).

The composition and stereochemistry of the chromanone/thiochromanone-grafted spiropyrrolidines was unambiguously elucidated by their spectroscopic data and elemental analyses. The assignments of the chemical shifts from the ^1^H and ^13^C NMR spectra of the studied compounds were done based on the literature data [5,53,55] and calculations at the B3LYP/6-311++G(2d,p) level of compounds **4a** and **9c** (Supplementary Materials; Table S1). As representative examples, relevant ^1^H and ^13^C chemical shifts of spiro-compounds **4a** and **9c** are illustrated in Figure 2 and Figure 3, respectively. 

In the ^1^H NMR spectra of **4a** and **9c**, the pyrrolidinyl protons H-4 and H-5 appear as two mutually coupled doublets at δ 5.14 and 5.02 ppm, and δ 5.02 and 4.79 ppm, respectively. This multiplicity unambiguously confirms the regiochemistry proposed in Figure 2 and Figure 3. If the hypothetical alternative regioisomers **4′**,**9′** (Scheme 1) would have been formed, the pyrrolidinyl protons should give rise to two singlets in the ^1^H NMR spectrum. Furthermore, the value of the ^3^*J* coupling constants of 10.5 and 10.8 Hz indicate that H-4 and H-5 protons are in *trans* relationship, in accordance with our earlier reports on related compounds [5,55]. A broadened singlet at δ 8.18 and 7.89 ppm can be assigned to the oxindole N-H proton of **4a** and **9c**, and a multiplet in the region between 6.59–8.17 ppm and 6.06–7.94 ppm indicates the presence of aromatic protons. Additionally, the ^1^H NMR spectra show two mutually coupled doublets at δ 2.73 and 3.42 ppm for **4a**, and 3.56 and 4.84 ppm for **9c**, corresponding to the diastereotopic 2′–CH_2_ group. The ^3^*J* values of 12.3 Hz for **4a** and 14.1 Hz for **9c** are in the same range with those previously reported values of similar spiropyrrolidine(s) fused with thiochroman-4-one/chroman-4-one moieties [52,53].

The proton decoupled ^13^C NMR spectrum of **9c** reveals the presence of two signals at δ 191.8 and δ 178.8 ppm, attributed to the carbonyl groups of chromanone and oxindole moieties, respectively. The resonances corresponding to the two spirocarbons C-2 and C-3 are observed at δ 71.2 and δ 61.4 ppm, respectively. For the assignment of the methoxy, methylene, methine, spiro, and quaternatry carbons, the DEPT-135 spectra shown in Figures S3 and S20 were recorded.

**Figure 4:**
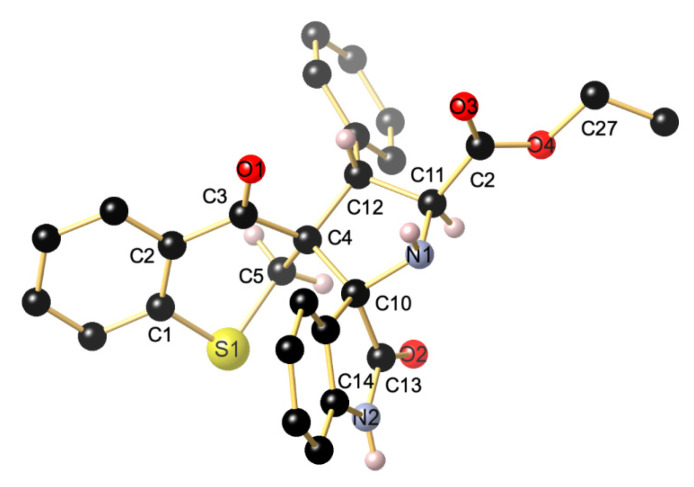
Ball and sticks presentation of the molecular structure of **4a** recorded at 100 K. Only one of the two independent molecules is shown. Apart from stereochemically relevant H atoms, all other hydrogen atoms are omitted for clarity. Furthermore, an EtOH molecule of crystallization, present in the asymmetric unit, is not depicted.

**Figure 5:**
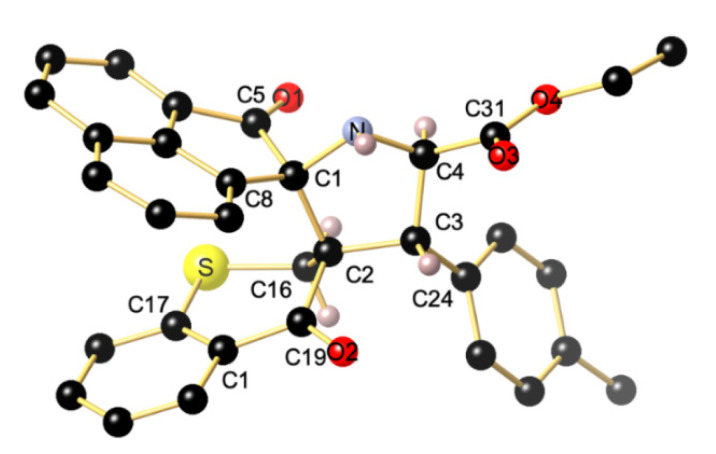
Ball and sticks presentation of the molecular structure of **4e** recorded at 100 K. Only stereochemically relevant H atoms are shown.

**Figure 6:**
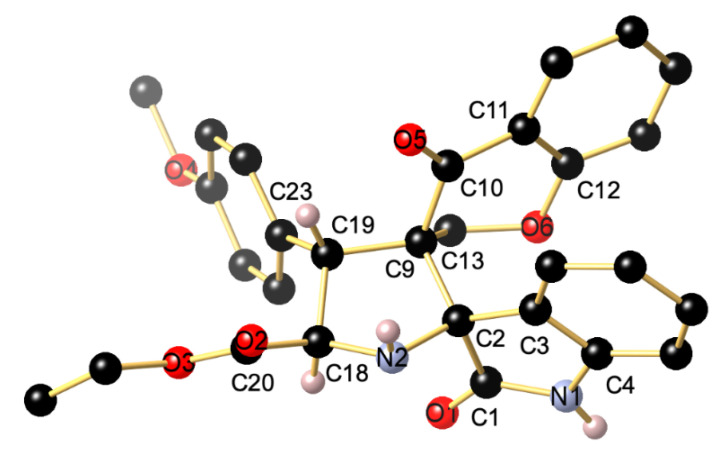
Ball and sticks presentation of the molecular structure of **9c** recorded at 100 K. Only one of the two independent molecules is shown. Apart from stereochemically relevant H atoms, all other hydrogen atoms are omitted for clarity.

The relative configuration of the stereogenic carbons in spiropyrrolidines **4** and **9** was corroborated after determination of the single-crystal X-ray structures of cycloadducts **4a**, **4e**, and **9c**, whose molecular structures are shown in Figure 4, Figure 5 and Figure 6, respectively. The structural analysis reveals that the carbonyl carbon of acenaphthenequinone moiety and (i) the carbonyl carbon group of thiochromanone/chromanone part are in *trans*-relationship, (ii) the pyrrolidinyl proton attached at C-5 (C-11, C-4, and C-18 in the crystal structures of **4a**, **4e**, and **9c**, respectively) are in *cis*-relationship (note that the crystallographic atom numbering scheme differs from that used in Figure 2 and Figure 3). 

It is noteworthy that the packing of **4a** contains two independent molecules with slightly different metric parameters, and the individual molecules are associated through intermolecular N_2_-H_2_····O_6_ and N_4_-H_4_…O_2_ hydrogen bonding with H····O bond lengths of 1.94 and 2.00 Å, respectively (see Figure S31). A similar supramolecular pairwise N-H····O association occurs also in the crystal structure of **9c** (see Figure S32).

Accordingly, we propose that the cycloadducts **4** and **9** are formed through an exo-approach between the arylidene thiochromanone/chromanone and the *Z*,*E*-dipole, as outlined above in Table 1 and Table 2. 

#### 2.1.2. DFT Mechanistic Studies

**Scheme 2:**
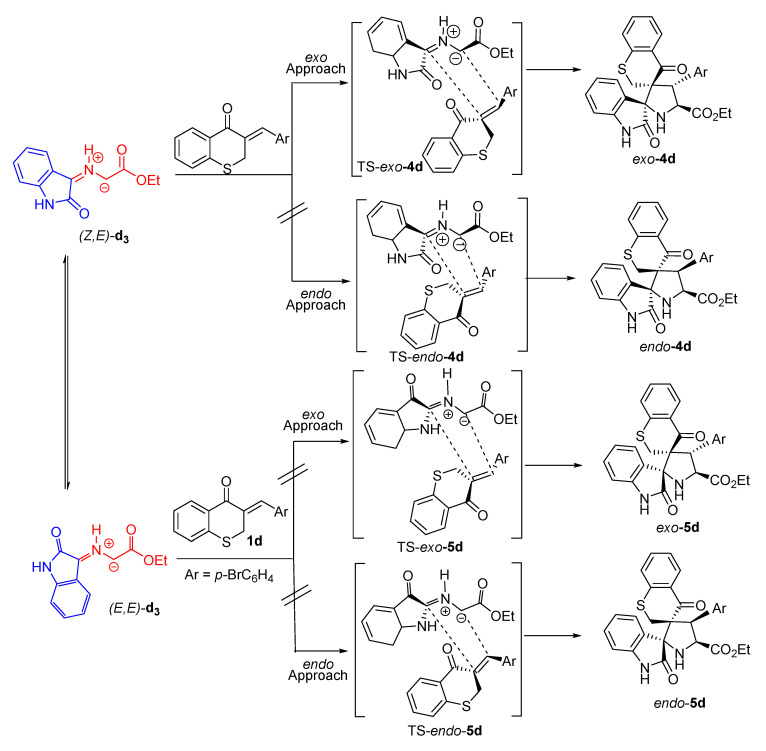
Proposed mechanism for the regio- and stereoisomeric 1,3-dipolar cycloaddition reaction of dipolarophile **1d** with azomethine ylide **d_3._**

To better grasp the experimentally observed high regio- and diastereoselectivity in the MCR, DFT calculations were performed using the 6–31G(d,p) basis set and the ωB97xd functional [58]. The effect of solvent (acetonitrile) was taken into consideration using the polarizable continuum model (PCM) approach [59,60]. We have chosen the reaction of dipolarophile **1a**, glycine ethyl ester **2**, and isatin **3** as model case study, as illustrated in Scheme 2.

In line with our previous study [55], condensation of a glycine ethyl ester and isatin, or acenaphthenequinone, followed by a [1,2]-prototropic sequence furnishes (*E*,*E*) and (*Z*,*E*) azomethine ylides are the two most stable isomers. Note that the subsequent 1,3-dipolar cycloaddition of these intermediates can a priori occur through both endo-TS and exo-TSs (Scheme 2). Kinetic and thermodynamic calculations of the Gibbs free energy (ΔG), enthalpy (ΔH), and entropy (ΔS), corresponding to all possible isomeric pathways of the reaction and relative energies for the TSs, are summarized in Table 3.

The analysis of the four located transition states reveals that TS-exo-**4d**, corresponding to the exo-approach between the (*E*)-*p*-bromobenzylidenethiochromanone **1d** and the (*Z*,*E*)-dipole, presents the lowest barriers with a ΔG value of 0.4 kcal mol^−1^, favoring the formation of the observed exo-**4d** regioisomer. Moreover, ΔG values indicate that the product exo-**4d**, identified as the sole product is more stable (ΔG, −21.8 kcal mol^−1^) than the other theoretical stereoisomers endo-**4d**, exo-**5d**, and endo-**5d**. Thus, the spiroadduct **4d** is kinetically and thermodynamically preferred, which agrees well with our experimental observations. 

### 2.2. Biological Evaluation of the Synthesized Compounds

#### 2.2.1. Antimicrobial Activity

The dispiropyrrolidine derivatives **4** and **9** were evaluated for their antibacterial activity against an assortment of five Gram-positive bacteria (*Bacillus subtilis* ATCC 6633, *Staphylococcus epidermidis CI1232*, *Staphylococcus aureus* ATCC 29213, *Staphylococcus aureus* ATCC 25923, and *Enterococcus faecalis* ATCC 29212) and four Gram-negative bacteria (*Escherichia coli* ATCC 25922, *Klebsiella pneumoniae* ATCC 4352, *Salmonella enterica 800390*, and *Pseudomonas aeruginosa* ATCC 9023). The antifungal activity of the synthesized compounds was also investigated against *Candida albicans* ATCC 90028, *Candida glabrata* ATCC 90030, and *Candida krusei* ATCC 6258. As references, to evaluate and to compare the potency of the tested compounds under the same conditions, the antibiotics Amoxicillin (AMX) and Ampicillin (AMP) were chosen as antibacterial agents. Macrocyclic Amphotericin B was used as antifungal reference. 

The in vitro antimicrobial activity of the novel dispiropyrrolidines has been assessed by the determination of the minimal inhibitory concentration (MIC) using the microdilution method, as described by Rattan [61]. The results are summarized in Table 4 and Table 5.

Most of the screened compounds show variable antibacterial activities ranging from poor to excellent against Gram-positive and Gram-negative bacteria with MIC values in the range of 32–250 µg/mL. As shown in Table 4, compounds **4a**–**e** exhibit, in most cases, interesting antibacterial potential. Compounds **4a**–**d** displayed the highest activity (MIC = 32 µg/mL) towards *B. subtilus* and *S. epidermis* compared to the reference antibiotics Amoxicillin (MIC = 64 µg/mL) and Ampicillin (MIC = 78 µg/mL).

Moreover, all these compounds were found to be more active than the standard drugs Amoxicillin (MIC = 64 µg/mL) and Ampicillin (MIC = 78 µg/mL) against the screened Gram-negative bacteria with MIC values ranging from 64 to 125 µg/mL. For the series **9a**–**h**, results presented in Table 4 indicate that the most performant derivatives exhibit an inferior activity compared to the spirocompounds of series **4**. They show only a poor to moderate activity with MIC values ranging from 64 to 250 µg/mL. It is noteworthy that compounds **4a**, **4b**, **4d**, and **4e**, with MIC value of 64 µg/mL, perform with an excellent activity, up to four times higher towards *P. aeruginosa* than Amoxicillin (MIC = 256 µg/mL). 

The compounds **9d** and **9f**–**h** (MIC = 64 µg/mL) are more potent than Amoxicillin (MIC = 78 µg/mL) against *S. epidermis* and exhibit an activity against *B. subtilus* similar to that of the reference antibiotic Ampicillin (MIC = 64 µg/mL). The results of antifungal screening (Table 5) reveal that compound **9e** was the only one exhibiting an activity comparable to that of reference antifungal Amphotericin B (MIC = 500 µg/mL) against the tested fungi.

The other synthesized compounds of series **4** were found to be more active than the standard drug with MICs ranging between 32–500 µg/mL. Notably, compound **4d** was found to be the most potent antifungal agent (MIC = 32 µg/mL) towards *C. krusei* and *C. glabrata* compared to the reference antifungal Amphotericin B (MIC =500 µg/mL).

In contrast, compounds **9a**–**h** were revealed to be less active than their analogues, **4**, against the three screened yeasts with MIC values ranging from of 125–500 µg/mL.

#### 2.2.2. Structure-Activity Relationship (SAR)

Another aim of these studies was to analyze qualitatively and quantitatively the structure-activity relationships (SAR) of our dispiropyrrolidines. As described in the literature, spiropyrrolidine derivatives are known to be excellent antimicrobial agents [8,62,63]. For the first screening of the in vivo antimicrobial results, it is clearly outlined that the presence of a thiochromanone moiety in spiropyrrolidines **4a**–**e**, independently of the nature of the *para*-substituent attached on the phenyl ring, greatly influences the inhibitory activity against all of the tested bacterial and fungal strains in comparison to their analogues containing a chromanone moiety **9a**–**h**. These results conclude the importance of the introduction of the thiochromanone moiety to improve the activity of spiropyrrolidines. Compounds **4a**–**e** especially feature a broad-spectrum bacterial and antifungal action and exerted the more potent antimicrobial effect as compared to the rest of the synthesized compounds. 

Concerning the series **9a**–**d**, bearing an oxindole core, it is interesting to note that the introduction of an electron-withdrawing bromine substituent (**9d**) at the para position of the aromatic cycle of the chromanone, caused an increase of the antibacterial activity towards *B. subtilus*, *S. epidermis*, *E.coli*, *K. pneumoniae*, and *P. aeruginosa*. In contrast, the introduction of an electron-donating group, such as a methyl (**9b**) or methoxy group (**9c**), decreased the activity potency or retained it against the same bacteria.

Among the spiropyrrolidines incorporating an acenaphthylene-1(2*H*)-one core **9e**–**h**, compound **9e**, with an unsubstituted phenyl ring, is the most active one against *K. pneumoniae* and *P. aeruginosa*. Compounds **9f** (4-MePh), **9g** (4-MeOPh), and **9h** (4-BrPh) are found to exert a superior antibacterial effect against *B. subtilus* and *S. epidermis*. The inductive or mesomeric electronic effects exerted by these groups allow, probably, each bacterium to interact in its own specific way with the corresponding compounds. 

According to the antifungal activity results and based on the study of the structure-activity relationships (SAR), we noticed that compounds containing a thiochromanone ring, **4a**–**e**, display significant antifungal activity against all the tested fungal strains, superior to those of the chromanone-grafted spiropyrrolidines, **9a**–**h**, and the reference antifungal Amphotericin B. In addition, the compound **4d** is the most potent one against *C. krusei* and *C. glabrata*. This finding shows that the presence of a bromine atom in para position of the phenyl group of chromanone seems to be responsible for the enhanced activity.

### 2.3. DFT Computational Studies

#### 2.3.1. Optimized Molecular Structures and HOMO-LUMO Energies

The ground state geometric optimization of spiropyrrolidines **4a**–**f** and **9a**–**h** was carried out in the gas phase with the *ω*B97xd functional [58] and the standard 6–31g(d,p) basis set in Gaussian 16 package [64]. The simulated electron densities distribution of the FMOs, including both HOMO and LUMO orbitals of all compounds, are indicated in Table S2 (see Supporting Information). Frontier molecular orbital energies and chemical reactivity descriptor values of the optimized geometries are summarized in Table 6 and Table 7.

The molecular frontier orbitals provide important clues to the chemical reactivity of molecules. The FMOs energy gap describes the charge transfer character from HOMO to LUMO within the studied compounds. The HOMO energy represents the tendency of a molecule to donate electrons, while the LUMO energy indicates the ability to accept electrons [65]. 

Thus, increasing the HOMO energy and decreasing the LUMO energy of the ligand lead to large intermolecular interactions with the LUMO and the HOMO of the receptor. The extent of these stabilizing interactions between ligand-receptor interacting orbitals correlates with the HOMO-LUMO energy gap. The softest molecules are determined with small energy gap values (ΔE_gap_ = E_LUMO_ − E_HOMO_) and are also identified as more stable and, hence, more reactive, according to Pearson’s “Hard and Soft Acids and Bases” principle [66]. 

As shown from Table 4, the HOMO and LUMO electron-density of spiropyrrolidines **4a**–**f** is delocalized on the thiochromanone ring except in **4e** and **4f**, where the electron-density of the LUMO, which indicates the electrophilic attack site, is mainly distributed over the acenaphtene nucleus. For the designed compounds **9a**–**f**, the HOMO orbital was found to be distributed all over the pyrrolidine ring and isatin or acenaphtenequinone nucleus. However, the LUMO orbitals are dispersed over the chromanone ring in the series of compounds **9a**–**d** and on the acenaphtenequinone ring in compounds **9e**–**h**. Interestingly, the spiropyrrolidines **4a**–**f** bearing a thiochroman-4-one moiety show smaller energy gap values, ranging between 1.636 and 1.965 eV, compared to spiropyrrolidine derivatives **9a**–**h** featuring a chroman-4-one scaffold (ΔE_gap_ in the range of 2.552 and 2.890 eV). Consequently, compounds **4a**–**f** are the most reactive, compared to compounds **9a**–**h**, which show the lowest reactivity (the most chemically stable). These results may explain the higher biological activity of compounds **4a**–**f**, providing a good match with the experimental antifungal and antibacterial data. 

The reactivity descriptors, based on the analysis of the electronic chemical potential (μ), the chemical hardness (η), and the electrophilicity index (ω), provide useful insight into the chemical reactivity and stability of the molecules. The chemical potential describes the electron transfer capacity that occurs in the molecule in the fundamental state and the propensity of electrons to escape from an equilibrium system, whereas the chemical hardness indicates the resistance to charge transfer [67,68]. As shown in Table 4, compounds **4a**–**f** showed higher chemical potential (ranging from −6.477 to −6.679 eV) and lower hardness values (in the range of 0.818 to 0.983 eV). Consequently, the spiropyrrolidines **4a**–**f** are softer and more reactive than the chromanone-grafted spiropyrrolidines **9a**–**h**. The biological activity of compounds **4a**–**f** can, thus, be affected by the strong charge transfer interaction. The electrophilicity (ω), which defines the tendency of molecules to attract electrons [69], is also superior in the series of spiropyrrolidines **4a**–**d**. Hence, the bioactivity of these compounds may be explained by the ability of the biological target to receive electrons from neighboring molecules, which may be important for stabilization of the active site.

#### 2.3.2. Molecular Electrostatic Potential (MEP)

The MEP has emerged as a powerful approach in drug design and molecular biology to understand the intermolecular interactions between molecules and their biological receptors (proteins, enzymes). The MEP is applied to explore the chemical affinity and most likely binding modes with a molecular receptor, thus providing insight into the structure activity relationship of bioactive compounds. MEP-mapped surfaces inform on the chemical reactivity of the molecules and provide a reliable description of the charge distributions in a pictorial form. They also indicate the most likely electrophilic and nucleophilic reactive sites, polarization of molecules, as well as hydrogen bonding interactions [70,71,72,73].

**Figure 7:**
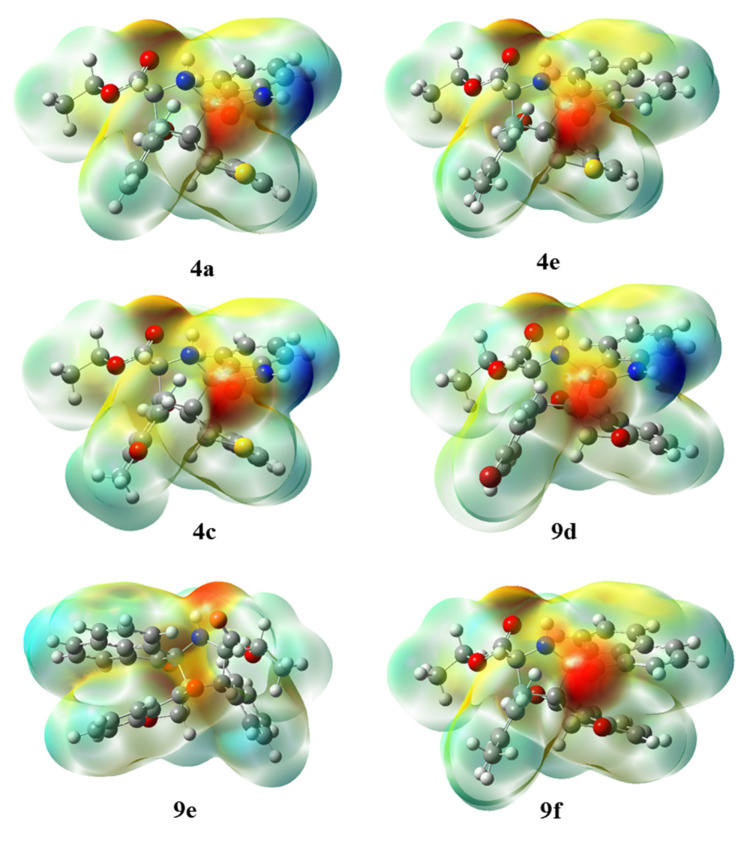
Molecular electrostatic potential (MEP) formed by mapping of the total density over the electrostatic potential for compounds **4a**, **4e**, **4c**, **9d**, **9e**, and **9f**.

The sites related to the polar and nonpolar regions of the molecule are visualized by color variations. Electrophilic regions are indicated by blue coloration (electron-deficient regions), while nucleophilic regions are shown in red color (electron-rich regions). The electrostatic potential maps of the selected compounds **4a**, **4e**, **9c**, **9d**, **9e**, and **9f** are shown in Figure 7.

### 2.4. Drug-Likeness Analysis

Analyzing the physicochemical properties of the developed drug hits is a crucial step to analyze and determine their drug-likeness potential. In this regard, quantitative structure-activity relationships (QSAR) descriptors, which can be calculated by means of a variety of software packages, may be a very useful tool. In this work, we evaluated the drug-likeness potential of the six selected compounds (**4a**, **4d**, **4e**, **9c**, **9d**, and **9e**) through computing various QSAR descriptors, according to the Lipinski rule of five [74] and the Veber’s parameters (Table 8) [75]. 

Molecular weights of the compounds range from 485.58 to 564.48 Da. Derivatives **4a** and **9c**, having molecular weights less than 500 Da, obey the first Lipinski rule for effective and safe drug delivery. Lipinski’s second rule stipulates that drug-like compounds should not possess more than five hydrogen bond donating groups. All of the six compounds comply with this rule. Hydrogen bond accepting groups in all compounds are in the range of 5–8, which is less than ten, thus also meeting Lipinski’s third rule. The determined logP value was 2.99–5.75. All compounds, therefore, satisfy Lipinski’s rule regarding logP with a value inferior to five. An exception is compound **4e**, whose logP value exceeds five. Compounds **4a** and **9c** showed zero Lipinski violations, compounds **4d**, **9d**, and **9e** violated one rule, while compound **4e** violated two rules and, thus, failed to show a drug-likeness potential (Table 8). Veber’s parameters, such as molar refractivity (mr) and total polar surface area (TPSA), demonstrate the polarizability of the compounds and suggest their oral bioavailability [75]. The mr values of the six compounds did not fall in the recommended range of 40–130 cm^3^/mol, however, this was offset by their TPSA value, which was not superior to 140 for any of the compounds. This suggests, for derivatives, an appreciable oral bioavailability. Besides, the number of rotatable bonds is four or five, which is inferior to 10. Except for compound **4e**, which failed in drug-likeness parameters, some additional parameters, such as solubility, van der Waals volume, and number of hydrophobic atoms (a-hyd) were found to be in the acceptable range of −6 to −8, 451–478, and 26–31, respectively. 

## 3. Materials and Methods

### 3.1. Apparatus and General Information

The ^1^H NMR spectra were recorded at 300 and 400 MHz using a Bruker Avance 300 or Bruker Avance III-400 machine (Rheinstetten, Germany). The chemical shifts were recorded in ppm relative to TMS and with the solvent resonance as the internal standard. Data were reported as follows: chemical shift, multiplicity (bs = broad singlet, s = singlet, d = doublet, t = triplet, m = multiplet), coupling constants (Hz), integration. ^13^C {^1^H} NMR data were collected at 75 or 100 MHz with complete proton decoupling with the solvent resonance as the internal standard. Elemental analyses were performed on a Perkin Elmer 2400 Series II Elemental CHNS analyzer (Waltham, MA, USA). Materials: thin-layer chromatography (TLC): TLC plates (Merck, silica gel 60 F254 0.2 mm, 200 × 200 nm) (Darmstadt, Germany); substances were detected using UV light at 254 nm.

### 3.2. General Procedure for Preparation of Cycloadducts **4** and **9**

A mixture of 3-arylidenethiochroman-4-ones/3-arylidenecroman-4-ones (1 mmol), ethyl glycinate hydrochloride (1 mmol) **2**, isatin **3** or acenaphthenequinone **3′** (1 mmol), and Et_3_N (1 mmol) in acetonitrile (5 mL) was heated under reflux for 2h. After completion of the reaction (TLC monitoring), the solvent was removed under vacuum. The residue was chromatographed on silica gel employing ethylacetate-cyclohexane (3:7 *v*/*v*) as eluent to obtain the pure products **4** and **9**.

#### 3.2.1. (2S*,3R*,4*S,5S*)-Spiro[2,3′]-oxindole-spiro[3,3″]thiochroman-4″-one-4-phenyl-5-carboxyethoxypyrrolidine (**4a**)

White solid; Yield: (387 mg, 80%); mp (°C ± 2) = 184 °C; IR (KBr) υ: 3267, 2918, 1723, 1692, 1650, 1183, 749 cm^−1^; ^1^H NMR (300 MHz, CDCl_3_) δ_H_: 1.10 (t, *J* = 7.2 Hz, 3H, CH_3_(ester)), 2.73 (d, *J* =14,4 Hz, 1H, H-2″), 3.42 (d, *J* = 14.4 Hz, 1H, H-2″), 4.12–4.2 (m, 2H, CH_2_(ester)), 5.02 (d, *J* = 10.5 Hz, 1H, H-4), 5.14 (d, *J* = 10.5 Hz, 1H, H-5), 6.59–6.62 (m, 1H, Ar-H), 6.72–6.74 (m, 1H, Ar-H), 6.81–6.84 (m, 1H, Ar-H), 6.97–7.05 (m, 2H, Ar-H), 7.09–7.12 (m, 2H, Ar-H), 7.28–7.40 (m, 3H, Ar-H), 7.56–7.58 (m, 1H, Ar-H), 8.14–8.17 (m, 1H, Ar-H), 8.18 (bs, 1H, H-1′); ^13^C NMR (75 MHz, CDCl_3_) δ_C_: 13.9, 33.6, 56.7, 61.2, 61.7, 62.6, 71.8, 110.0, 122.0, 124.7, 125.6,126.0, 126.6, 127.6,128.5, 126.6, 130.1, 130.7, 132.6, 136.2, 141.4, 142.2, 171.2, 177.4, 194.1. Anal. Calcd for C_28_H_24_N_2_O_4_S: C, 69.40; H, 4.99; N, 5.78%. Found: C, 69.07; H, 4.95; N, 5.79%.

#### 3.2.2. (2S*,3R*,4*S,5S*)-Spiro[2,3′]-oxindole-spiro[3,3″]thiochroman-4″-one-4-(*p*-methyl phenyl)-5-carboxyethoxypyrrolidine (**4b**)

White solid; Yield: (423 mg, 85%); mp (°C ± 2) = 218 °C; IR (KBr) υ: 3323, 2969, 1714, 1700, 1658, 1195, 759 cm^−1^; ^1^H NMR (300 MHz, CDCl_3_) δ_H_: 1.13 (t, *J* = 6.9 Hz, 3H, CH_3_(ester)), 2.69 (s, 3H, CH_3_), 2.74 (d, *J* = 14.1 Hz, 1H, H-2″), 3.39 (d, *J* = 14.4 Hz, 1H, H-2″), 4.11–4.19 (m, 2H, CH_2_ (ester)), 4.95 (d, *J* = 10.2 Hz, 1H, H-4), 5.05 (d, *J* = 10.5 Hz, 1H, H-5), 6.55–6.60 (m, 1H, Ar-H), 6.68–6.81 (m, 3H, Ar-H), 6.89–7.32 (m, 4H, Ar-H), 7.41–7.43 (m, 2H, Ar-H), 8.09–8.14 (m, 2H, Ar-H); ^13^C NMR (75 MHz, CDCl_3_) δ_C_: 13.5, 21.0, 32.2, 54.6, 55.4, 60.6, 61.9, 63.1, 72.0, 109.5, 113.1, 121.5, 124.6, 126.6, 129.0, 129.4, 131.3, 132.0, 132.3, 141.5, 141.6, 158.4, 178.1, 192.4. Anal. Calcd for C_29_H_26_N_2_O_4_S: C, 69.86; H, 5.26; N, 5.62%. Found: C, 69.53; H, 5.30; N, 5.63%.

#### 3.2.3. (2S*,3R*,4*S,5S*)-[2,3′]-oxindole-spiro[3,3″]thiochroman-4″-one-4-(p-methoxyphenyl)-5-carboxyethoxypyrrolidine (**4c**)

White solid; Yield: (401 mg, 78%); mp (°C ± 2) = 168 °C; IR (KBr) υ: 3270, 2925, 1734, 1697, 1655, 1194, 764 cm^−1^; ^1^H NMR (300 MHz, CDCl_3_) δ_H_: 1.12 (t, *J* = 7.2 Hz, 3H, CH_3_(ester)), 2.53 (d, *J* = 14.1 Hz, 1H, H-2″), 3.39 (d, *J* = 14.1 Hz, 1H, H-2″), 3.80 (s, 3H, OCH_3_), 4.12–4.19 (m, 2H, CH_2_(ester)), 4.93 (d, *J* = 10.5 Hz, 1H, H-4), 4,99 (d, *J* = 10.5 Hz, 1H, H-5), 6.57–6.72 (m, 2H, Ar-H), 6.80–6.90 (m, 4H, Ar-H), 6.96–7.10 (m, 3H, Ar-H), 7.46–7.48 (m, 2H, Ar-H), 8.13–8.15 (m, 1H, Ar-H), 8.22 (bs, 1H, H-1′); ^13^C NMR (75 MHz, CDCl_3_) δ_C_: 13.5, 33.2, 54.6, 56.0, 60.5, 61.4, 62.6, 71.5, 109.2, 113.4, 121.4, 124.1, 125.4, 125.9, 126.0, 128.0, 128.8, 130.1, 130.3, 130.6, 131.9, 140.8, 141.7, 158.4, 171.3, 177.8, 193.8. Anal. Calcd for C_29_H_26_N_2_O_4_S: C, 67.69; H, 5.09; N, 5.44%. Found: C, 67.36; H, 5.13; N, 5.45%.

#### 3.2.4. (2S*,3R*,4*S,5S*)-[2,3′]-oxindole-spiro[3,3″]thiochroman-4″-one-4-(p-bromophenyl)-5-carboxyethoxypyrrolidine (**4d**)

White solid; Yield: (490 mg, 87%); mp (°C ± 2) = 221 °C; IR (KBr) υ: 3277, 2922, 1744, 1708, 1660, 1178, 754 cm^−1^; ^1^H NMR (300 MHz, CDCl_3_) δ_H_: 1.12 (t, *J* = 7.2 Hz, 3H, CH_3_(ester)), 2.70 (d, *J* = 14.4 Hz, 1H, H-2″), 3.32 (d, 1H, *J* = 14.4 Hz, 1H, H-2″), 4.13–4.21 (m, 2H, CH_2_(ester)), 4.93 (d, *J* = 10.2 Hz, 1H, H-4), 5.11 (d, *J* = 10.5 Hz, 1H, H-5), 6.53–6.58 (m, 1H, Ar-H), 6.74–6.92 (m, 3H, Ar-H), 6.96–7.15(m, 3H, Ar-H), 7.49–7.59 (m, 4H, Ar-H), 8.13–8.14 (m, 1H, Ar-H), 8.16 (bs, 1H, H-1′); ^13^C NMR (75 MHz, CDCl_3_) δ_C_:14.0, 33.5, 55.5, 61.0, 61.7, 62.4, 71.7, 110.4, 121.9, 122.1, 124.3, 125.0, 126.8, 130.0, 130.5, 130.7, 131.8, 131.9, 133.0, 135.0, 141.9, 142.3, 170.4, 176.3, 194.2. Anal. Calcd for C_28_H_23_BrN_2_O_4_S: C, 59.69; H, 4.11; N, 4.97%. Found: C, 59.58; H, 4.26; N, 5.01%.

#### 3.2.5. (2S*,3R*,4*S,5S*)-Spiro[2,2′]-acenaphthene-1′-one-spiro[3,3′]thiochroman-4″-one-4-(*p*-methylphenyl)-5-carboxyethoxypyrrolidine (**4e**)

White solid; Yield: (469 mg, 88%); mp (°C ± 2) = 128 °C; IR (KBr) υ: 3291, 3054, 1724, 1709, 1661, 1188, 783, 749 cm^−1^; ^1^H NMR (300 MHz, CDCl_3_) δ_H_: 1.11 (t, *J* = 7.2 Hz, 3H, CH_3_(ester)), 2.35 (s, 3H, CH_3_), 2.70 (d, *J* = 14.1 Hz, 1H, H-2″), 3.30 (d, *J* = 14.4 Hz, 1H, H-2″), 4.13–4.19 (m, 2H, CH_2_(ester)), 5.13 (s, 2H, H-4 and H-5), 6.49–6.52 (m, 1H, Ar-H), 6.94–6.98 (m, 2H, Ar-H), 7.15–7.27 (m, 4H, Ar-H), 7.46–7.48 (m, 2H, Ar-H), 7.59–7.61 (m, 1H, Ar-H), 7.66–7.71 (m, 1H, Ar-H), 7.39–7.99 (m, 2H, Ar-H), 8.05–8.08 (m, 1H, Ar-H); ^13^C NMR (75 MHz, CDCl_3_) δ_C_: 14.0, 21.0, 33.5, 56.3, 61.3, 62.7, 63.1, 74.7, 122.2, 122.7, 124.7, 125.6, 126.6, 127.6, 128.1, 129.3, 129.7, 130.3, 130.5, 130.9, 131.3, 131.9, 132.4, 132.7, 135.6, 137.3, 141.7, 171.3, 194.0, 201.8. Anal. Calcd for C_33_H_27_NO_4_S: C, 74.28; H, 5.10; N, 2.62%. Found: C, 74.06; H, 5.14; N, 2.60%.

#### 3.2.6. (2S*,3R*,4*S,5S*)-Spiro[2,2′]-acenaphthene-1′-one-spiro[3,3″]thiochroman-4″-one-4-(p-methoxyphenyl)-5-arboxyethoxypyrrolidine (**4f**)

White solid; Yield: (494 mg, 90%); mp (°C ± 2) = 114 °C; IR (KBr) υ: 3282, 2990, 1718, 1705, 1662, 1186, 779, 736 cm^−1^; ^1^H NMR (300 MHz, CDCl_3_) δ_H_: 1.09 (t, *J* = 7.2 Hz, 3H, CH_3_(ester)), 2.68 (d, *J* = 14.4 Hz, 1H, H-2″), 3.30 (d, *J* = 14.4 Hz, 1H, H-2″), 3.79 (s, 3H, OCH_3_), 4.1–4.16 (m, 2H, CH_2_(ester)), 5.01 (d, *J* = 10.8 Hz, 1H, H-4), 5.11 (d, *J* = 10.8 Hz, 1H, H-5), 6.47–6.48 (m, 1H, Ar-H), 6.87–6.94 (m, 3H, Ar-H), 7.12–7.26 (m, 2H, Ar-H), 7.48–7.56 (m, 3H, Ar-H), 7.62–7.72 (m, 2H, Ar-H), 7.88–7.94 (m, 2H, Ar-H), 8.02–8.05 (m, 1H, Ar-H); ^13^C NMR (75 MHz, CDCl_3_) δ_C_: 14.0, 33.5, 55.0, 56.3, 61.3, 62.7, 63.1, 74.7, 122.2, 122.7, 124.7, 125.6, 126.6, 127.6, 128.1, 129.3, 129.7, 130.3, 130.5, 130.9, 131.3, 131.9, 132.4, 132.7, 135.6, 136.0, 137.3, 171.3, 194.0, 201.8. Anal. Calcd for C_33_H_27_NO_5_S: C, 72.11; H, 4.95; N, 2.55%. Found: C, 72.44; H, 4.99; N, 2.57%.

#### 3.2.7. (2S*,3R*,4*S,5S*)-Spiro[2,3′]-oxindole-spiro[3,3″]chroman-4″-one-4-phenyl-5-carboxyethoxypyrrolidine (**9a**)

White solid; Yield: (402 mg, 86%) mp (°C ± 2) =182 °C; IR (KBr) υ: 3331, 2974, 1726, 1701, 1682, 759 cm^−1^; ^1^H NMR (300 MHz, CDCl_3_) δ_H_: 1.00 (t, *J* = 7.2 Hz, 3H, CH_3_(ester)), 3.46 (d, *J* = 12.3 Hz, 1H, H-2″), 4.01–4.10 (m, 2H, CH_2_(ester)), 4.72 (d, *J* = 10.5 Hz, 1H, H-4); 4.75 (d, *J* = 12.3 Hz, 1H, H-2″), 4.98 (d, *J* = 10.5 Hz, 1H, H-5), 6.47–6.50 (m, 1H, Ar-H), 6.72–6.78 (m, 2H, Ar-H), 6.92–6.98 (m, 2H, Ar-H), 7.13–7.15 (m, 1H, Ar-H), 7.23–7.30 (m, 3H, Ar-H), 7.49–7.52 (m, 2H, Ar-H), 7.61–7.64 (m, 1H, Ar-H), 8.17 (bs, 1H, H-1′); ^13^C NMR (75 MHz, CDCl_3_) δ_C_: 13.9, 53.0, 61.1, 61.6, 63.1, 71.3, 71.5, 109.3, 116.7, 120.8, 121.1, 122.2, 125.8, 126.6, 127.7, 128.5, 129.3, 129.5, 134.9, 135.3, 140.8, 161.1, 171.6, 179.2, 191.7. Anal. Calcd for C_28_H_24_N_2_O_5_: C, 71.78; H, 5.16; N, 5.98%. Found: C, 71.60; H, 5.06; N, 5.89%.

#### 3.2.8. (2S*,3R*,4*S,5S*)-Spiro[2,3′]-oxindole-spiro[3,3″]chroman-4″-one-4-(*p*-methylphenyl)-5-carboxyethoxypyrrolidine (**9b**)

Yellow solid; Yield: (366 mg, 76%); mp (°C ± 2) = 226 °C; IR (KBr) υ: 3281, 3002, 1716, 1695, 1675, 764 cm^−1^; ^1^H NMR (400 MHz, CDCl_3_) δ_H_: 1.10 (t, *J* = 7.1 Hz, 3H, CH_3_(ester)), 2.30 (s, 3H, CH_3_), 3.54 (d, *J* = 12.4 Hz, 1H, H-2″), 4.10–4.16 (m, 2H, CH_2_(ester)), 4.78 (d, *J* = 10.8 Hz, 1H, H-4), 4.84 (d, *J* = 12.4 Hz, 1H, H-2″), 5.02 (d, *J* = 10.8 Hz, 1H, H-5), 6.59 (d, *J* = 8.1 Hz, 2H, Ar-H), 6.80–6.69 (m, 2H, Ar-H), 7.02–6.92 (m, 2H, Ar-H), 7.21–7.10 (m, 3H, Ar-H), 7.39 (d, *J* = 7.8 Hz, 2H, Ar-H), 7.62 (dd, *J* = 7.9 Hz, *J* = 1.5 Hz, 1H, Ar-H), 8.36 (bs, 1H, H-1′); ^13^C NMR (100 MHz, CDCl_3_) δ_C_: 14.0, 21.1, 52.7, 61.2, 61.5, 63.2, 71.3, 71.5, 109.3, 116.7, 121.1, 122.2, 125.8, 127.7, 129.2, 129.5, 131.7, 135.4, 137.3, 161.1, 171.7, 179.3, 191.8. Anal. Calcd for C_29_H_26_N_2_O_5_: C, 71.78; H, 5.16; N, 5.98%. Found: C, 71.60; H, 5.06; N, 5.89%.

#### 3.2.9. (2S*,3R*,4*S,5S*)-Spiro[2,3′]-oxindole-spiro[3,3″]chroman-4″-one-4-(p-methoxyphenyl)-5-carboxyethoxypyrrolidine (**9c**)

Yellow solid; Yield: (418 mg, 84%); mp (°C ± 2) = 228 °C; IR (KBr) υ: 3315, 2985, 1721, 1688, 1674, 748 cm^−1^; ^1^H NMR (300 MHz, CDCl_3_) δ_H_: 1.13 (t, *J* = 7.1 Hz, 3H, CH_3_(ester)), 3.51 (d, *J* = 12.3 Hz, 1H, H-2″), 3.81 (s, 3H, OCH_3_), 4.13–4.19 (m, 2H, CH_2_(ester)), 4.76 (d, *J* = 12.3 Hz, 1H, H-2″), 4.91 (d, *J* = 11.1 Hz, 1H, H-4), 5.09 (d, *J* = 11.1 Hz, 1H, H-5), 6.00 (d, *J* = 8.3 Hz, 1H, Ar-H), 6.53 (t, *J* = 7.5 Hz, 1H, Ar-H), 6.94–6.71 (m, 3H, Ar-H), 7.36 (ddd, *J* = 33.3 Hz, *J* = 18.9 Hz, *J* = 7.7 Hz, 4H, Ar-H), 7.52 (d, *J* = 8.1 Hz, 1H, Ar-H), 7.60 (t, *J* = 7.6 Hz, 1H, Ar-H), 7.83 (d, *J* = 8.1 Hz, 1H, Ar-H), 7.91 (d, *J* = 7.0 Hz, 1H, Ar-H); ^13^C NMR (75 MHz, CDCl_3_) δ_C_: 14.0, 52.3, 55.2, 61.2, 61.4, 63.3, 71.2, 71.6, 109.2; 113.9, 116.7, 120.8, 121.1, 122.2, 125.8, 126.4, 126.7, 127.7, 129.5, 135.3, 140.6, 159.0, 161.1, 171.5, 178.8, 191.8. Anal. Calcd for C_29_H_26_N_2_O_6_: C, 69.87; H, 5.26; N, 5.62%. Found: C, 69.00; H, 5.37; N, 5.59%.

#### 3.2.10. (2S*,3R*,4*S,5S*)-Spiro[2,3′]-oxindole-spiro[3,3″]chroman-4″-one-4-(*p*-bromophenyl)-5-carboxyethoxypyrrolidine (**9d**)

White solid; Yield: (514 mg, 94%); mp (°C ± 2) = 234 °C; IR (KBr) υ: 3323, 2969, 1714,1694, 16969, 749 cm^−1^; ^1^H NMR (400 MHz, CDCl_3_) δ_H_: 1.11 (t, *J* = 7.1 Hz, 3H, CH_3_(ester)), 3.53 (d, *J* = 12.3 Hz, 1H, H-2″), 4.10–4.18 (m, 2H, CH_2_(ester)), 4.77 (t, *J* = 10.7 Hz, 2H, H-4 and H-2″), 5.00 (d, *J* = 10.6 Hz, 1H, H-5), 6.62–6.50 (m, 2H, Ar-H), 6.72 (dt, *J* = 28.4 Hz, *J* =7.6 Hz, 2H, Ar-H), 6.98–6.86 (m, 2H, Ar-H), 7.21–7.10 (m, 1H, Ar-H), 7.42 (m, 4H, Ar-H), 7.62 (dd, *J* = 7.9 Hz, *J* =1.4 Hz, 1H, Ar-H); 8.22 (bs, 1H, H-1′); ^13^C NMR (100 MHz, CDCl_3_) δ_C_: 14.0, 52.3, 61.3, 63.2, 71.4, 71.5, 109.4, 116.7, 120.7, 121.3, 121.7, 125.8, 127.8, 129.6, 131.1, 131.7, 134.2, 135.6, 140.7, 161.1, 171.4, 191.7. Anal. Calcd for C_28_H_23_BrN_2_O_5_: C, 61.44; H, 4.24; N, 5.12%. Found: C, 61.58; H, 4.16; N, 5.20%.

#### 3.2.11. (2S*,3R*,4*S,5S*)-Spiro[2,2′]-acenaphthene-1′-one-spiro[3,3″]chroman-4″-one-4-phenyl-5-carboxyethoxypyrrolidine (**9e**)

Yellow solid; (352 mg, 70%); mp (°C ± 2) = 188 °C; IR (KBr) υ: 3279, 2911, 1726, 1693, 1674, 1193, 774, 760 cm^−1^; ^1^H NMR (400 MHz, CDCl_3_) δ_H_: 1.10 (t, *J* = 7.1 Hz, 3H, CH_3_(ester)), 3.47 (d, *J* = 12.3 Hz, 1H, H-2″), 4.13–4.18 (m, 2H, CH_2_(ester)), 4.75 (d, *J* = 12.3 Hz, 1H, H-2″), 4.90 (d, *J* = 11.2 Hz, 1H, H-4), 5.10 (d, *J* = 11.1 Hz, 1H, H-5), 6.01 (d, *J* = 7.9 Hz, 1H, Ar-H), 6.64–6.45 (m, 1H, Ar-H), 6.83 (m, 1H, Ar-H), 7.15 (d, *J* = 7.8 Hz, 2H, Ar-H), 7.47–7.20 (m, 5H, Ar-H), 7.55 (m, 2H, Ar-H), 7.85 (d, *J* = 8.0 Hz, 1H, Ar-H), 7.93 (d, *J* = 6.9 Hz, 1H, Ar-H); ^13^C NMR (100 MHz, CDCl_3_) δ_C_: 14.0, 52.2, 61.2, 62.9, 63.2, 71.0, 74.6, 116.1, 120.7, 120.8, 121.5, 122.2, 125.1, 127.2, 127.9, 128.9, 129.2, 129.8, 131.0, 131.5, 131.7, 134.7, 136.9, 137.3, 140.8, 160.0, 171.9, 191.6. Anal. Calcd for C_32_H_25_NO_5_: C, 76.33; H, 5.00; N, 2.78%. Found: C, 76.99; H, 5.06; N, 2.70%.

#### 3.2.12. (2S*,3R*,4*S,5S*)-Spiro[2,2′]-acenaphthene-1′-one-spiro[3,3″]chroman-4″-one-4-(*p*-methylphenyl)-5-carboxyethoxypyrrolidine (**9f**)

Yellow solid; Yield: (377 mg, 73%); mp (°C ± 2) = 194 °C; IR (KBr) υ: 3288, 2898, 1739, 1700, 1667, 1201, 786, 767 cm^−1^; ^1^H NMR (400 MHz, CDCl_3_) δ_H_: 1.10 (t, *J* = 7.1 Hz, 3H, CH_3_(ester)), 2.30 (s, 3H, CH_3_), 3.47 (d, *J* = 12.3 Hz, 1H, H-2″), 4.10–4.17 (m, 2H, CH_2_(ester)), 4.75 (d, *J* = 12.3 Hz, 1H, H-2″), 4.90 (d, *J* = 11.2 Hz, 1H, H-4), 5.10 (d, *J* = 11.1 Hz, 1H, H-5); 6.01 (d, *J* = 7.9 Hz, 1H, Ar-H), 6.64–6.45 (m, 1H, Ar-H), 6.83 (m, 1H, Ar-H), 7.15 (d, *J* = 7.8 Hz, 2H, Ar-H), 7.47–7.20 (m, 5H, Ar-H), 7.55 (m, 2H, Ar-H), 7.85 (d, *J* = 8.0 Hz, 1H, Ar-H), 7.93 (d, *J* = 6.9 Hz, 1H, Ar-H); ^13^C NMR (100 MHz, CDCl_3_) δ_C_: 14.0, 21.0, 52.7, 61.2, 63.2, 71.3, 71.5, 109.3, 116.7, 120.1, 122.2, 125.8, 126.6, 127.7, 129.2, 129.5, 131.7, 135.7, 140.8, 161.1, 171.7, 179.3, 191.8. Anal. Calcd for C_33_H_27_NO_5_: C, 76.58; H, 5.26; N, 2.71%. Found: C, 76.49; H, 5.38; N, 2.76%.

#### 3.2.13. (2S*,3R*,4*S,5S*)-Spiro[2,2′]-acenaphthene-1′-one-spiro[3,3″]chroman-4″-one-4-(*p*-methoxyphenyl)-5-carboxyethoxypyrrolidine (**9g**)

Yellow solid; Yield: (464 mg, 87%); mp (°C ± 2) =184 °C; IR (KBr) υ: 3292, 2899, 1737, 1704, 1677, 1199, 773, 747 cm^−1^; ^1^H NMR (400 MHz, CDCl_3_) δ_H_: 1.10 (t, *J* = 7.1 Hz, 3H, CH_3_(ester)), 3.55 (d, *J* = 12.4 Hz, 1H, H-2″), 3.77 (s, 3H, OCH_3_), 4.10–4.16 (m, 2H, CH_2_(ester)), 4.76 (d, *J* = 10.8 Hz, 1H, H-4), 4.83 (d, *J* = 12.3 Hz, 1H, H-2″), 4.98 (d, *J* =10.8 Hz, 1H, H-5), 6.59 (d, *J* = 8.0 Hz, 2H, Ar-H), 6.74 (dt, *J* = 15.3 Hz, *J* = 7.6 Hz, 2H, Ar-H), 6.87 (d, *J* = 8.5 Hz, 2H, Ar-H), 6.94 (m, 2H, Ar-H), 7.17 (t, *J* = 7.3 Hz, 1H, Ar-H), 7.43 (d, *J* = 8.3 Hz, 2H, Ar-H), 7.62 (m, 1H, Ar-H); ^13^C NMR (100 MHz, CDCl_3_) δ_C_: 14.0, 52.4, 55.2, 61.2, 61.5, 63.4, 71.4, 71.6, 109.3, 113.9, 116.7, 120.8, 121.1, 122.2, 125.8, 126.6, 126.8, 127.7, 129.4, 130.4, 135.4, 140.8, 159.0, 161.1, 171.1, 171.8, 191.9. Anal. Calcd for C_33_H_27_NO_6_: C, 74.28; H, 5.10; N, 2.63%. Found: C, 71.35; H, 5.17; N, 2.53%.

#### 3.2.14. (2S*,3R*,4*S,5S*)-Spiro[2,2′]-acenaphthene-1′-one-spiro[3,3″]chroman-4″-one-4-(*p*-bromophenyl)-5-carboxyethoxypyrrolidine (**9h**)

Yellow solid; Yield: (425 mg, 73%); mp (°C ± 2) = 226 °C; IR (KBr) υ: 3285, 2883, 1752, 1713, 1687, 1192, 792, 773 cm^−1^; ^1^H NMR (400 MHz, CDCl_3_) δ_H_: 1.11 (t, *J* = 7.1 Hz, 3H, CH_3_(ester)), 3.45 (d, *J* = 12.2 Hz, 1H, H-2″), 4.12–4.17 (m, 2H, CH_2_(ester)); 4.70 (d, *J* = 12,2 Hz, 1H, H-2″), 4.88 (d, *J* = 11.1 Hz, 1H, H-4), 5.08 (d, *J* = 11.1 Hz, 1H, H-5), 6.01 (d, *J* = 8.3 Hz, 1H, Ar-H), 6.85 (s, 1H, Ar-H), 6.57 (s, 1H, Ar-H), 7.29 (d, *J* = 6.8 Hz, 1H, Ar-H), 7.38–7.33 (m, 1H, Ar-H), 7.41 (m, 3H, Ar-H), 7.49 (d, *J* = 8.4 Hz, 2H, Ar-H), 7.54 (d, *J* = 8.2 Hz, 1H, Ar-H), 7.66–7.59 (m, 1H, Ar-H), 7.89 (m, 2H, Ar-H); ^13^C NMR (75 MHz, CDCl_3_) δ_C_: 14.0, 51.8, 61.3, 63.2, 70.9, 74.6, 116.1, 121.0, 121.7, 122.2, 125.2, 127.2, 127.9, 128.0, 130.8, 131.1, 131.7, 134.9, 160.0, 171.5, 191.4, 204.4. Anal. Calcd for C_32_H_24_BrNO_5_: C, 65.99; H, 4.15; N, 2.40%. Found: C, 65.90; H, 4.20; N, 2.55%.

### 3.3. Crystal Structure Determinations

X-ray suitable crystals were grown in EtOH. The crystal structure determination was accomplished on a Bruker D8 Venture four-circle diffractometer using a *PHOTON 100 CMOS* (**4a**, **9c**), or *PHOTON II CPAD* (**4e**), detector by *Bruker AXS GmbH*. X-ray radiation was generated by microfocus source *IμS*-Mo (λ = 0.71073 Å) by *Incoatec GmbH* with HELIOS mirror optics and a single-hole collimator by *Bruker AXS GmbH*. The crystals of **4a**, **4e**, and **9c** were covered with an inert oil (perfluoropolyalkylether) and mounted on a *MicroMount* from MiTeGen. For the data collection, the programs APEX 3 Suite (v.2018.7-2), with the integrated programs SAINT (integration) and SADABS (adsorption correction) by *BrukerAXS GmbH*, were used. The processing and finalization of the crystal structures was done with the program Olex2 [76]. The crystal structure was solved with the ShelXT [77] structure solution program using Intrinsic Phasing and refined with the ShelXL [78] refinement package using Least Squares minimization. The non-hydrogen atoms were refined anisotropically. The C-bound and H atoms were placed in geometrically calculated positions and each was assigned a fixed isotropic displacement parameter based on a riding-model: C–H =0.95–0.99 Å with U_iso_(H) = 1.5U_eq_(CH_3_) and 1.2U_eq_(CH_2_, CH) for other hydrogen atoms. The N-bound hydrogen atoms were located in the difference-Fourier-map and refined independently. The crystal and structure refinement data of **4a**, **4e**, and **9c** are gathered in Table S3. Crystallographic data for the structures of **4a**, **4e**, and **9c** have been deposited with the Cambridge Crystallographic Data Centre as supplementary publication number 2116000 (**4a**), 2116001 (**4e**), and 2115999 (**9c**). Copies of these data can be obtained, free of charge, on application to CCDC, 12 Union Road, Cambridge, CB2 IEZ, UK; fax: 144-(0)1223-336033 or e-mail: deposit@ccdc.cam.ac.uk.

### 3.4. Antimicrobial Activity

#### 3.4.1. Determination of the Antibacterial and Antifungal Activity

Various concentrations of the screened compounds have been used to determine their antimicrobial activity. Both cultures have been set to yield approximately 1 × 10^6^ CFUmL^−1^ of bacteria or yeast overnight. The minimum inhibitory concentrations (MICs) have been determined on brain heart infusion (BHI) agar plates (Bio-Rad, Marne la Coquette, France) by a standard method (Edziri et al., 2012). One milliliter of each of the extracts, previously dissolved in 10% dimethyl sulfoxide (DMSO), have been mixed for each concentration with 19 mL of BHI agar at 40 °C and poured over Petri dishes. The resulting DMSO concentration was approximately 0.5%. Plates containing only medium, or medium with 0.5% DMSO, were used as controls to ensure that DMSO did not affect the growth. Standard antibiotics Amoxicillin (AMX) and Ampicillin (AMP), and the antifungal Amphotericin B were used to control the sensitivity of the tested microorganism. After 18 h of incubation at 37 °C, the MIC was defined as the lowest concentration of the extract inhibiting the visible growth of each microorganism. Each test was carried out in triplicate.

#### 3.4.2. Microorganisms

The antibacterial potential of the dispiropyrrolidine derivatives has been investigated against nine microorganisms, namely the Gram-positive *Staphylococcus epidermidis* (*CI1232*), *Bacillus subtilis* (ATCC 6633), *Staphylococcus* aureus (ATCC 29213), *Staphylococcus aureus* (ATCC 25923), and *Enterococcus faecalis* (ATCC 29212) and the Gram-negative rods *Escherichia coli* (ATCC 25922), *Klebsiella pneumoniae* (ATCC 4352), *Salmonella enterica 800390*, and *Pseudomonas aeruginosa* (ATCC 9023). For the evaluation of the antifungal activity, *Candida glabrata* (ATCC 90030), *Candida albicans* (ATCC 90028), and *Candida* krusei (ATCC 6258) have been used. As references, to evaluate and to compare the potency of the tested compounds under the same conditions, the antibiotics Amoxicillin (AMX) and Ampicillin (AMP) were chosen as antibacterial agents. Macrocyclic Amphotericin B was used as antifungal reference (both of the previously identified bacterial and fungal strains were obtained from the laboratory of Transmissible Diseases and Biologically Active Substances, Faculty of Pharmacy, Tunisia).

### 3.5. Computational Methods

DFT calculations were performed using the Gaussian 16 suite of programs [64]. All geometry optimizations and frequency analyses were carried out using the *ω*B97xd functional with the standard 6-31G(d,p) basis set [58]. Vibrational analyses were performed to identify the stationary points as either local minima, or transition states (with one imaginary vibrational mode) on the potential energy surface. Solvent effects were considered using the polarizable continuum model (PCM) with acetonitrile as the solvent medium at a temperature of 355 K [59,60]. The frontier molecular orbitals analysis (FMOs) and molecular electrostatic potential (MEP) surfaces for compounds **4a**–**f** and **9a**–**h** were calculated at the same level of theory and visualized with the graphical interface Gauss View 6 [79] (Gaussian Inc., Wallingford, CT, USA). The chemical reactivity descriptors [80,81,82] (electronic chemical potential (μ), electronegativity (χ), chemical hardness (η), and electrophilicity index ω) are calculated as follows: *μ* = (E_H_ + E_L_)/2; η = (E_L_− E_H_)/2; ω = (μ^2^/2η).

## 4. Conclusions

A *one-pot* three-component 1,3-dipolar cycloaddition reaction of a stabilized azomethine ylide, generated in situ by thermal [1,2]-prototropy of the corresponding iminoesters with (*E*)-3-arylidene-thiochroman-4-one/chroman-4-one as dipolarophiles afforded the dispiro-oxindole-pyrrolidine-thiochroman-4-ones, **4a**–**f** and dispiro-oxindole-pyrrolidine-chroman-4-ones, **9a**–**h** in satisfactory yields along with high regio- and stereoselectivity. DFT calculations correctly predicted the observed experimental results, evidencing that the reaction is favorable according to kinetic and thermodynamic parameters. The antimicrobial activity of the novel *N*-heterocyclic compounds was examined against different pathogenic bacteria and fungi. According to the antimicrobial activity results, and on the basis of a structure-activity relationships (SAR) study, it follows that compounds **4a**–**d** containing a thiochromanone ring display a significant antimicrobial activity against most of the tested bacterial and fungal strains, superior than those of chromanone-grafted spiropyrrolidines **9a**–**h**. Even the activity of reference antibiotics is outperformed in some cases. Theoretical chemical reactivity, taking into account the FMOs and MEP maps, predicted a relatively higher chemical reactivity and softer character of spiropyrrolidines **4a**–**f**, which seems to have a prominent effect on their higher antimicrobial activity compared to the **9a**–**h** derivatives. The predicted absorption, distribution, metabolism, and excretion (ADME) profiles of some derivatives were in line with Lipinski rules. These promising results encourage further study of the antimicrobial activity of these derivatives by measuring their toxicity. Advanced docking studies are in progress and will be published elsewhere.

## Figures and Tables

**Table 1:** Synthesis of spiropyrrolidine-thiochroman-4-ones hybrids **4**.

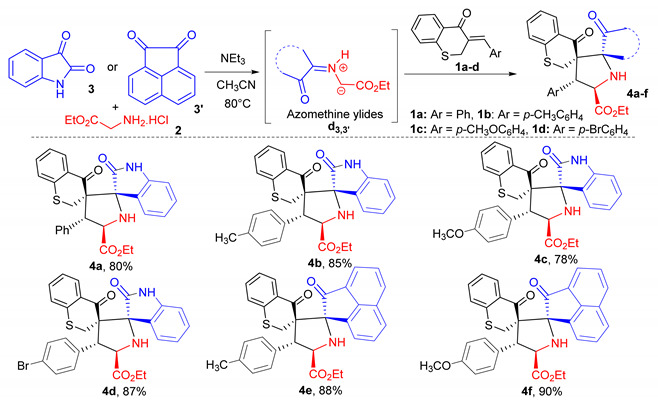

All reactions were carried out using **1** (1 mmol), **2** (1 mmol), **3**, or **3′** (1 mmol), NEt_3_ (1 mmol) in CH_3_CN (5.0 mL) at reflux for 2 h. Yields of the isolated cycloadducts are given.

**Table 2:** Synthesis of acenaphthylen-1(2*H*)-one-spiropyrrolidine-chromanone hybrids **9**.

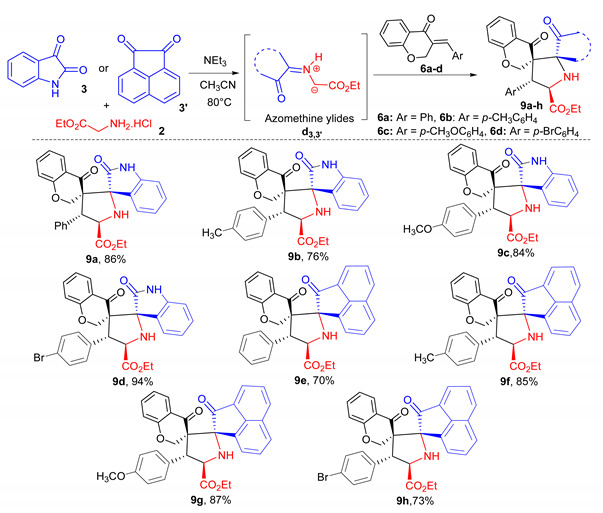

All reactions were carried out using **6** (1 mmol), **2** (1 mmol), **3**, or **3′** (1 mmol), NEt_3_ (1 mmol) in CH_3_CN (5.0 mL) at reflux for 2 h. Yields of the isolated cycloadducts are given.

**Table 3:** Difference in the Gibbs free energies (ΔG, in kcal mol^−1^), enthalpies (ΔH, in kcal mol^−1^), entropies (ΔS, in cal K^−1^ mol^−1^), and electronic energies (ΔU, in kcal mol^−1^) for TSs and products calculated at the *ω*B97xd/6-31G(d,p) level of theory.

	ΔG	ΔH	ΔS	ΔU
TS-*exo*-**4d**	0.4	−17.6	−50.9	−16.9
TS-*endo*-**4d**	2.0	15.4	−48.9	−14.7
TS-*exo*-**5d**	15.1	−3.6	−52.7	−2.9
TS-*endo*-**5d**	14.9	−0.3	−42.7	0.4
*exo-* **4d**	−21.8	−42.4	−58.1	−41.7
*endo-* **4d**	−14.6	−34.8	57.0	−34.2
*exo-* **5d**	−16.8	−37.9	−59.5	−37.2
*endo-* **5d**	−16.3	−36.2	−56.3	−35.5

**Table 4:** MIC values (in µg/mL) of the target compounds **4** and **9** against Gram-positive and Gram-negative bacteria activities.

	Gram Positive Bacteria					Gram Negative Bacteria			
Comp.	*B. subtilis* ATCC 6633	*S. epidermidis* *CI1232*	*S. aureus* ATCC 29213	*S. aureus* ATCC 25923	*E. faecalis* ATCC 29212	*E. coli* ATCC 25922	*K. pneumoniae* ATCC 4352	*S. enterica* *800390*	*P. aeruginosa* ATCC 9023
**4a**	32	32	64	32	32	125	64	64	64
**4b**	32	32	64	32	32	125	64	64	125
**4c**	32	32	64	64	32	125	64	64	64
**4d**	32	32	64	64	32	125	64	64	64
**4e**	32	32	64	32	64	250	64	125	64
**9a**	125	250	125	125	125	250	250	550	250
**9b**	125	250	250	250	125	125	125	550	125
**9c**	125	125	125	250	64	250	64	550	125
**9d**	64	64	500	125	64	125	64	550	125
**9e**	125	250	125	125	64	250	64	550	125
**9f**	64	64	125	125	125	125	250	550	250
**9g**	64	64	125	125	125	125	500	500	500
**9h**	64	64	125	125	250	250	500	500	500
**AMX** ^a^	64	-	-	-	1	256	256	-	256
**AMP** ^a^	-	78	50	50	-	-	-	100	-

^a^ Positive control.

**Table 5:** Antifungal activity of synthesized compounds **4** and **9** (MIC was determined in µg/mL).

Compound	*Candida albicans* ATCC 90028	*Candida glabrata* ATCC 90030	*Candida krusei* ATCC 6258
**4a**	64	64	64
**4b**	64	64	64
**4c**	64	64	64
**4d**	64	32	32
**4e**	64	64	64
**9a**	250	250	250
**9b**	250	125	125
**9c**	125	125	125
**9d**	125	125	250
**9e**	500	500	500
**9f**	125	250	125
**9g**	250	125	250
**9h**	125	250	250
**Amphotericin B** ^a^	500	500	500

^a^ Positive control.

**Table 6:** The HOMO, LUMO energies and molecular descriptors of compounds **4a**–**f**.

Compound	E_HOMO_	E_LUMO_	ΔE_gap_	ղ	μ	ω
	(eV)			(eV)		
**4a**	−7.463	−5.502	1.961	0.981	−6.483	21.422
**4b**	−7.462	−5.501	1.961	0.981	−6.482	21.415
**4c**	−7.459	−5.494	1.965	0.983	−6.477	21.338
**4d**	−7.462	−5.507	1.955	0.978	−6.485	21.501
**4e**	−7.497	−5.860	1.637	0.819	−6.679	27.234
**4f**	−7.496	−5.860	1.636	0.818	−6.678	27.259

**Table 7:** The HOMO, LUMO energies and molecular descriptors of compounds **9a**–**h**.

Compound	E_HOMO_	E_LUMO_	ΔE_gap_	ղ	μ	ω
	(eV)			(eV)		
**9a**	−8.386	−5.499	2.887	1.444	−6.943	16.692
**9b**	−8.387	−5.497	2.890	1.445	−6.942	16.675
**9c**	−8.380	−5.492	2.888	1.444	−6.936	16.658
**9d**	−8.387	−5.499	2.888	1.444	−6.943	16.692
**9e**	−8.449	−5.875	2.574	1.287	−7.162	19.928
**9f**	−8.447	−5.874	2.573	1.287	−7.161	19.922
**9g**	−8.426	−5.874	2.552	1.276	−7.150	20.032
**9h**	−8.448	−5.878	2.570	1.285	−7.163	19.964

**Table 8:** Results of Lipinski and Veber rules for the tested compounds.

Compound	4d	9d	9e	9c	4a	4e
lip_acc	6	7	6	8	6	5
lip_don	3	3	2	3	3	2
lip_druglike	1	1	1	1	1	0
lip_violation	1	1	1	0	0	2
logP(o/w)	4.46	3.83	4.81	2.99	3.67	5.75
Weight	564.48	548.41	504.56	499.54	485.58	534.66
TPSA	89.08	98.31	86.28	107.54	89.08	77.05
logS	−8.03	−7.06	−7.95	−6.02	−6.94	−9.4
vol	478.5	464.88	476	464.88	451.25	507.5
b_rotN	4	4	4	5	4	4
mr	14.37	13.8	14.32	13.68	13.61	15.34
a_hyd	27	26	29	26	26	31

## Data Availability

The data presented in this study are not available from the authors.

## Supplementary Materials

The following are available online, copies of ^1^H and ^13^C {^1^H} NMR spectra of compounds **4** and **9**, figures of the supramolecular interactions occurring in the crystalline states for **4a** and **9c**, DFT and Cartesian coordinates and energies.

## Sample Availability

Samples of the compounds are available from the authors.
