# Supplementary material for: Antimicrobial Activity and DFT Studies of a Novel Set of Spiropyrrolidines Tethered with Thiochroman-4-one/Chroman-4-one Scaffolds
Source: Molecules. 2022 Jan 18;27(3):582. doi: 10.3390/molecules27030582 (PMC8839074; doi:10.3390/molecules27030582)
Supplement: Supplementary file 1 [file molecules-27-00582-s001.zip › molecules-1462713-supplementary.pdf]

Antimicrobial Activity, *in silico* Molecular Docking and DFT Studies of Novel Set of Spiropyrrolidines Tethered with Thiochroman-4-one/Chroman-4-one Scaffolds

Nourhène Chouchène[1], Amani Toumi[1], Sarra Boudriga[1,*], Hayet Edziri[2], Mansour Sobeh[3], Mohamed A. O. Abdelfattah[4], Moheddine Askri[1], Michael Knorr[5,*], Carsten Strohmann[6], Lukas Brieger[6], and Armand Soldera[7,*]

[1] Laboratory of Heterocyclic Chemistry Natural Product and Reactivity (LR11ES39), Department of Chemistry, Faculty of Science of Monastir, 5019 Monastir, Tunisia; nourhene.chouchene23@gmail.com (N.C.); amanitoumi45@gmail.com (A.T.); sarra_boudriga@yahoo.fr (S.B.); moheddine.askri@fsm.rnu.tn (M.A.)
[2] Laboratoire des maladies transmissibles et des substances biologiquement actives, Faculté de pharmacie, 5000 Monastir, Tunisia ; jaziri_hayet@yahoo.fr (H.E.)
[3] AgroBioSciences Research, Mohammed VI Polytechnic University, Lot 660–Hay MoulayRachid, Ben-Guerir 43150, Morocco; mansour.sobeh@um6p.ma (M.S.)
[4] College of Engineering and Technology, American University of the Middle East, Kuwait; Mohamed.AbdelMoety@aum.edu.kw (M.O.M.)
[5] Institut UTINAM-UMR CNRS 6213, Université Bourgogne Franche-Comté, 16 Route de Gray, 25030 Besançon, France ; michael.knorr@univ-fcomte.fr (M.K.)
[6] Technische Universität Dortmund, Anorganische Chemie, Otto-Hahn-Strasse 6, 44227 Dortmund, Germany; carsten.strohmann@tu-dortmund.de (C.S.); lukas.brieger@tu-dortmund.de (L.B.)
[7] Department of Chemistry, Laboratory of Physical Chemistry of Matter, Université de Sherbrooke, Sherbrooke, Québec J1K 2R1, Canada; Email: armand.soldera@usherbrooke.ca (A.S.)
*Correspondance: sarra_boudriga@yahoo.fr (S.B.); michael.knorr@univ-fcomte.fr (M.K.); armand.soldera@usherbrooke.ca (A.S.)

Supplementary Materials

Table of contents

		Page
**1.**	Copies of ^{1}H- and ^{13}C-NMR Spectra of compounds **4a-f** and **9a-h** (Fig. **S1** to **S30**)	2-16
**2.**	The chemical shifts δ [ppm] from the experimental and theoretical ^{1}H and ^{13}C NMR spectra of compounds **4a** and **9c.**	17
**3.**	Crystallographic data of **4a**, **4e**, and **9c**.	17-19
**4.**	DFT data	20-41

1. Copies of ^{1}H- and ^{13}C-NMR Spectra of compounds 4a-f and 9a-h

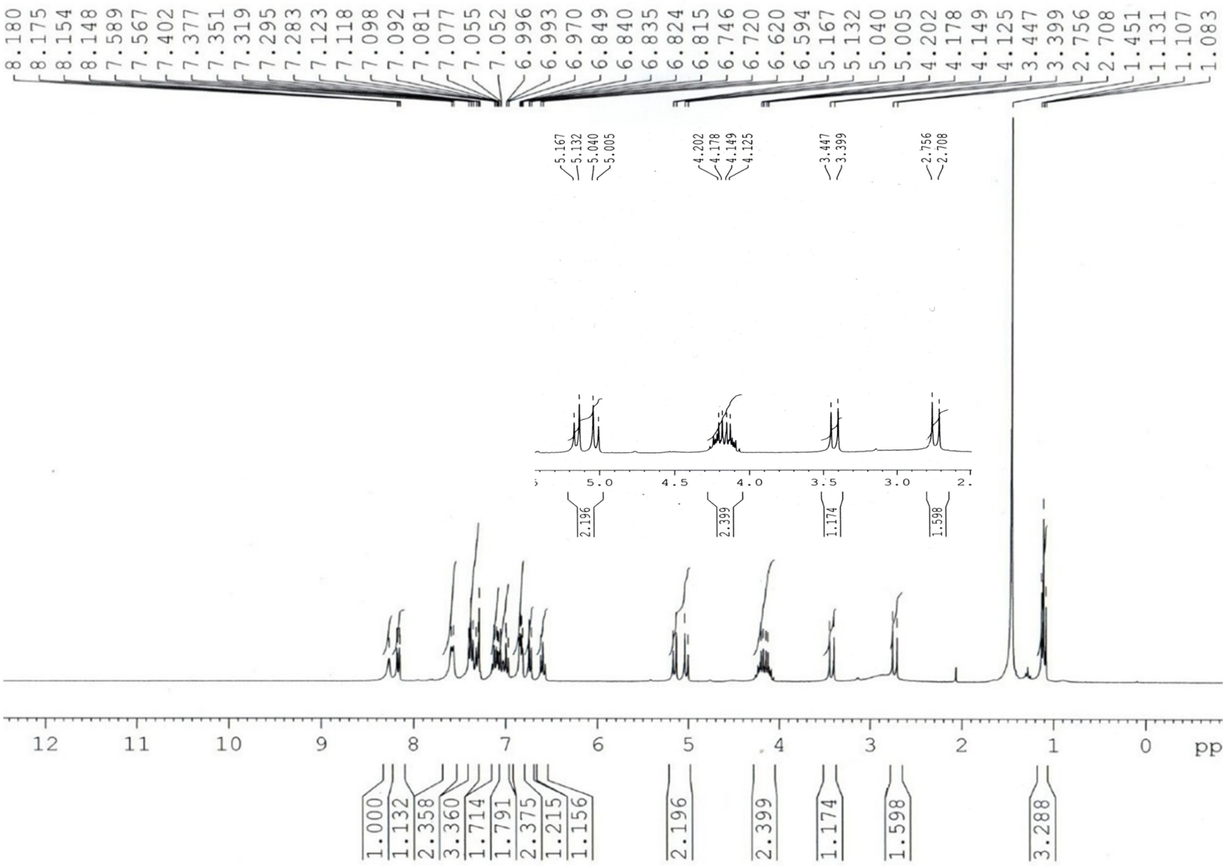

Figure S1. ^{1}H NMR spectrum of compound **4a** (300 MHz, $CDCl_3$)

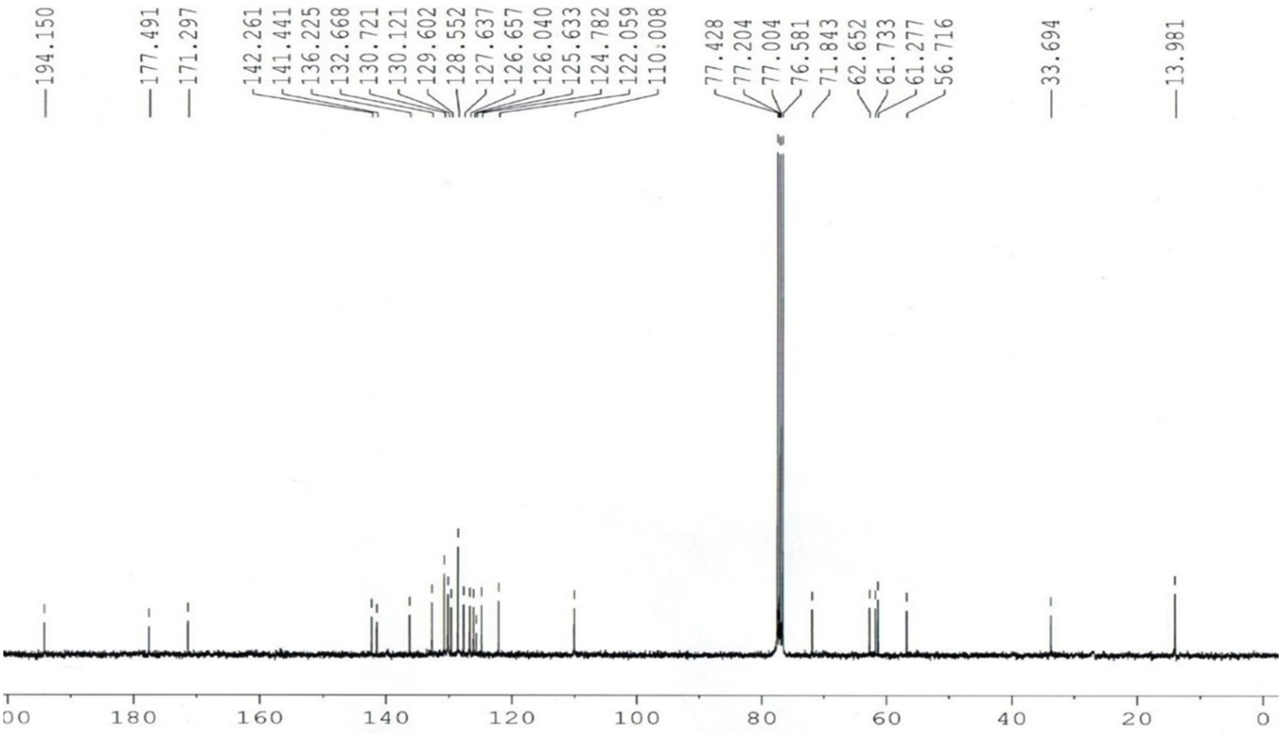

Figure S2. ^{13}C {^{1}H} NMR spectrum of compound **4a** (75 MHz, $CDCl_3$)

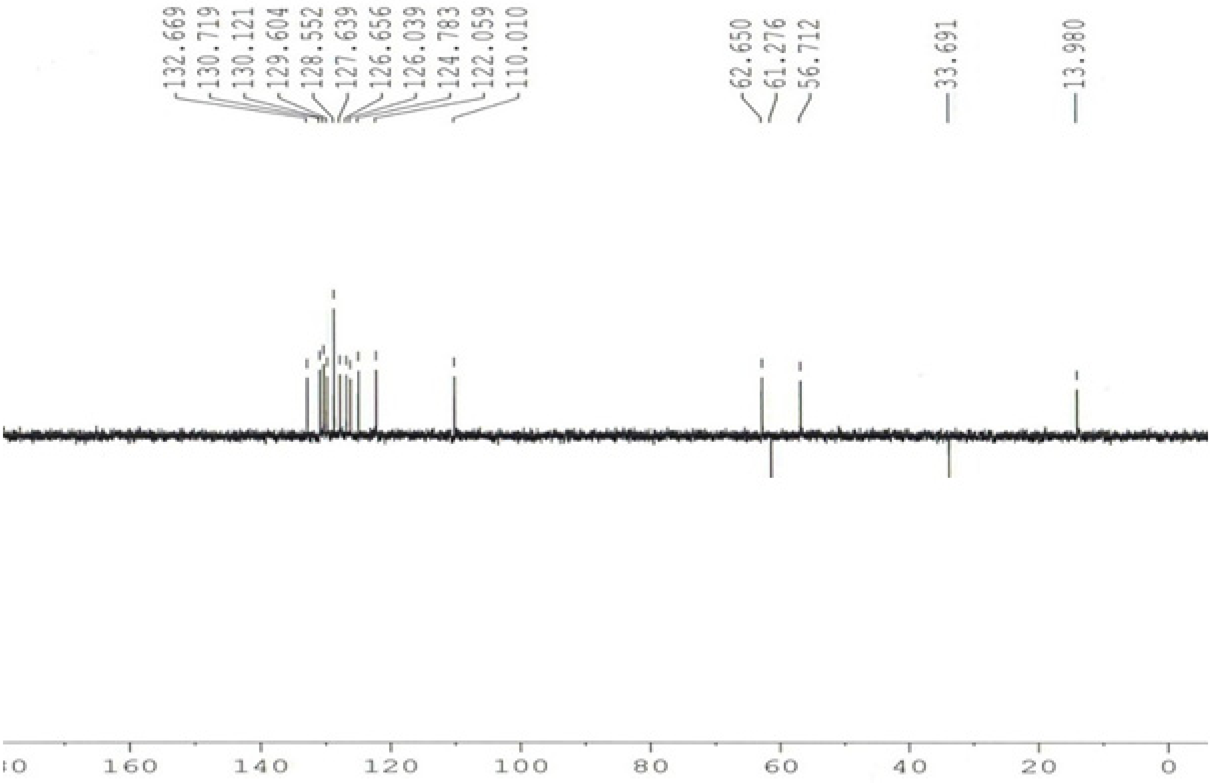

Figure S3. DEPT-135 ^{13}C NMR spectrum of compound **4a** (75 MHz, $CDCl_3$)

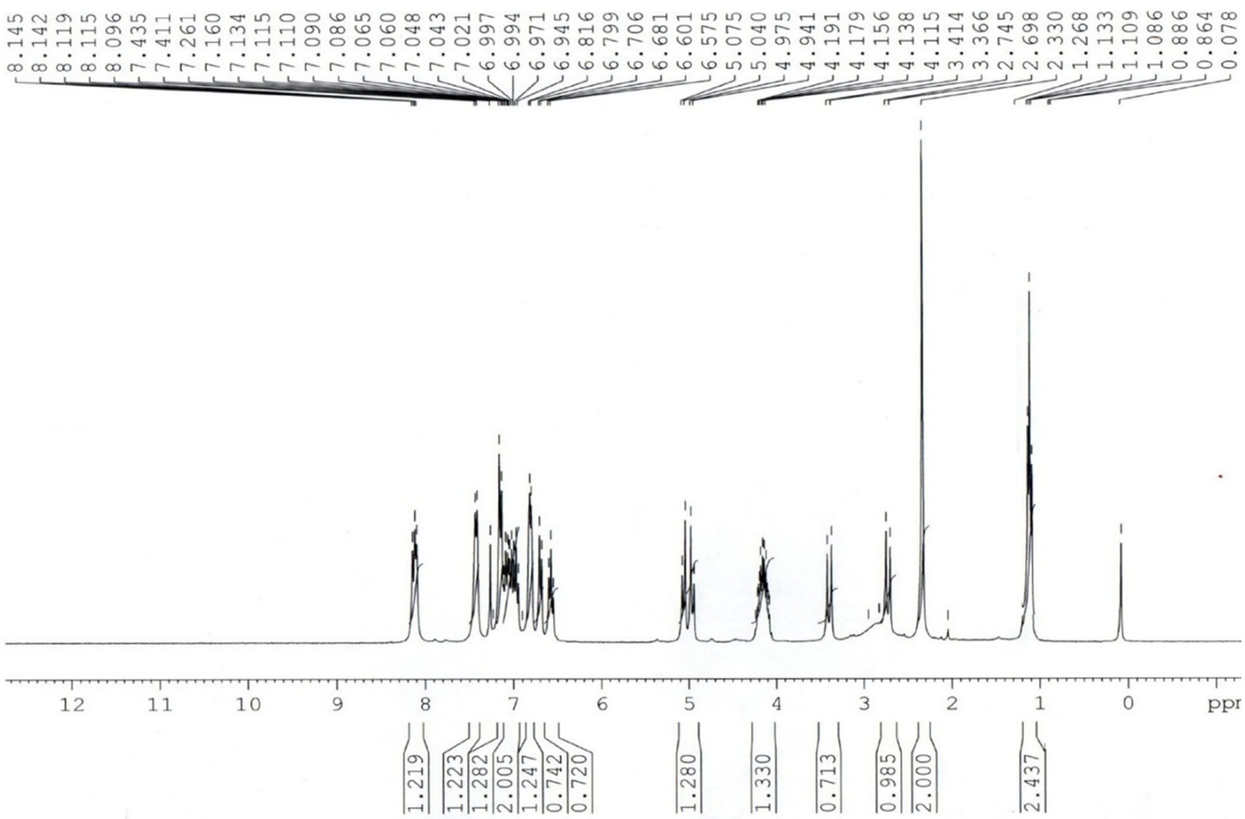

Figure S4. ^1H NMR spectrum of compound **4b** (300 MHz, $CDCl_3$)

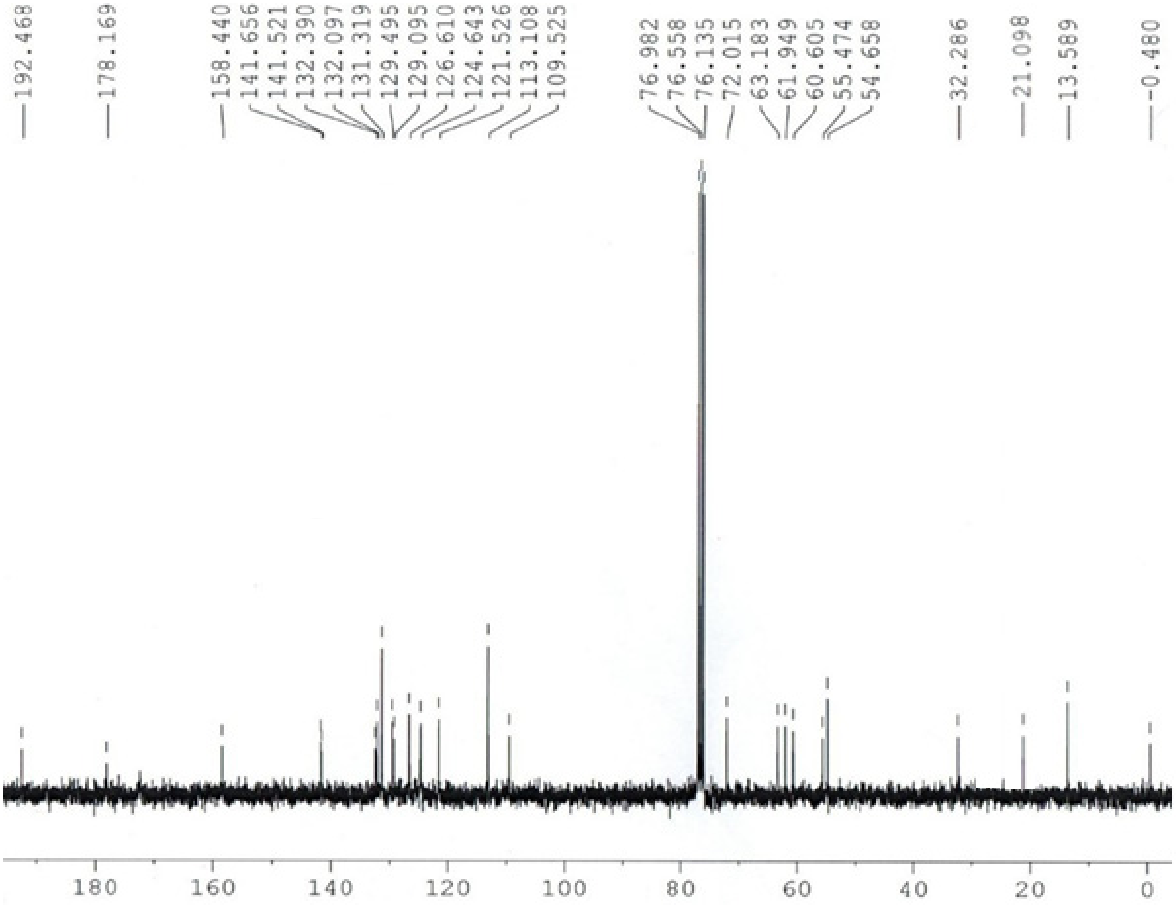

Figure S5. ^{13}C {^1H} NMR spectrum of compound **4b** (75 MHz, $CDCl_3$)

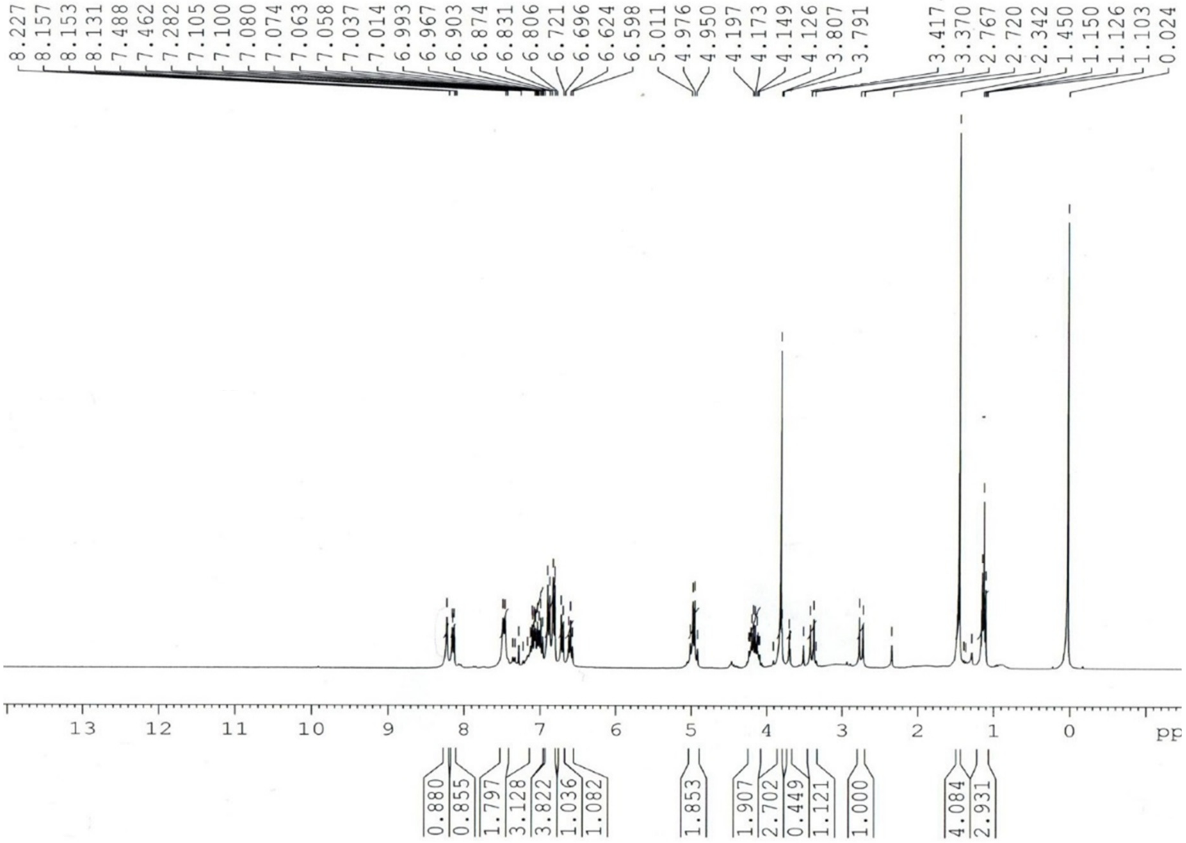

Figure S6. ^1H NMR spectrum of compound **4c** (300 MHz, $CDCl_3$)

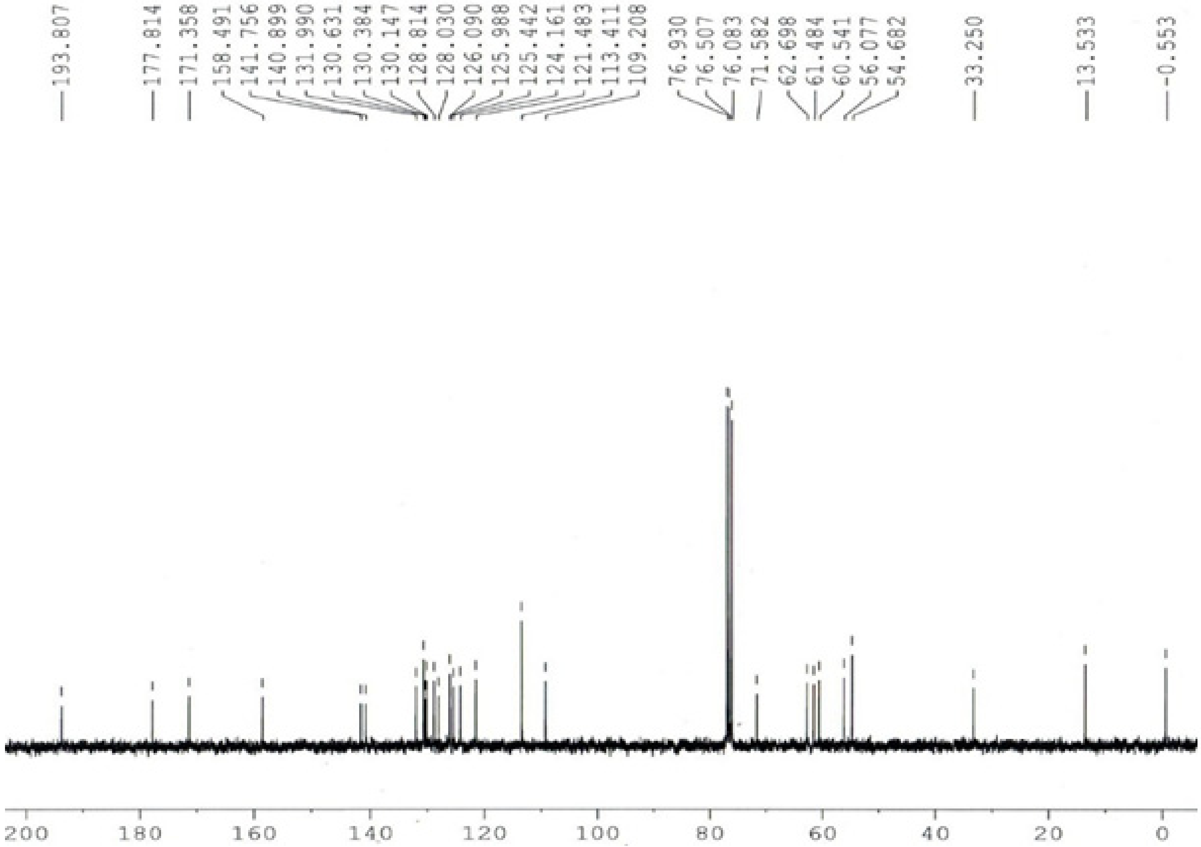

Figure S7. ^{13}C {^{1}H} NMR spectrum of compound **4c** (75 MHz, $CDCl_3$)

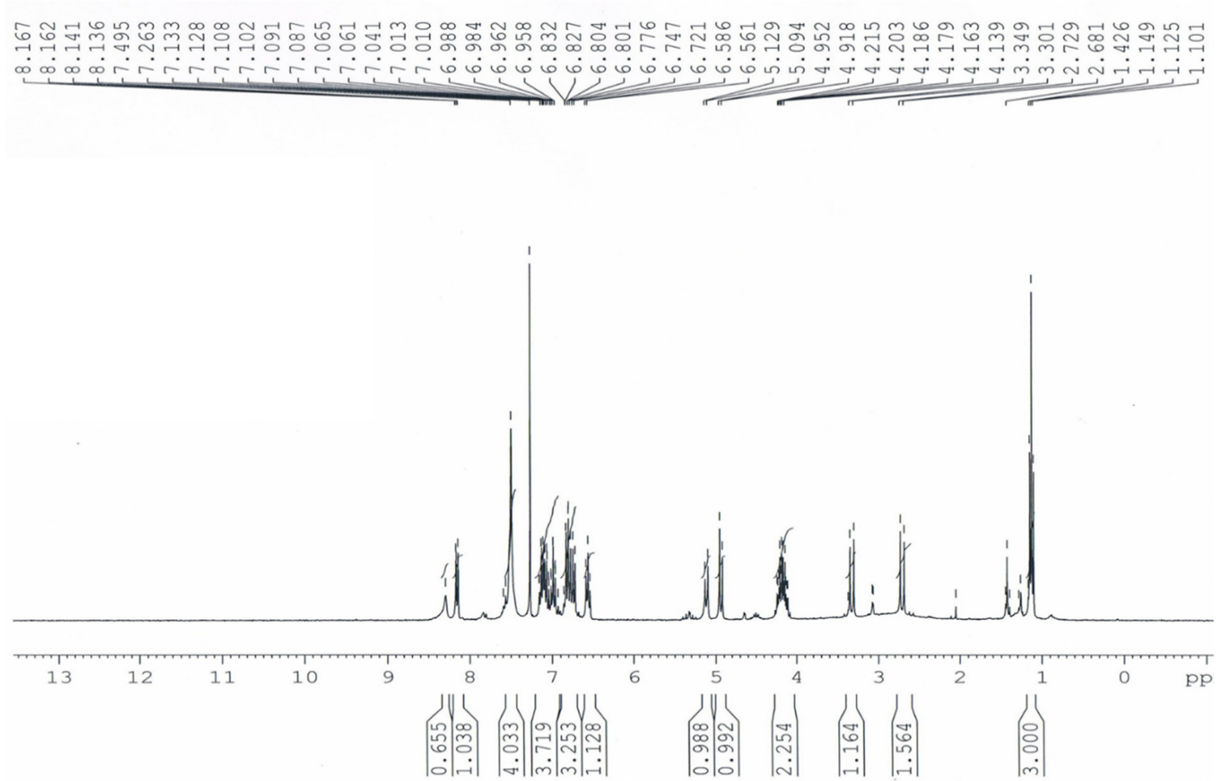

Figure S8. ^{1}H NMR spectrum of compound **4d** (300 MHz, $CDCl_3$)

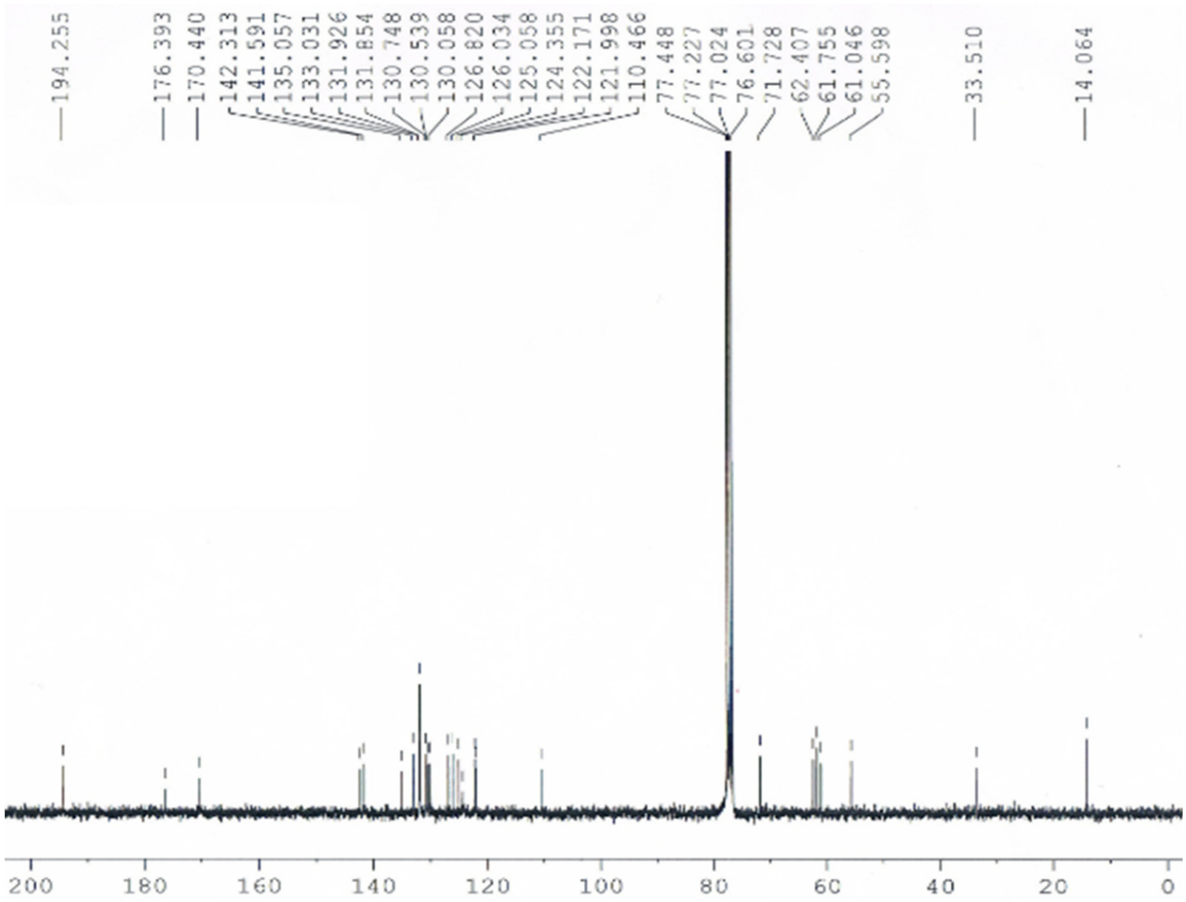

Figure S9. ^{13}C {^1H} NMR spectrum of compound **4d** (75 MHz, $CDCl_3$)

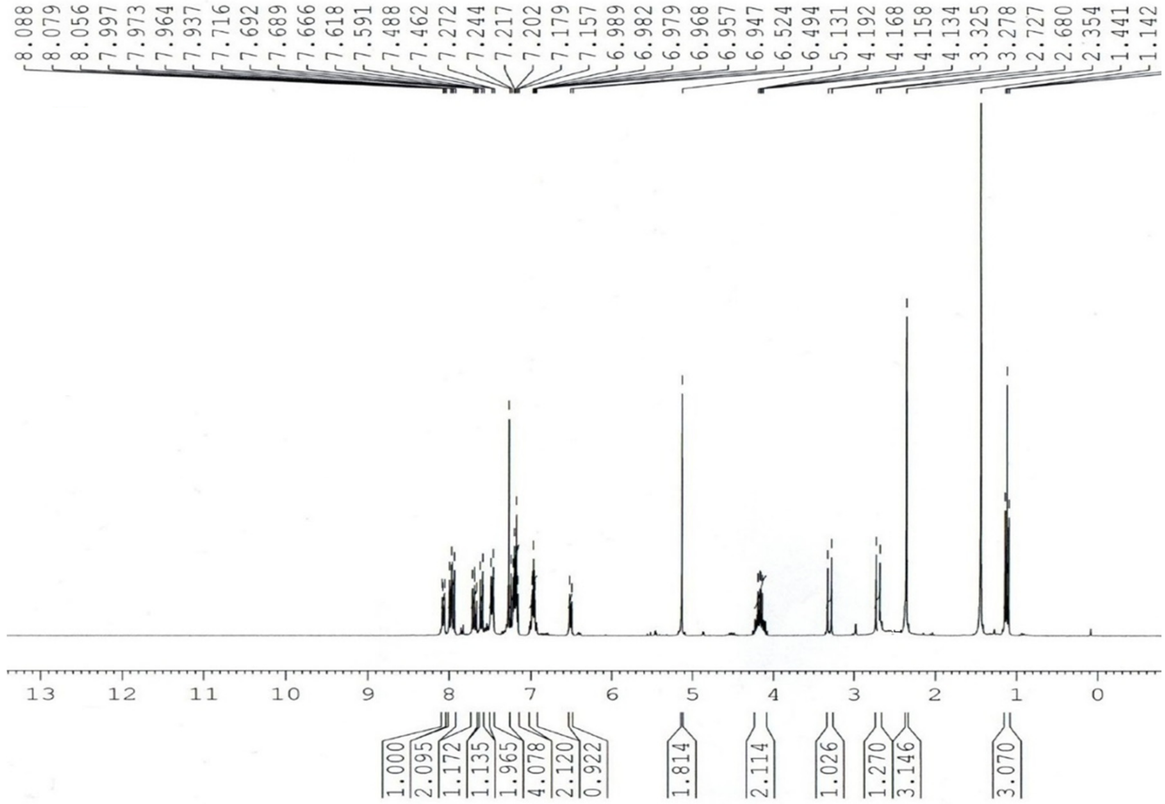

Figure S10. ^1H NMR spectrum of compound **4e** (300 MHz, $CDCl_3$)

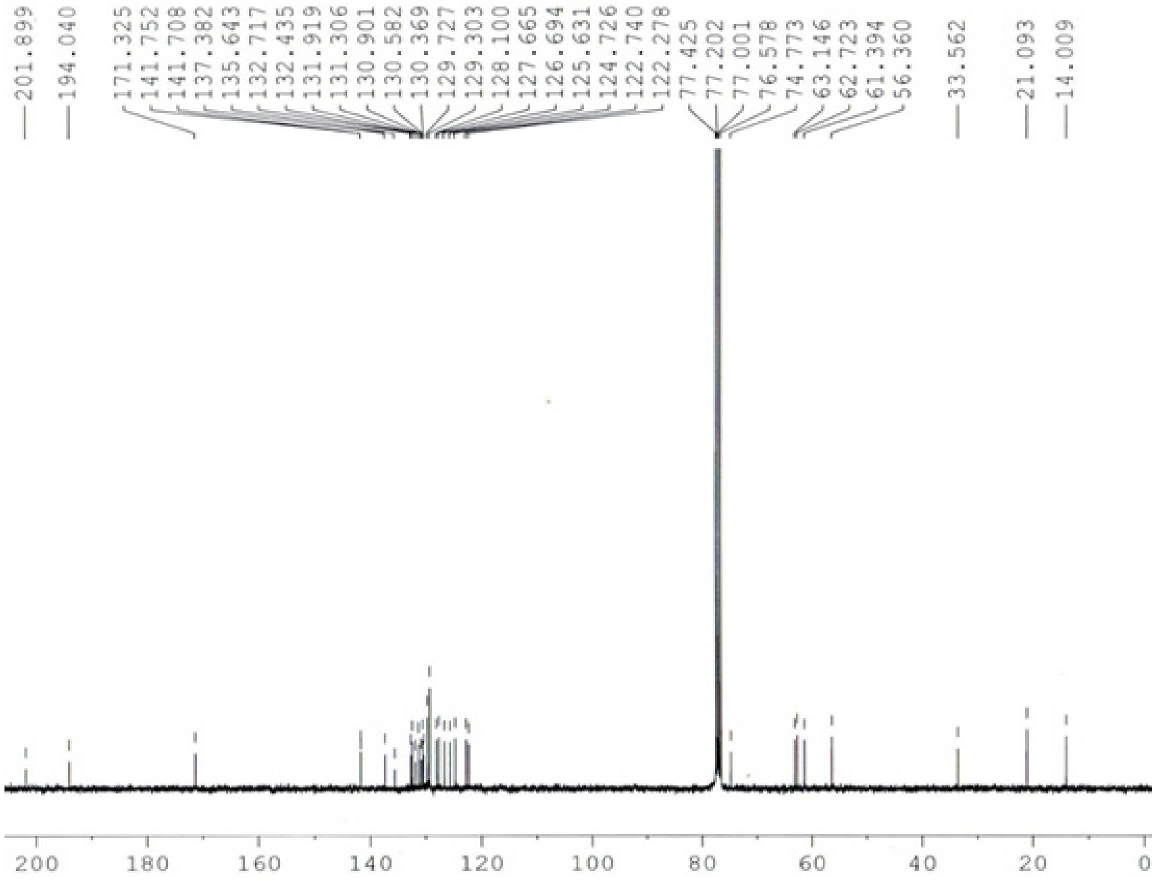

Figure S11. ^{13}C {^{1}H} NMR spectrum of compound **4e** (75 MHz, $CDCl_3$)

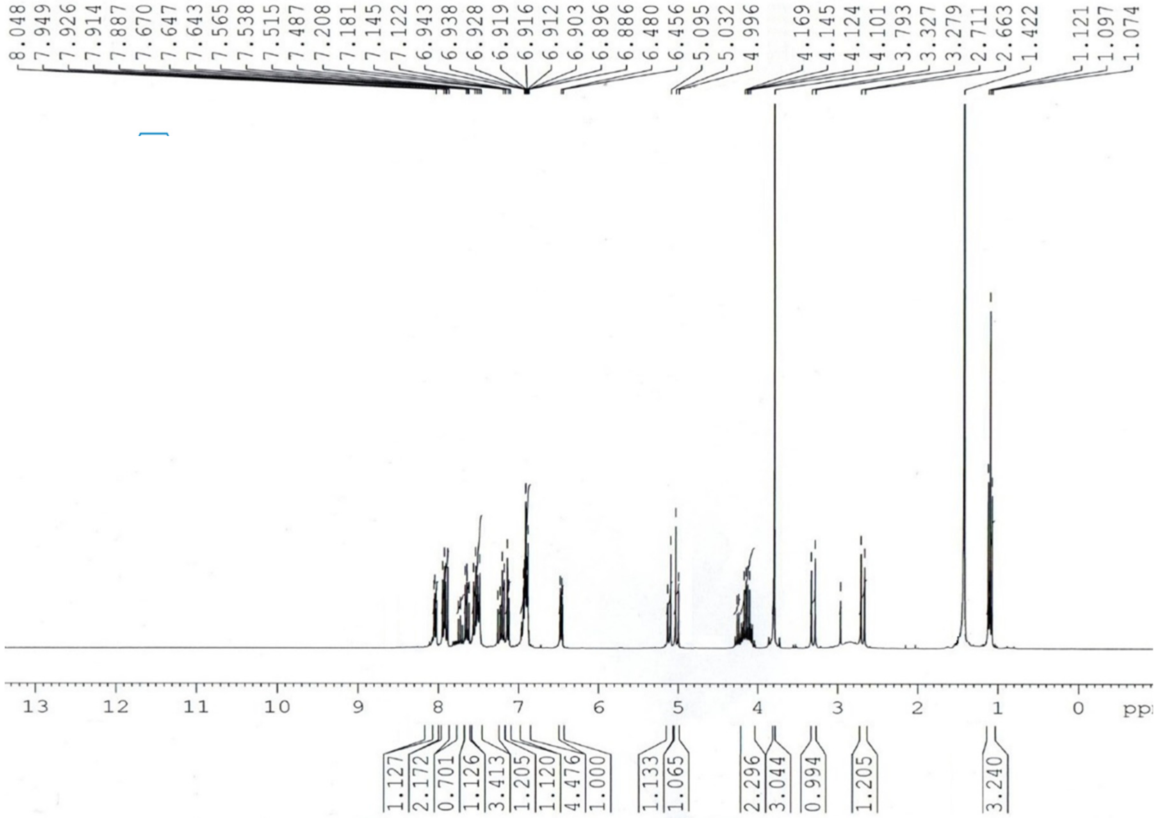

Figure S12. ^{1}H NMR spectrum of compound **4f** (300 MHz, $CDCl_3$)

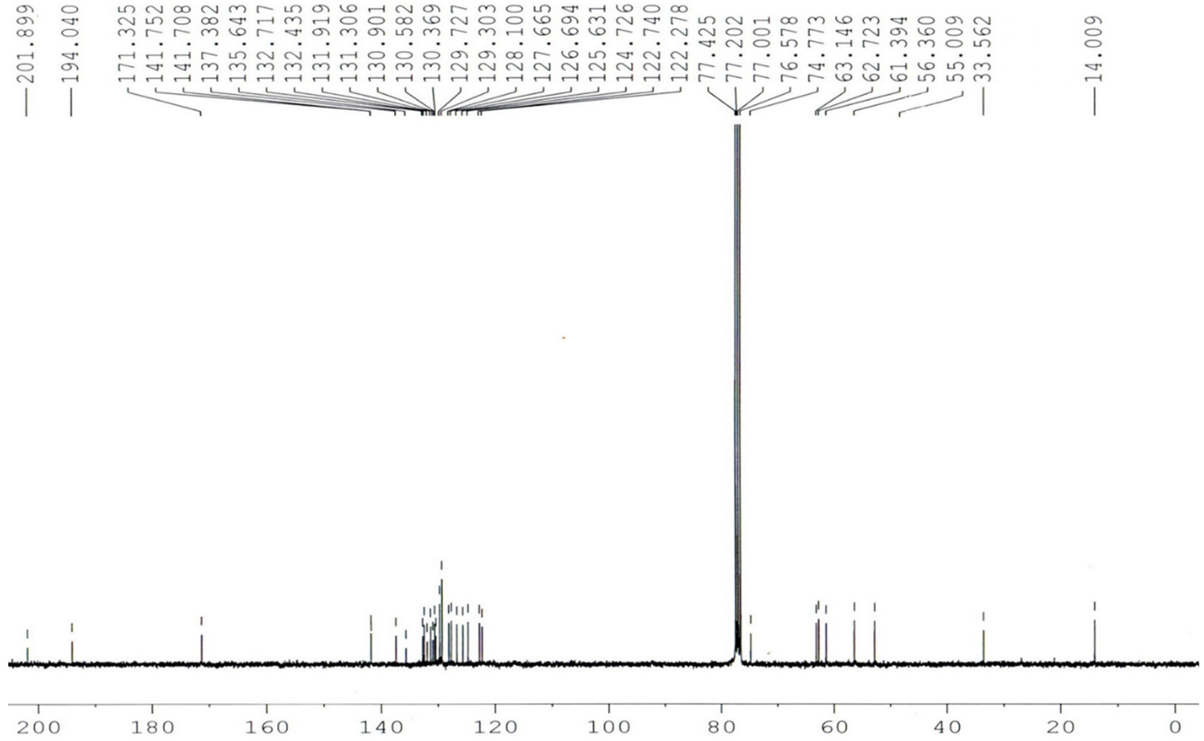

Figure S13. ^{13}C {^{1}H} NMR spectrum of compound **4f** (75 MHz, $CDCl_3$)

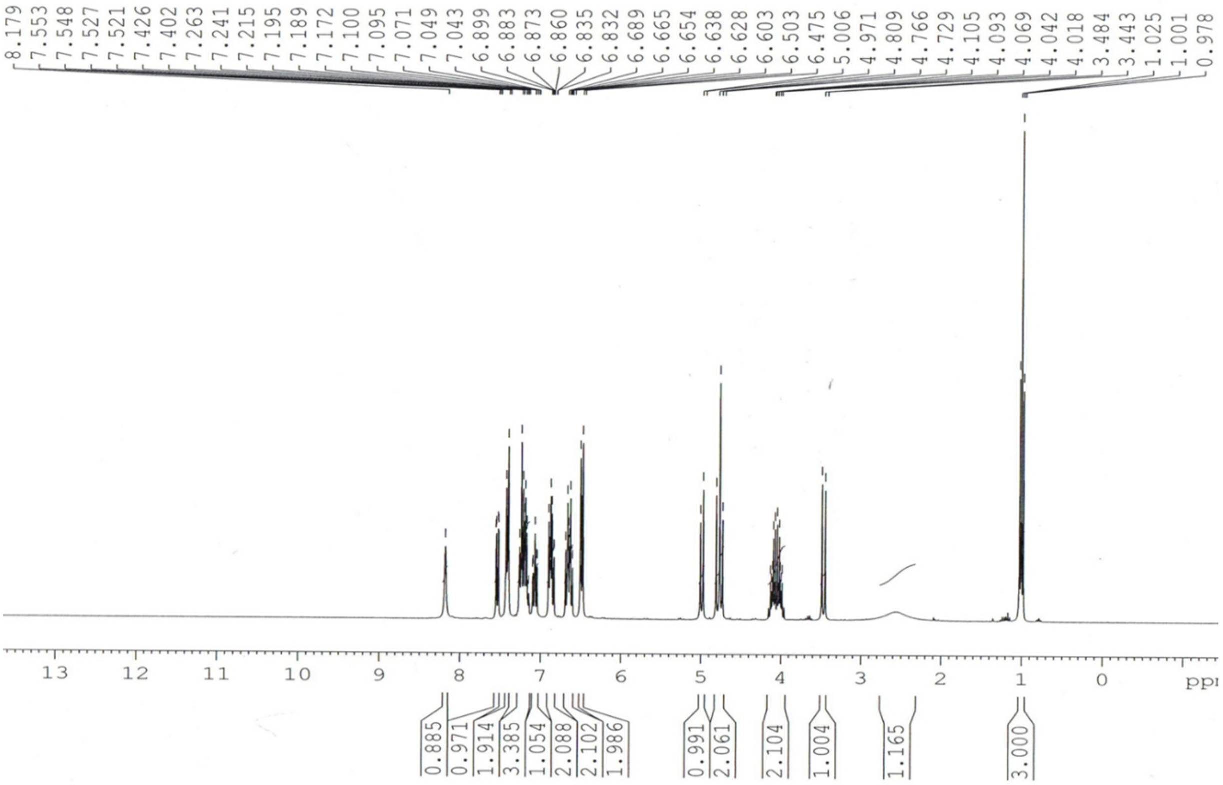

Figure S14. ^{1}H NMR spectrum of compound **9a** (300 MHz, $CDCl_3$)

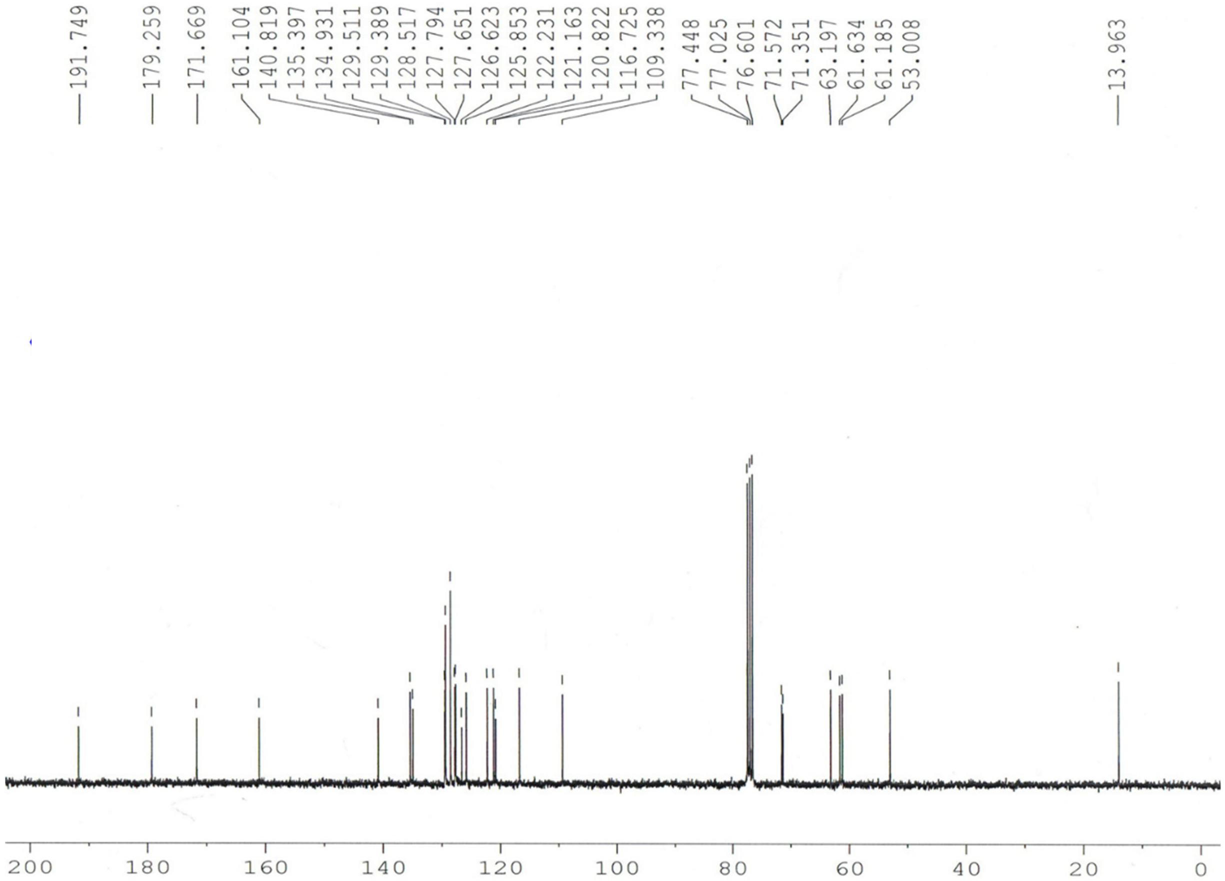

Figure S15. $^{13}C\ \{^{1}H\}$ NMR spectrum of compound **9a** (75 MHz, $CDCl_3$)

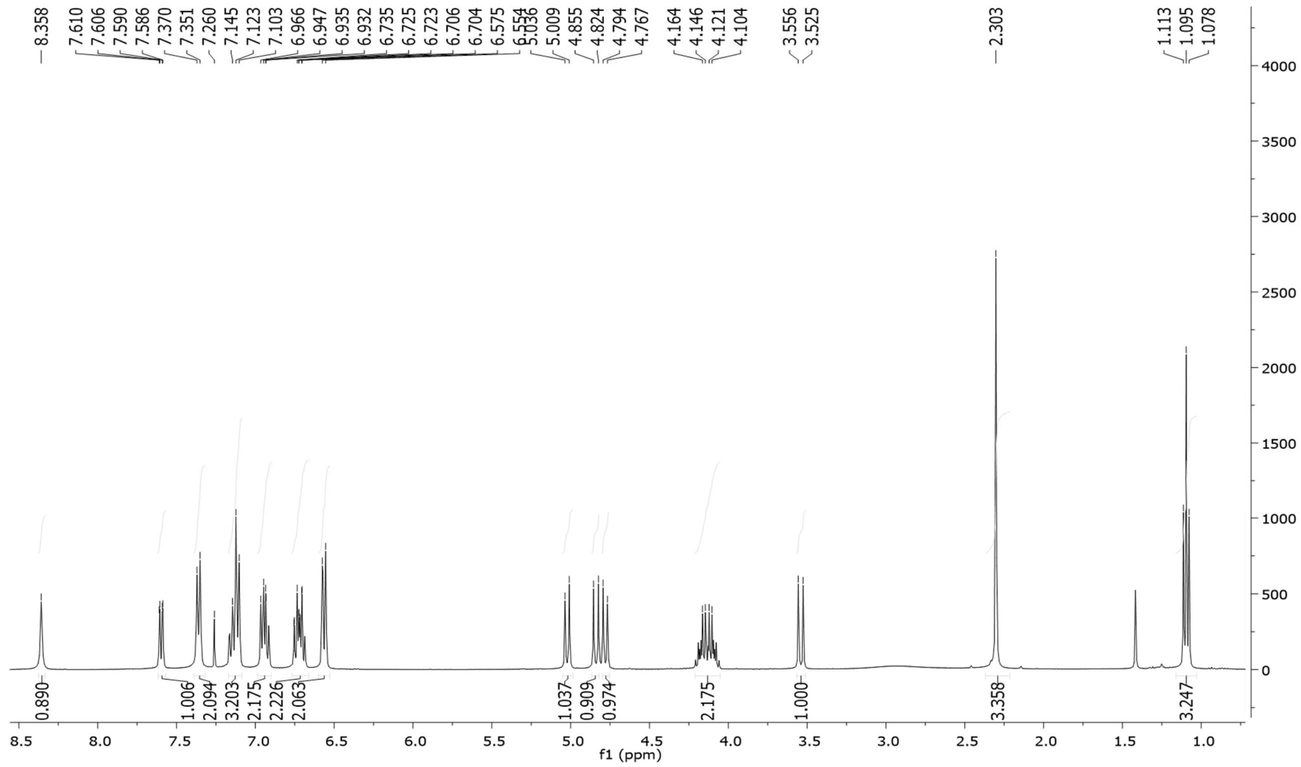

Figure S16. ^{1}H NMR spectrum of compound **9b** (400 MHz, $CDCl_3$)

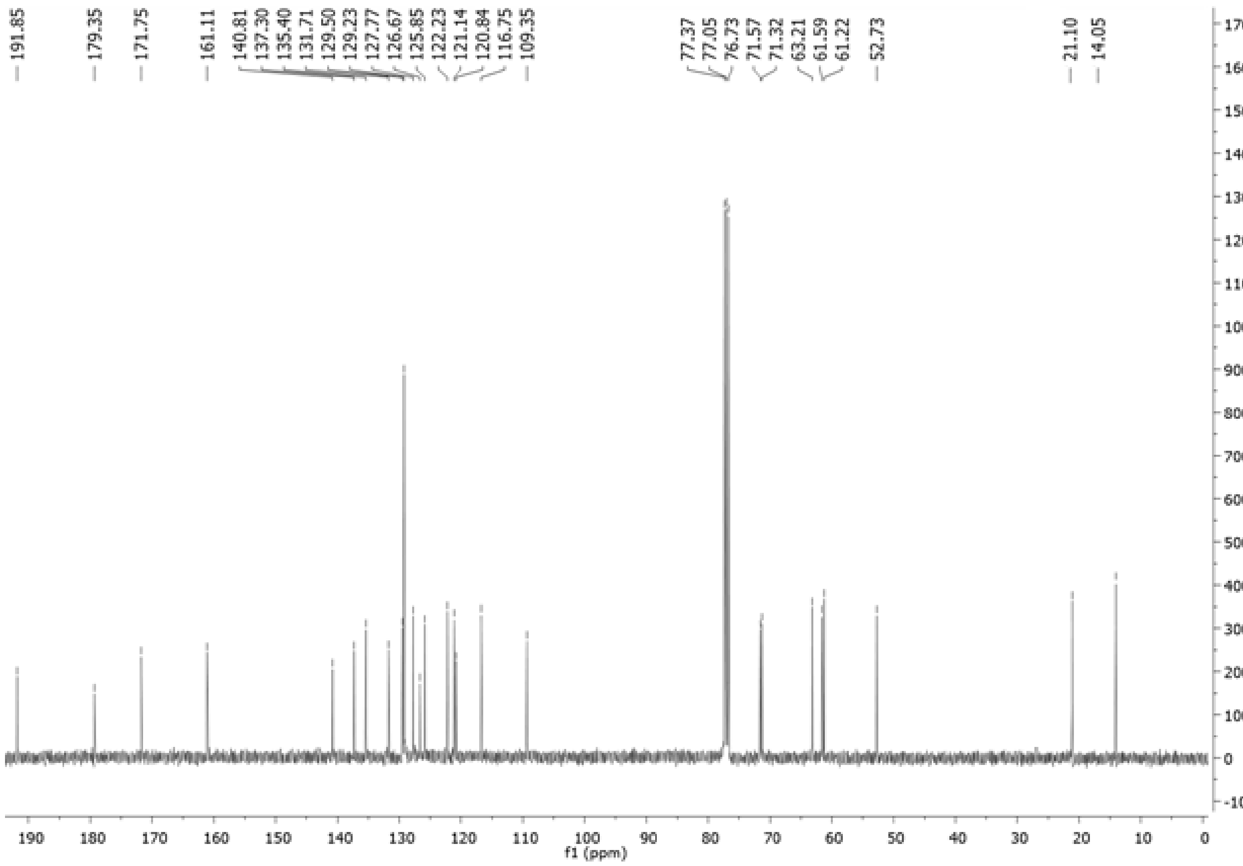

Figure S17. ^{13}C {^{1}H} NMR spectrum of compound **9b** (100 MHz, $CDCl_3$)

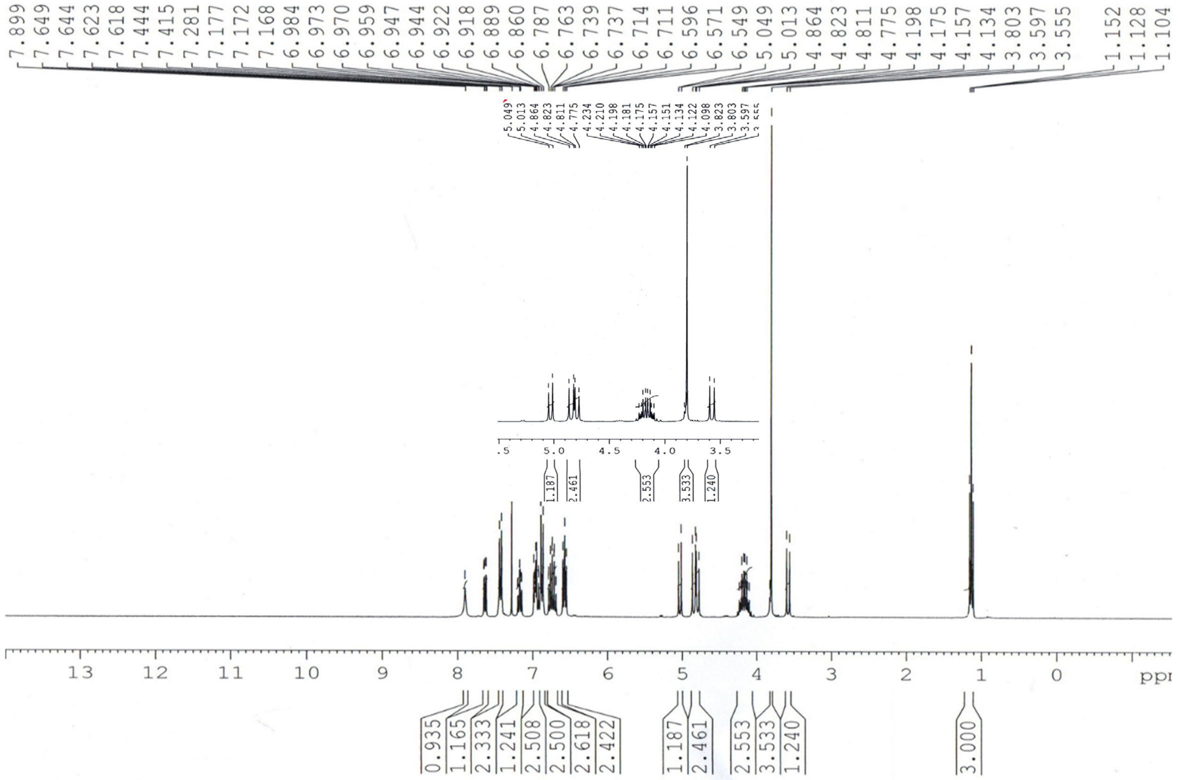

Figure S18. ^{1}H NMR spectrum of compound **9c** (300 MHz, $CDCl_3$)

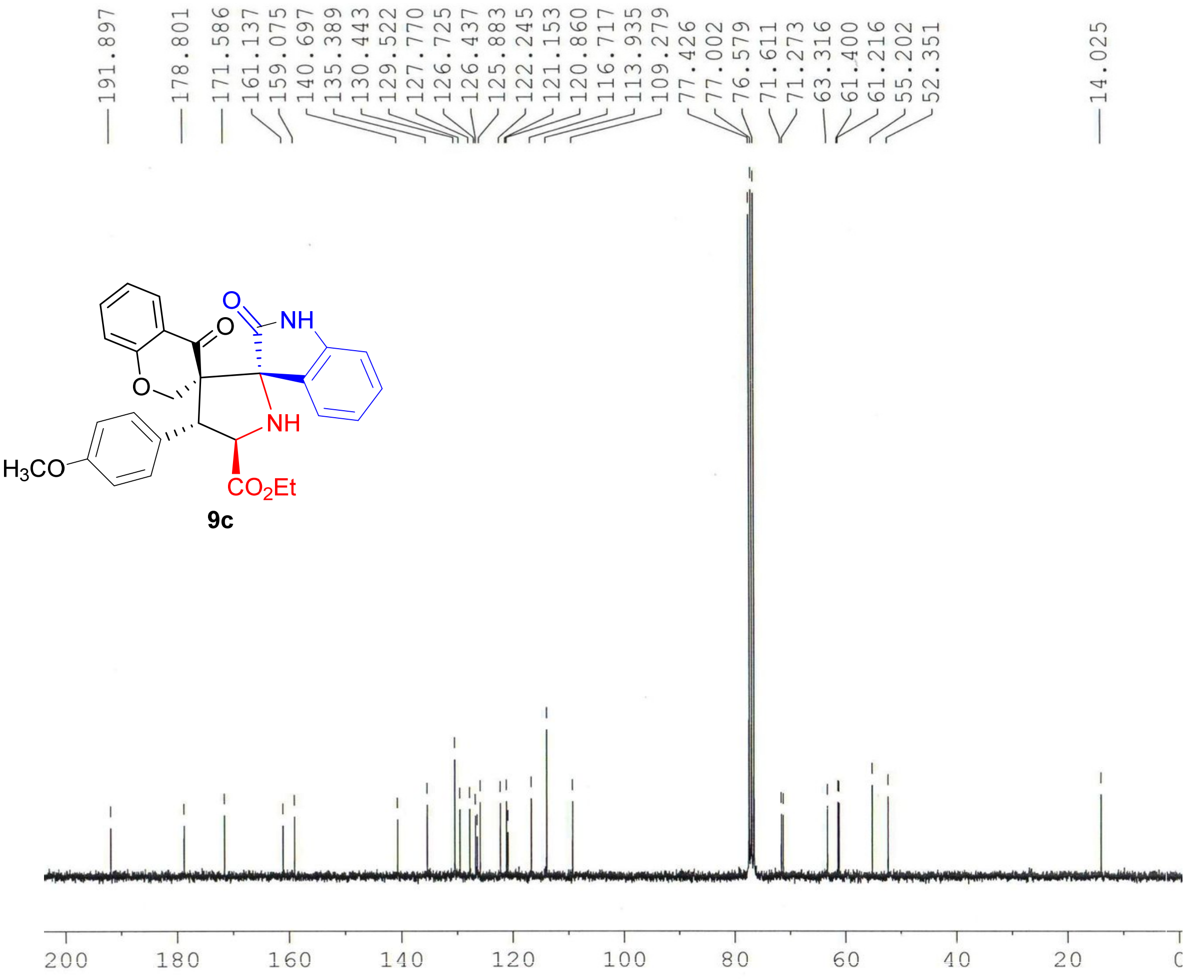

Figure S19. ^{13}C {^{1}H} NMR spectrum of compound **9c** (75 MHz, $CDCl_3$)

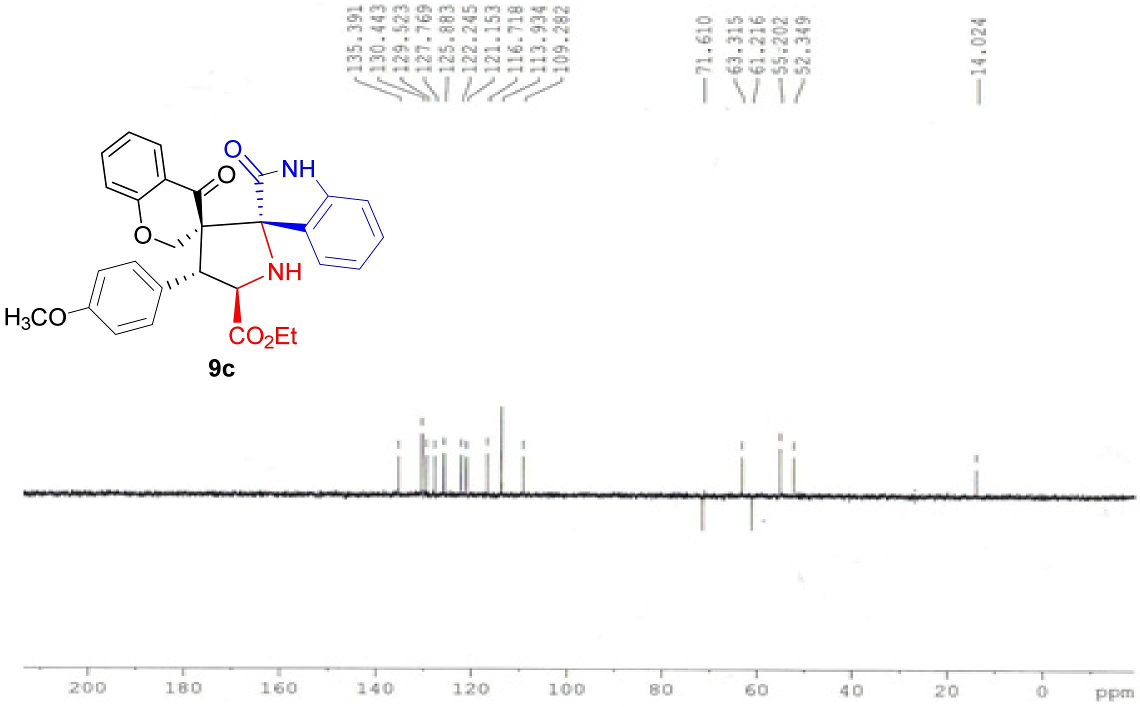

Figure S20. DEPT-135 ^{13}C NMR spectrum of compound **9c** (75 MHz, $CDCl_3$)

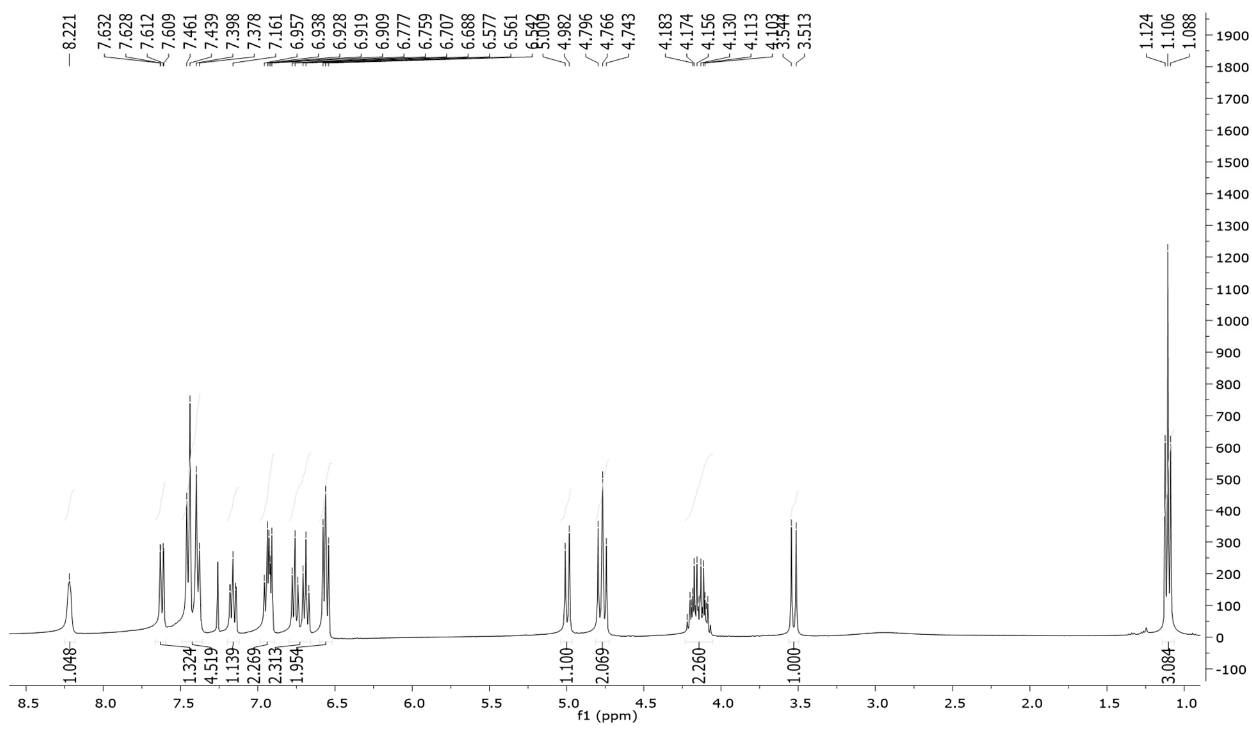

Figure S21. ^{1}H NMR spectrum of compound **9d** (400 MHz, $CDCl_3$)

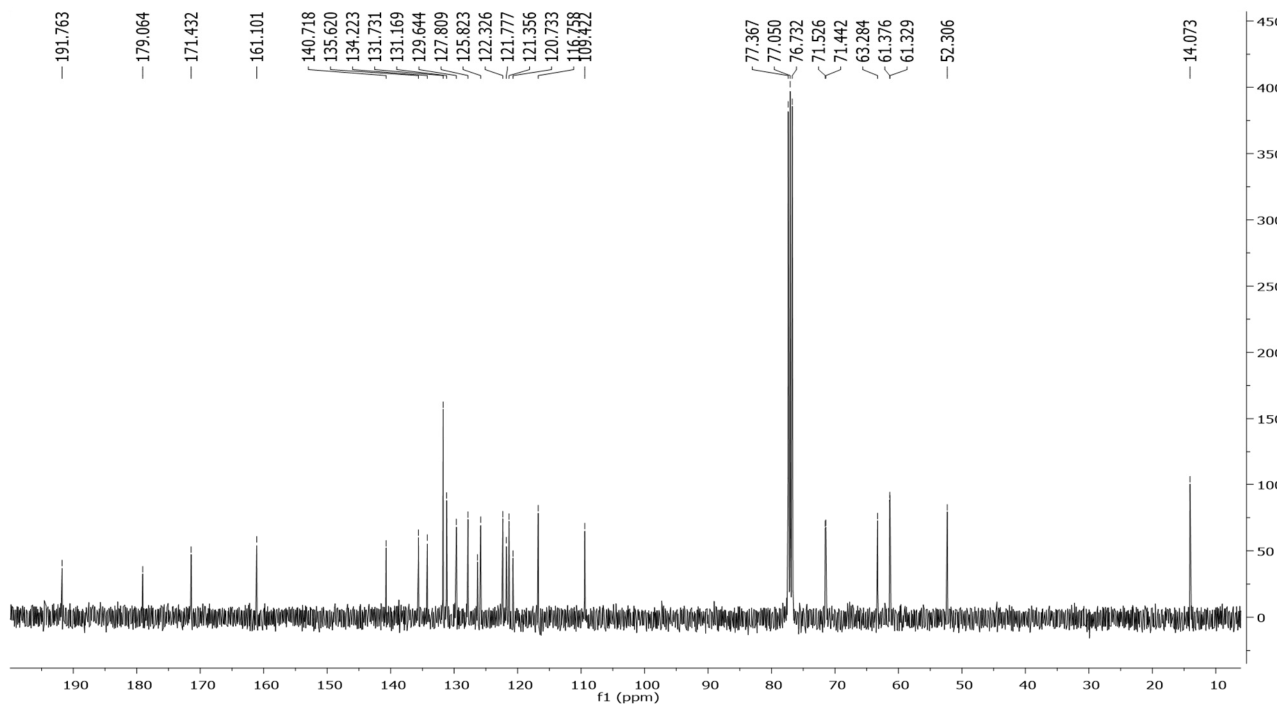

Figure S22. ^{13}C {^{1}H} NMR spectrum of compound **9d** (100 MHz, $CDCl_3$)

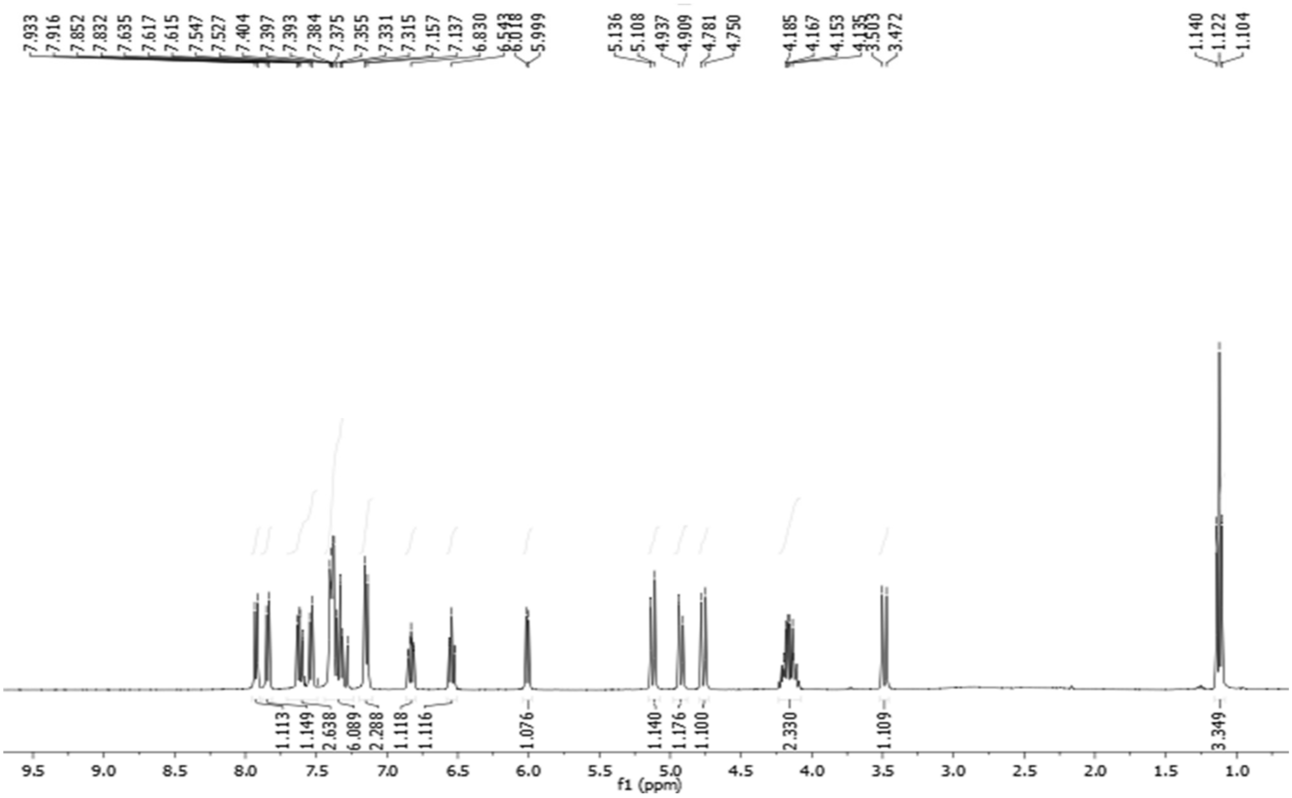

Figure S23. ^{1}H NMR spectrum of compound **9e** (400 MHz, $CDCl_3$)

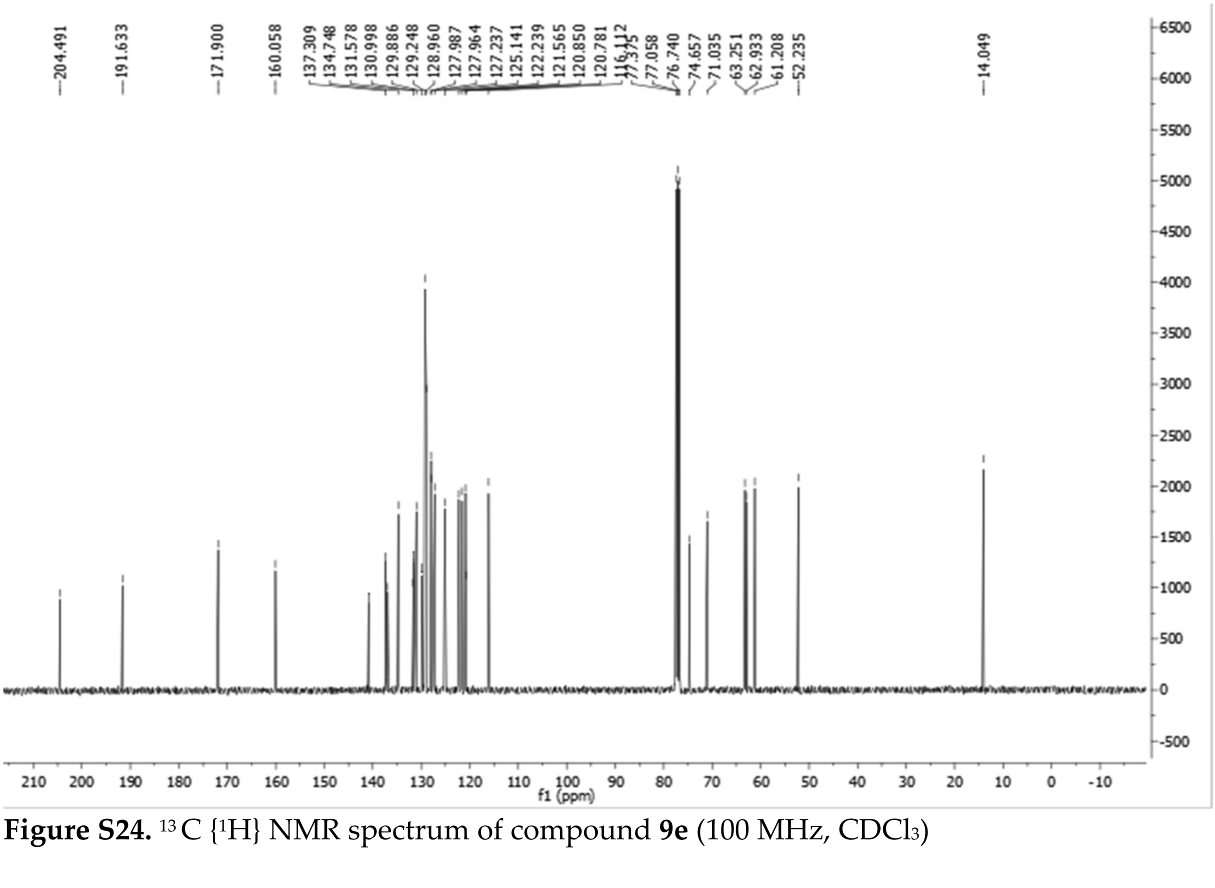

Figure S24. ^{13}C {^{1}H} NMR spectrum of compound **9e** (100 MHz, $CDCl_3$)

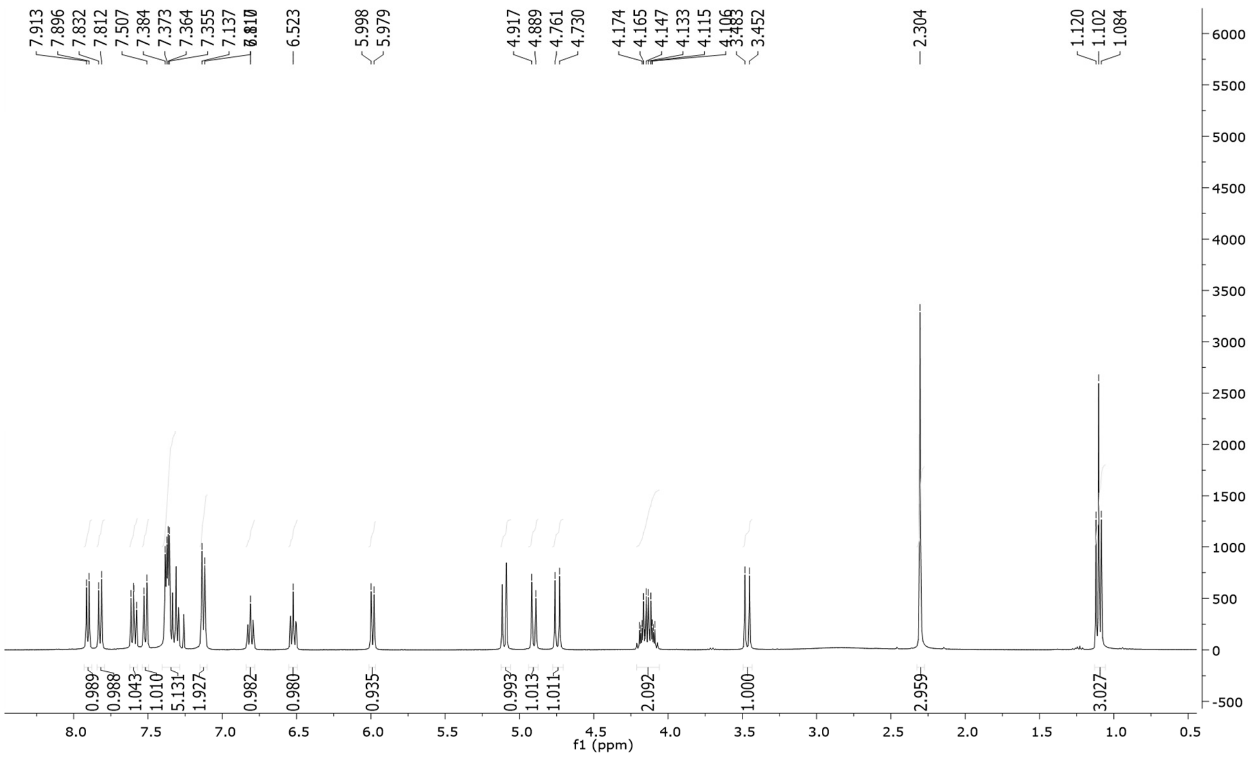

Figure S25. ^{1}H NMR spectrum of compound **9f** (400 MHz, $CDCl_3$)

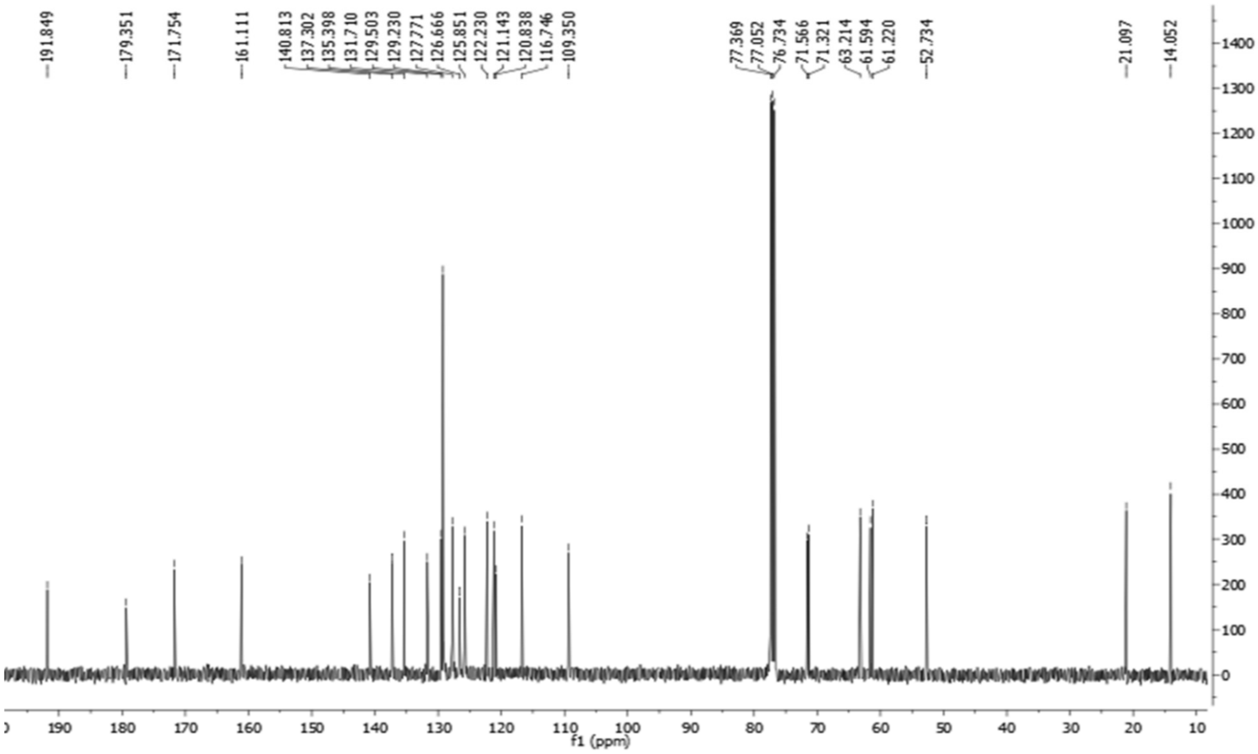

Figure S26. ^{13}C {^{1}H} NMR spectrum of compound **9f** (100 MHz, $CDCl_3$)

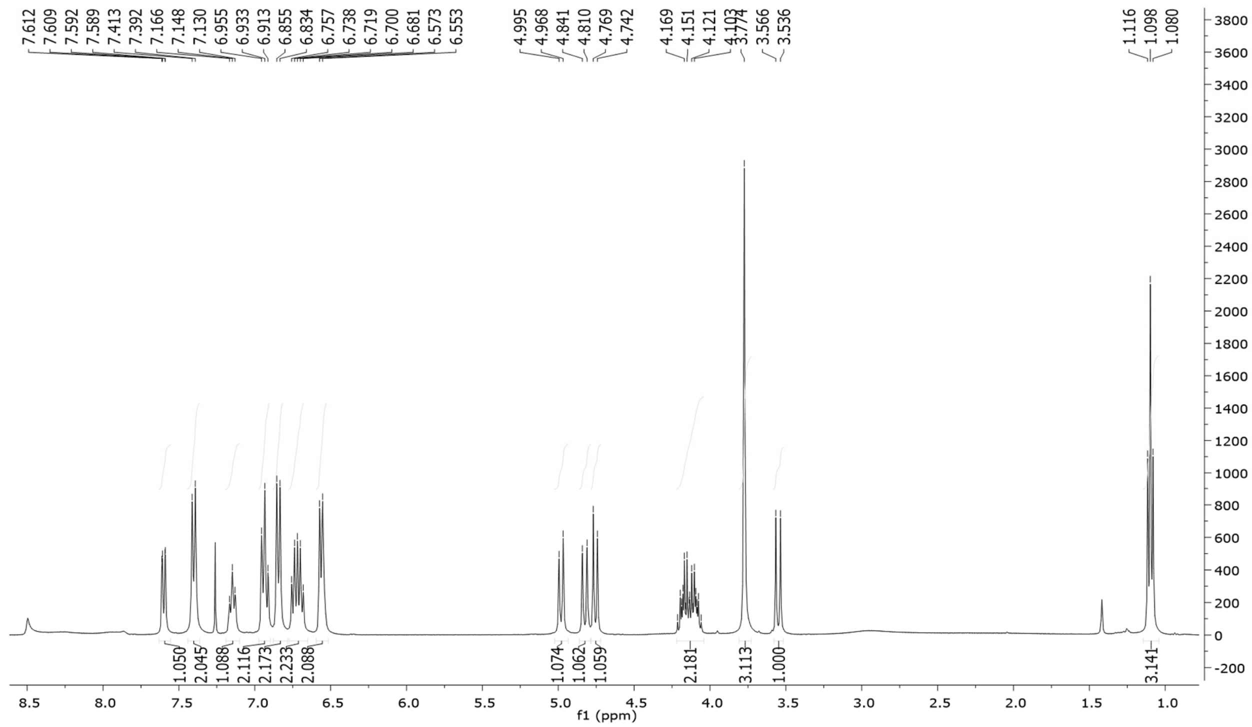

Figure S27. ^{1}H NMR spectrum of compound **9g** (400 MHz, $CDCl_3$)

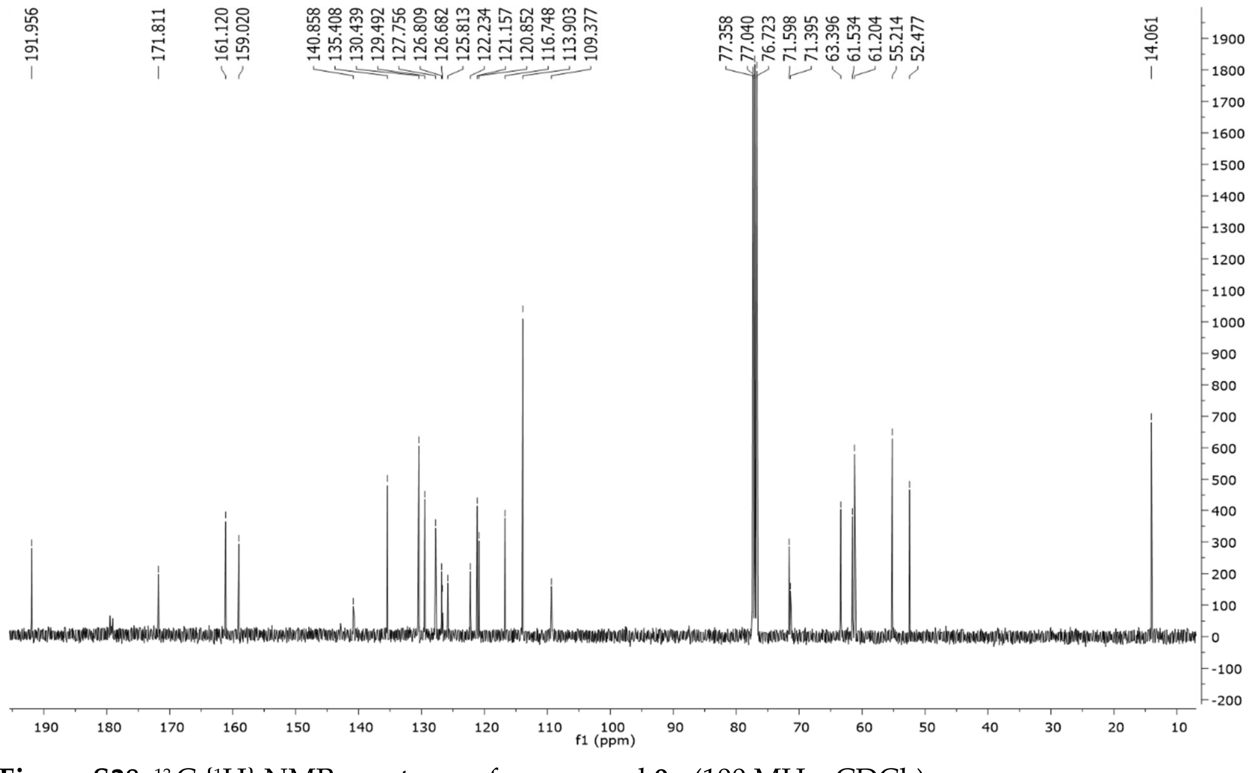

Figure S28. ^{13}C {^{1}H} NMR spectrum of compound **9g** (100 MHz, $CDCl_3$)

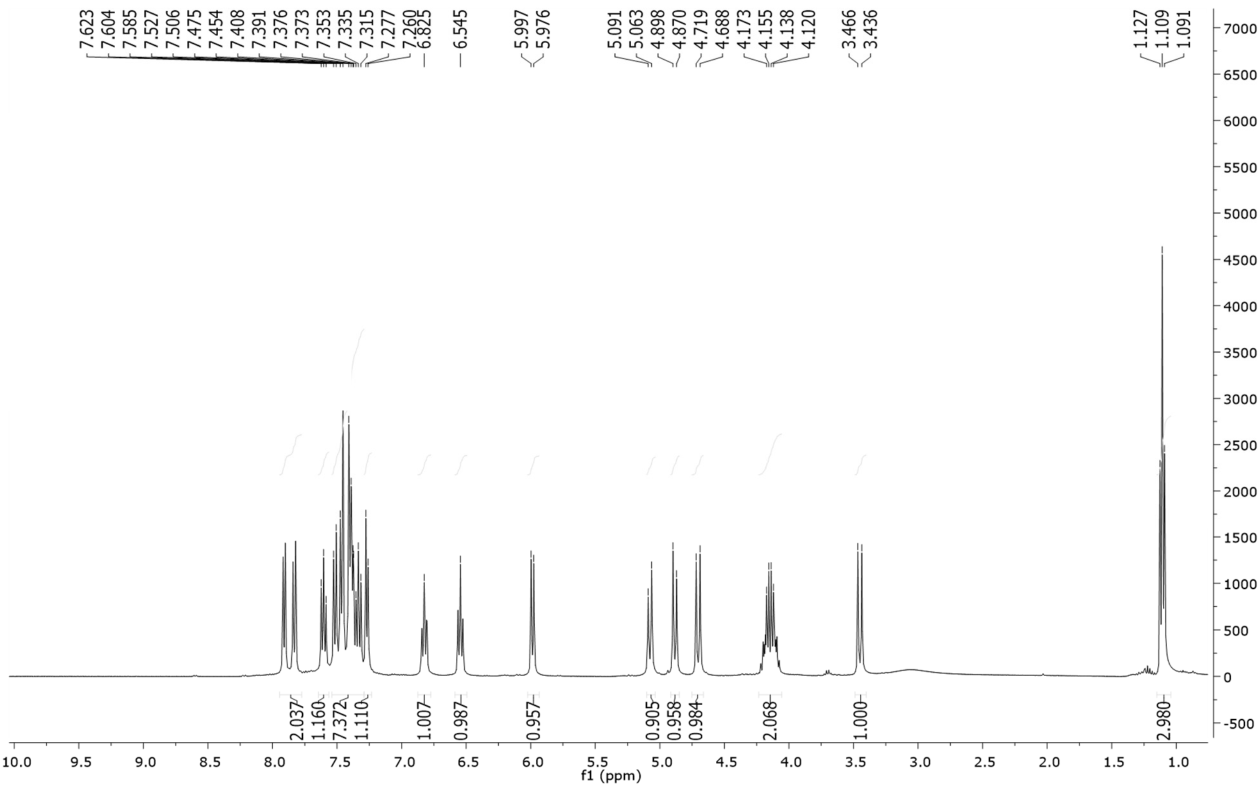

Figure S29. ^{1}H NMR spectrum of compound **9h** (400 MHz, $CDCl_3$)

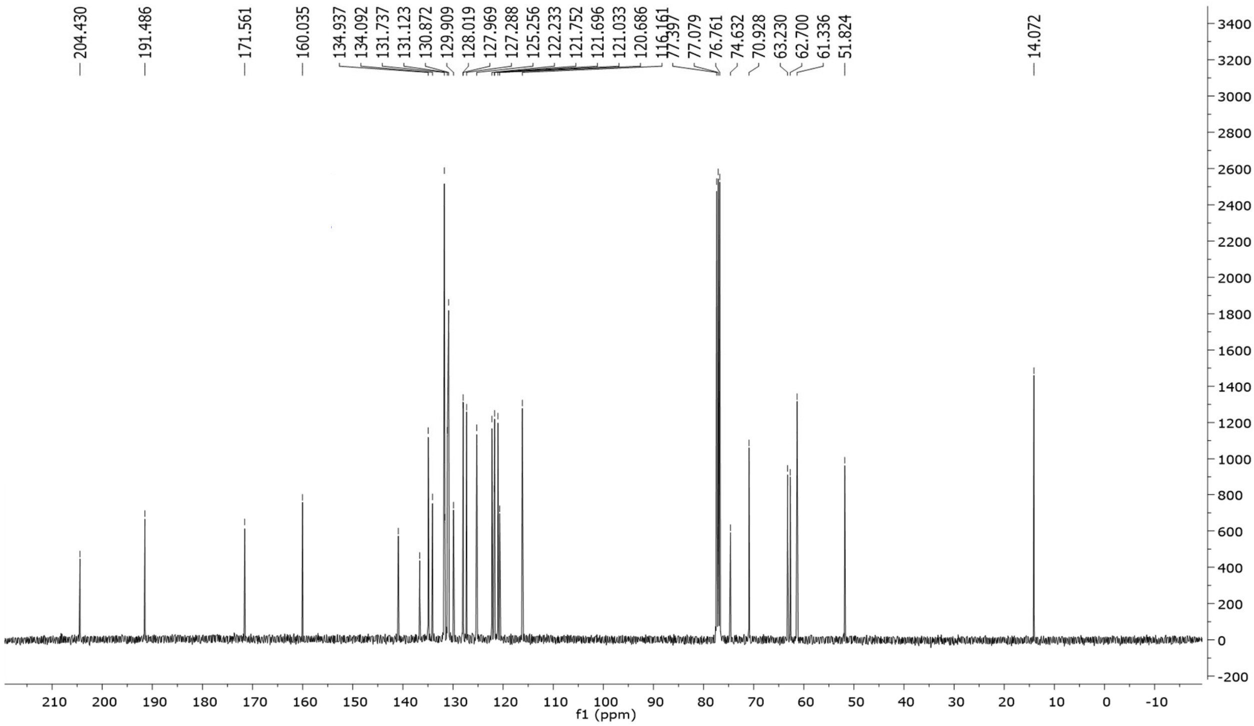

Figure S30. ^{13}C {^{1}H} NMR spectrum of compound **9h** (100 MHz, $CDCl_3$)

2. Table S1. The chemical shifts δ [ppm] from the experimental and theoretical 1H and 13C NMR spectra of compounds 4a and 9c.

Compound	**4a**		**9c**	
Atom no.	**Exp.**	**Calc.**	**Exp.**	**Calc.**
**C-2**	71.8	77.0	71.2	78.1
**C-3**	61.2	65.0	61.4	65.0
**C-4**	56.7	62.4	52.3	57.3
**C-5**	62.6	74.7	63.3	75.1
**O<u>C</u>H3/<u>C</u>H3**	-	-	55.2	54.7
**<u>C</u>H2 ester**	61.2	65.1	61.2	65.1
**<u>C</u>H3 ester**	13.9	14.7	14	14.6
**C-2″**	33.6	40.8	71.6	74.7
**C-4″**	194.1	204.0	191.8	202.4
**C-2'**	177.4	186.1	178.8	186.4
**(C=O)ester**	171.2	184.8	171.5	184.8
**H-4**	5.14	4.78	5.03	4.62
**H-5**	5.02	4.45	4.79	4.36
**NH oxindole**	8.18	6.53	7.89	6.55
**OC<u>H</u>3**	-	-	4.09	4.05
**C<u>H</u>2 ester**	4.12–4.2	4.36–4.69	4.11–4.2	4.36–4.67
**C<u>H</u>3 ester**	1.10	1.38<br>1.42<br>1.60	1.13	1.38<br>1.42<br>1.59
**H-2″**	3.42<br>2.73	2.94<br>3.21	3.56<br>4.84	3.96<br>4.22

3. Crystallographic data of 4a, 4e, and 9c.

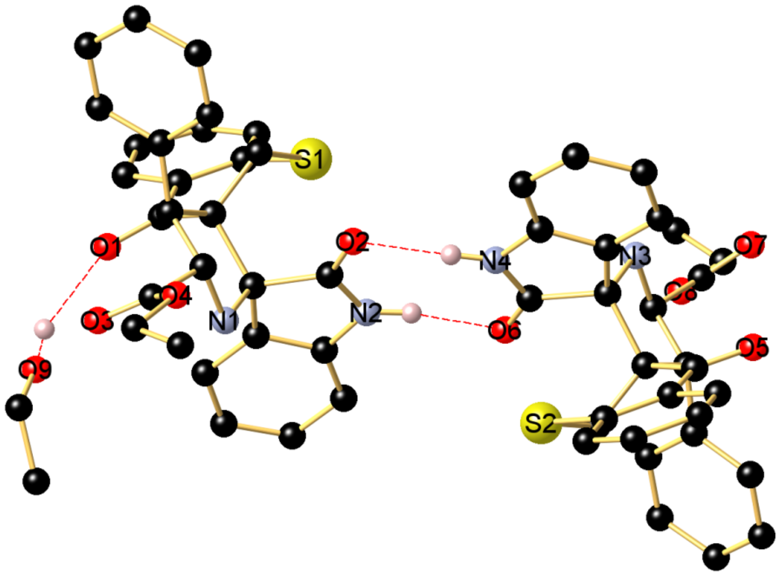

Figure S31. Supramolecular interactions occurring through hydrogen bonding in compound **4a**. Selected distances and angles: *d*(N2-H2······O6 1.92(3) Å, N2-H2-O6 171(2)2°); *d*(N4-H4······O 2 2.01(3) Å, N4-H4-O2 168(2)°); *d*(O9-H9A······O1 2.12 Å, O9-H9A-O1 155.9°).

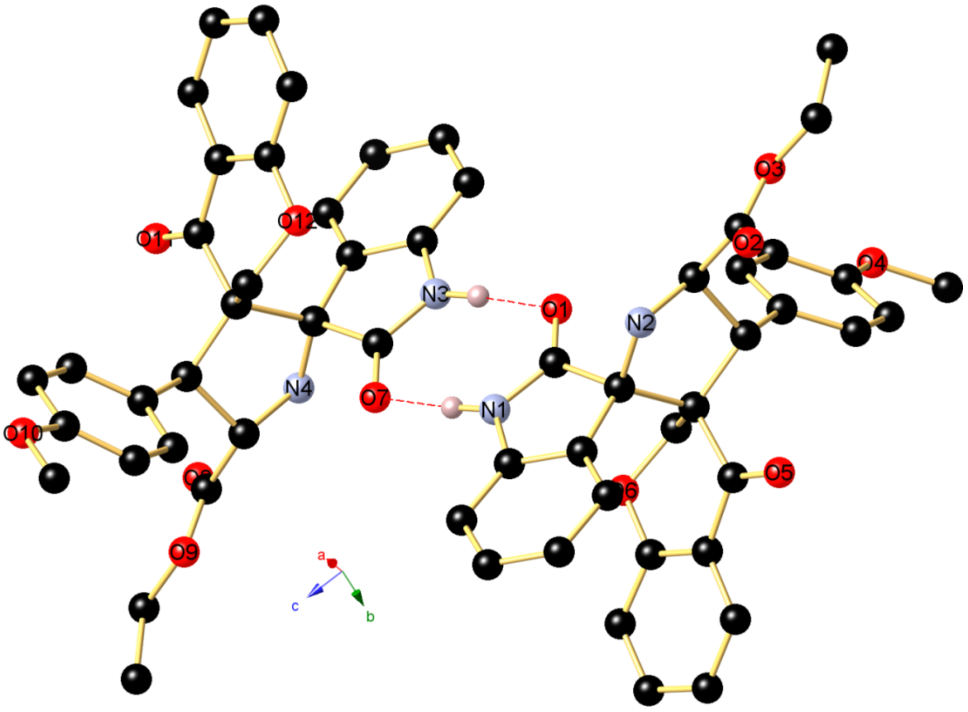

Figure S32. Supramolecular interactions occurring in compound **9c**. Selected distances and angles: *d*(N1-H1······O7 1.944(18) Å, N1-H1-O7 168.7(15°); *d*(N3-H3-O1 1.920(18) Å, N3-H3-O1 170.9(15)°).

Table S3. Crystal data collection and structure refinement of **4a, 4e, and 9e**

**Compound/formula**	**4a**/$C_{58}H_{54}N_4O_9S_4$	**4e**/$C_{33}H_{27}NO_4S$	**9c**/$C_{29}H_{26}N_2O_6$
**Formula weight**	1015.17	533.61	498.52
**Temperature/K**	100.0	100.0	100.0
**Wavelength/Å**	1.54178	0.71073	0.71073
**Crystal system**<br>**Space group**	monoclinic<br>$P2_1/n$	triclinic<br>P-1	monoclinic<br>$P2_1/n$
***a*/Å**	10.3905(5)	8.280(2)	12.3048
***b*/Å**	29.8564(16)	11.285(4)	18.3680(15)
***c*/Å**	15.9869(10)	14.788(3)	22.2980(17)
***α*/°**	90	95.993(11)	90
***β*/°**	97.798(2)	98.876(7)	94.285(2)
***γ*/°**	90	105.035(14)	90
**Volume/Å^3**	4913.6(5)	1303.3(6)	5025.6(6)
Z	4	2	8
**Density(calculated)g/cm^{-3}**	1.372	1.360	1.318
**Absorp. coefficient/mm^{-1}**	1.516	0.165	0.093
***F*(000)**	2136.0	560.0	2096.0
**Crystal size/mm^3**	0.66 × 0.454 × 0.118	0.433 × 0.184 × 0.074	0.312 × 0.234 × 0.08
**Theta range for data collection**	5.92 to 144.994	5.416 to 57.998	4.494 to 59.998
**Index ranges**	$-10 \leq h \leq 12$,<br>$-36 \leq k \leq 36$,<br>$-19 \leq l \leq 14$	$-11 \leq h \leq 11$,<br>$-15 \leq k \leq 15$,<br>$-18 \leq l \leq 20$	$-14 \leq h \leq 17$,<br>$-25 \leq k \leq 25$,

			-31 ≤ l ≤ 31
**Reflections collected**	43226	33330	111099
**Independent reflections**	9705 [R_{int} = 0.0465, R_{sigma} = 0.0331]	6942 [*R*int = 0.0313, *R*sigma = 0.0253]	14641 [R_{int} = 0.0507, R_{sigma} = 0.0372]
**Data/restraints/parameters**	9705/0/683	6942/0/358	14641/0/687
**Goodness-of-fit on F^2**	1.074	1.049	1.041
**Final *R* indices [*I* > 2sigma(*I*)]**	R_1 = 0.0475, wR_2 = 0.1244	R_1 = 0.0385, w*R*2 = 0.0960	R_1 = 0.0501, wR_2 = 0.1094
***R* indices (all data)**	R_1 = 0.0543, wR_2 = 0.1287	R_1 = 0.0457, w*R*2 = 0.1015	R_1 = 0.0710, wR_2 = 0.1185
**Largest diff. peak and hole/e Å^{-3}**	0.90/-0.46	0.45/-0.34	0.46/-0.30

3. DFT data

Table S2. The distribution patterns of the HOMOs and LUMOs of **4a-f** and **9a-h** calculated at the ωB97xd/6-31G(d, p) level of theory.

Compound	HOMO	LUMO
**4a**	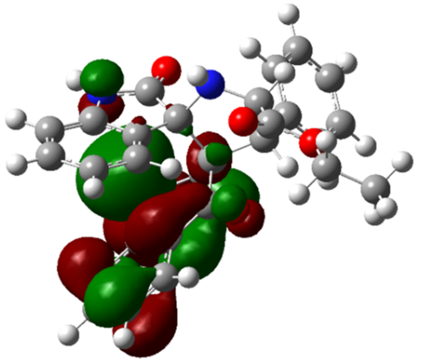	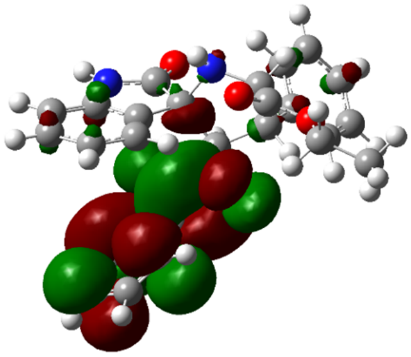
**4b**	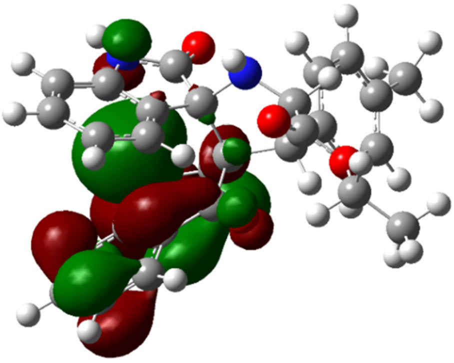	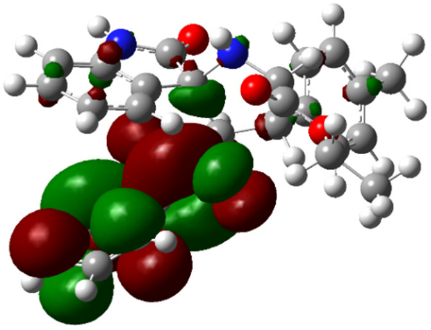
**4c**	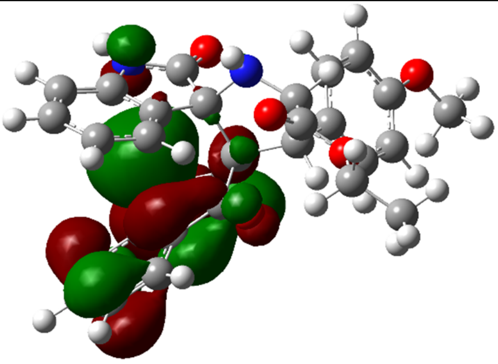	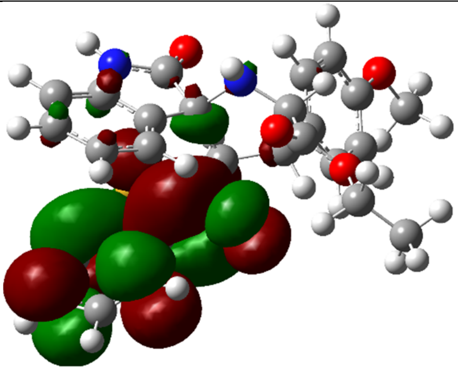
**4d**	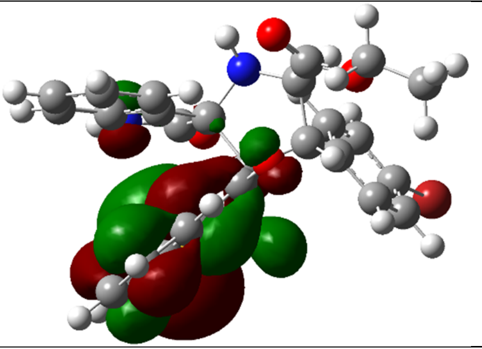	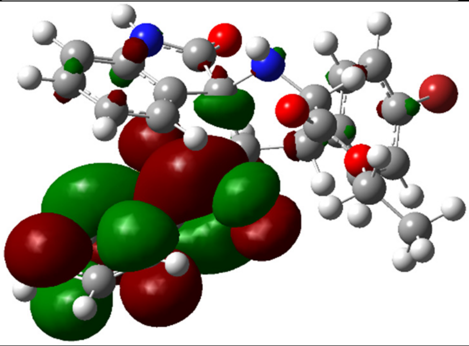

**4e**	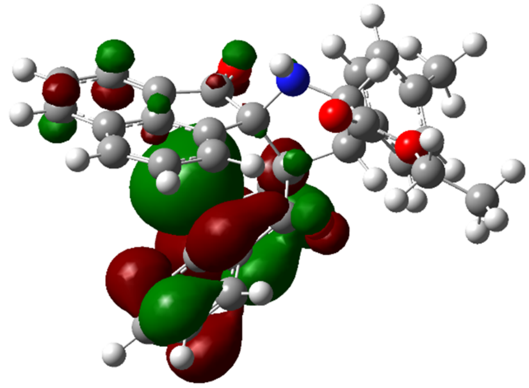	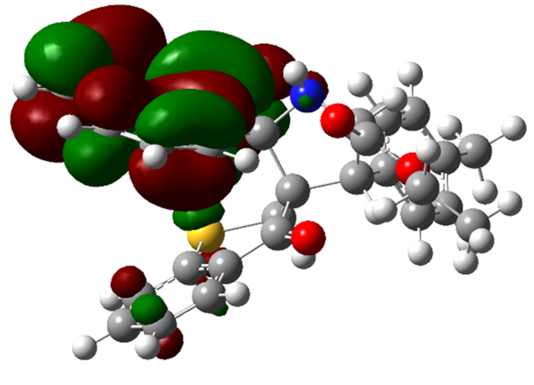
**4f**	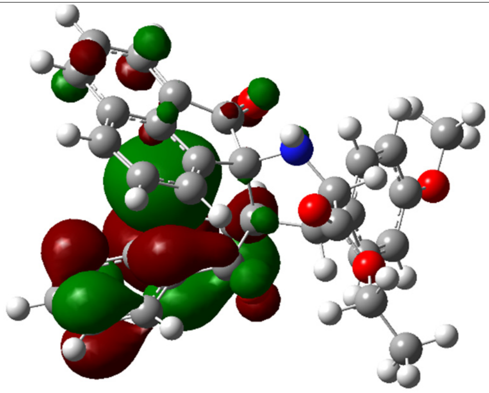	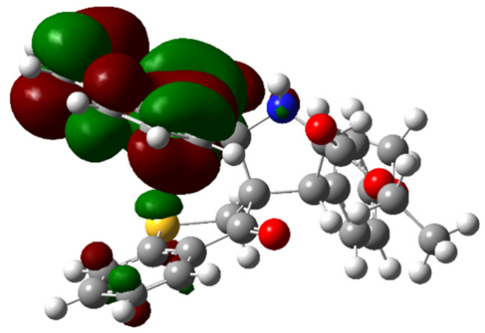
**9a**	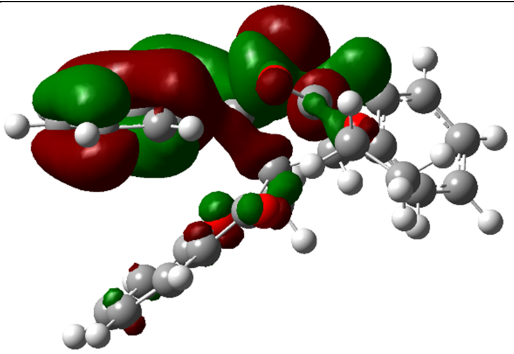	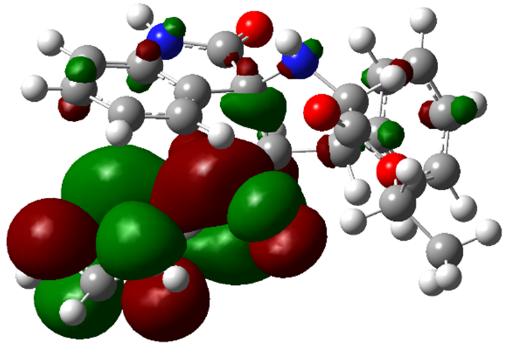
**9b**	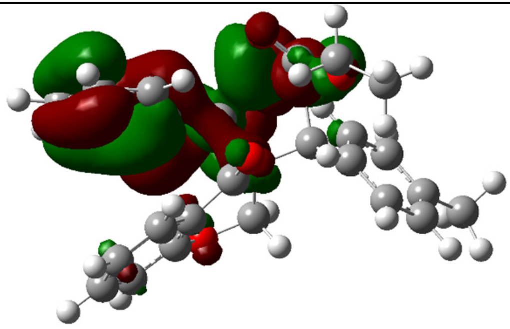	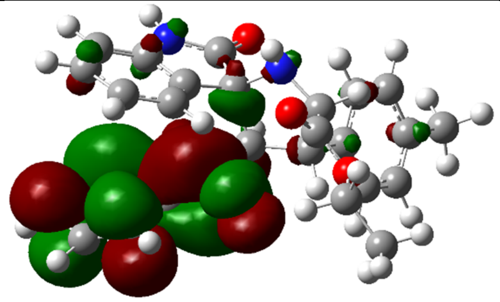

**9c**	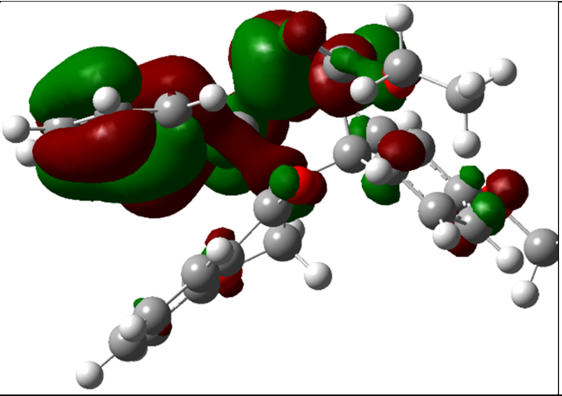	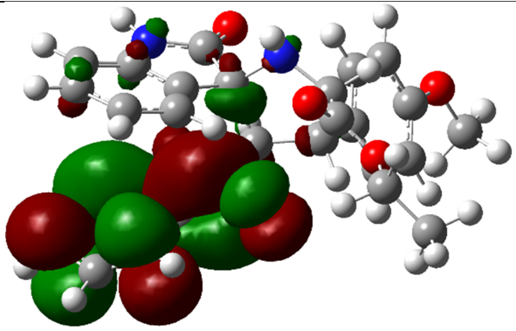
**9d**	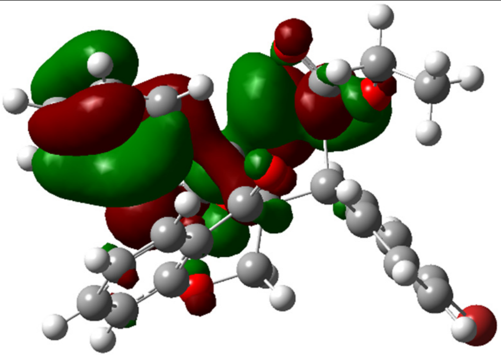	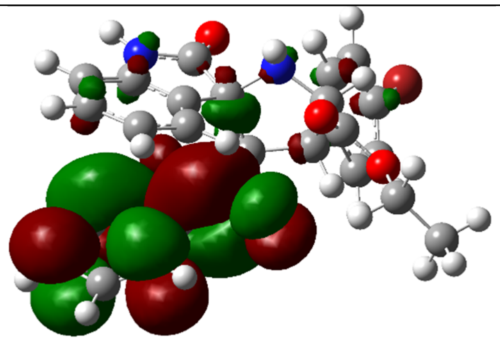
**9e**	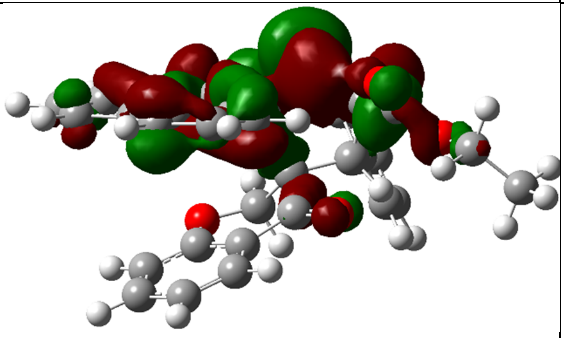	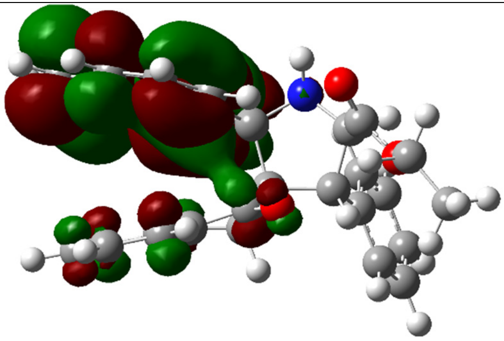
**9f**	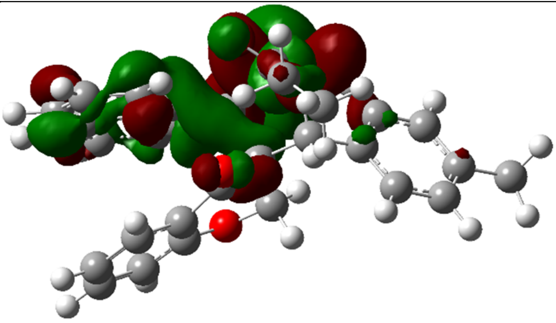	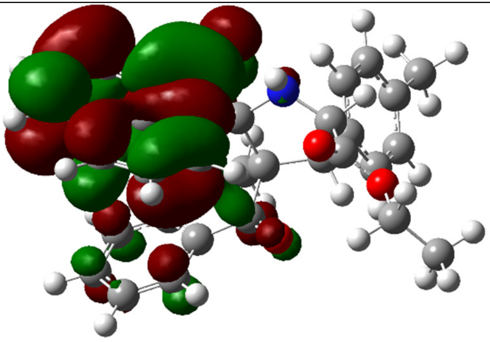

**9g**	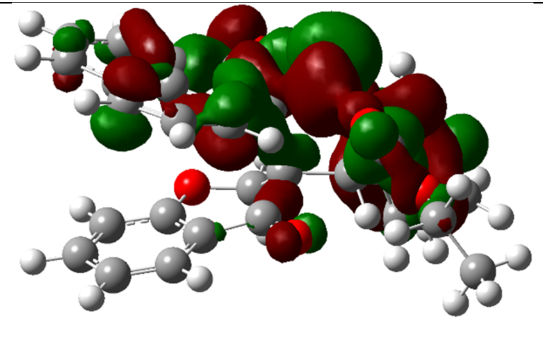	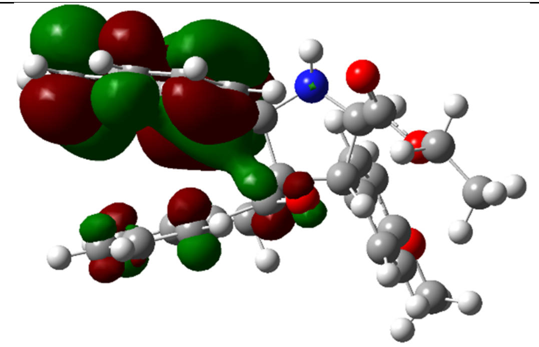
**9h**	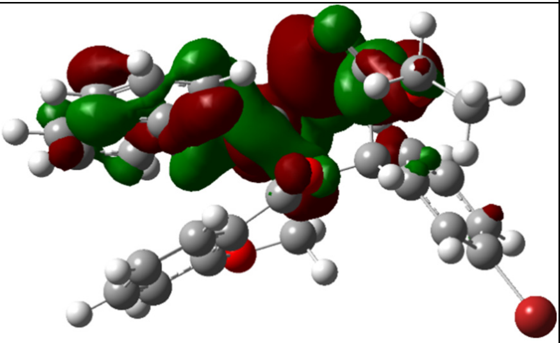	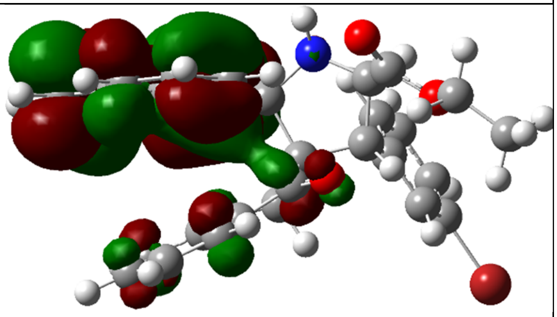

Cartesian coordinates and energies for 1d, *(Z,E)*-d3, *exo*-4d, *endo*-4d, *exo*-5d, *endo*-5d, TS-*exo*-4d, TS-*endo*-4d, TS-*exo*-5d and TS-*endo*-5d at the *ω*B97xd/6-31G(d, p) level of theory.

- **1d**

Coordinates (Angstroms)

Atom	X	Y	Z
S	-2.306551	-1.857296	0.117925
O	-2.720354	2.542565	-0.191633
C	-1.323544	-0.607740	1.005657
H	-1.781947	-0.468125	1.989892
H	-0.328286	-1.024612	1.153876
C	-1.301228	0.693259	0.263062
C	-2.628667	1.343056	0.030740
C	-3.849456	0.478116	0.021742
C	-3.813207	-0.929577	0.003639
C	-5.090578	1.124342	-0.061492
H	-5.097977	2.208470	-0.046841
C	-5.004906	-1.649512	-0.126099

C	-0.210263	1.335244	-0.184025
H	-0.378094	2.321636	-0.612138
H	-4.976476	-2.734321	-0.137446
C	-6.220675	-0.986494	-0.219610
C	-6.269313	0.407345	-0.176263
H	-7.220176	0.924949	-0.236671
H	-7.135862	-1.561773	-0.313028
C	1.187203	0.873347	-0.144982
C	1.551705	-0.430511	-0.501903
C	2.194696	1.777075	0.214818
C	2.881258	-0.835384	-0.467816
H	0.796146	-1.131157	-0.842572
C	3.525714	1.383133	0.263407
H	1.933622	2.799139	0.470722
C	3.854617	0.075119	-0.075360
H	3.153925	-1.845147	-0.751080
H	4.296784	2.086166	0.555777
Br	5.671596	-0.471541	-0.018781

Zero-point correction= 0.226781 (Hartree/Particle)

Electronic energy = -3661.014034

Internal energy = -3660.992567

Enthalpy= -3660.991442

Gibbs energy = -3661.072240

- *(Z,E)*-**d$_3$**

Coordinates (Angstroms)

Atom	X	Y	Z
O	0.361933	2.704205	-0.000344
O	-2.555148	-1.710998	0.000239
O	-3.841087	0.152725	0.000063
N	2.534144	1.857325	0.000354
N	-0.358854	-0.175330	0.000056
H	-0.446928	-1.196578	0.000139
C	-1.543074	0.418761	0.000015

H -1.608073 1.494792 -0.000102

C 0.874861 0.328897 0.000003

C 2.127763 -0.390019 -0.000059

C 3.134422 0.600137 -0.000038

C 1.154917 1.763095 -0.000110

C -2.673876 -0.491771 0.000122

C -5.023512 -0.670376 0.000172

H -5.005480 -1.312261 0.885201

C 3.835644 -2.073492 -0.000255

H 4.125887 -3.118655 -0.000340

C 2.482753 -1.736707 -0.000132

H 1.727344 -2.516577 -0.000131

C 4.480778 0.272162 -0.000115

C 4.819208 -1.082222 -0.000268

H 5.244488 1.042323 -0.000080

H 5.866501 -1.365728 -0.000369

H 3.023245 2.738214 -0.001348

H -5.005698 -1.312167 -0.884933

C -6.219438 0.254970 0.000363

H -6.219701 0.891330 -0.887984

H -7.137375 -0.337936 0.000427

H -6.219502 0.891215 0.888793

Zero-point correction= 0.230781 (Hartree/Particle)

Electronic energy = -799.228695

Internal energy = -799.208229

Enthalpy= -799.207105

Gibbs energy = -799.283843

- *exo*-**4d**

Coordinates (Angstroms)

Atom X Y Z

S -1.067009 -2.659632 1.169387

O -1.649575 1.735859 1.186125

O 0.699213 -2.083480 -1.922414

O	-1.554304	3.195498	-1.562220
O	0.069161	3.844713	-0.147174
N	-1.552458	-2.353600	-2.262367
N	-0.295724	0.759057	-2.032909
H	-0.977882	1.199787	-2.640914
C	0.134512	-1.320007	0.972501
H	0.495994	-1.026884	1.962437
H	0.966970	-1.758003	0.423194
C	-0.397974	-0.080009	0.245676
C	-1.539103	0.523914	1.093128
C	-2.528909	-0.360803	1.766871
C	-2.436564	-1.760971	1.816509
C	-3.654806	0.264535	2.325440
H	-3.709421	1.345844	2.275630
C	-4.675849	-0.471248	2.897794
H	-5.541365	0.029037	3.317298
C	-3.473099	-2.503433	2.393769
C	0.722310	1.010213	0.101946
H	0.626820	1.699021	0.940934
C	0.353230	1.747124	-1.195014
H	1.265738	2.119186	-1.677956
C	-0.916340	-0.309332	-1.243795
C	-2.423465	-0.406143	-1.447604
C	-2.729312	-1.638133	-2.030940
C	-0.446698	-1.684281	-1.829928
C	2.146644	0.490940	0.104215
C	2.877322	0.520134	1.293718
H	2.420006	0.913191	2.197324
C	4.188931	0.059298	1.348218
H	4.745139	0.088345	2.277881
C	4.774326	-0.429052	0.187804
C	4.075748	-0.462240	-1.013220
H	4.546389	-0.844433	-1.911696
C	2.764541	-0.001715	-1.047433

H	2.205458	-0.053418	-1.974726
C	-0.511333	2.987299	-0.978026
C	-0.673716	5.039231	0.164292
C	-4.753042	0.160006	-1.528867
H	-5.553975	0.867913	-1.345739
C	-3.436051	0.514638	-1.227077
H	-3.208818	1.496423	-0.828020
C	-4.583748	-1.864635	2.922673
H	-5.378422	-2.455988	3.365401
H	-3.398054	-3.585384	2.435093
C	-5.042467	-1.092310	-2.064932
C	-4.027479	-2.011613	-2.337228
H	-4.244713	-2.976637	-2.781522
H	-6.070260	-1.356832	-2.291094
H	-1.504213	-3.272716	-2.674201
C	0.124151	5.814454	1.187973
H	-0.823660	5.610429	-0.755773
H	-1.653397	4.743520	0.548501
H	1.104073	6.091020	0.791084
H	-0.412056	6.729435	1.451484
H	0.266756	5.223784	2.096329
Br	6.567308	-1.059046	0.241442

Zero-point correction = 0.463108 (Hartree/Particle)

Electronic energy = -4460.309188

Internal energy = -4460.267299

Enthalpy= -4460.266174

Gibbs energy = -4460.390856

- *endo*-**4d**

Coordinates (Angstroms)

Atom	X	Y	Z
S	-0.214300	1.745078	-1.688602
O	-1.284987	1.005585	2.222876
O	-1.813372	-1.953534	2.806988

O	0.681946	-3.027937	-1.837616
O	2.187781	-3.252704	-0.179033
N	-3.759110	-1.051662	1.967641
N	-1.318554	-2.620693	0.017715
H	-1.345148	-2.750531	-0.991380
C	-0.869300	0.087561	-1.307200
H	-1.850287	0.061341	-1.776227
H	-0.245773	-0.642908	-1.823527
C	-0.989568	-0.233365	0.192816
C	-1.410698	0.994351	1.010845
C	-2.036556	2.155618	0.318910
C	-1.549020	2.619658	-0.911320
C	-3.076165	2.837087	0.955607
H	-3.439118	2.464957	1.907878
C	-3.630155	3.969524	0.373648
H	-4.445597	4.489295	0.864189
C	-2.089542	3.769741	-1.482347
C	0.258153	-0.923772	0.804965
H	0.087416	-0.888004	1.880983
C	0.073282	-2.444252	0.427174
H	0.287953	-3.027963	1.324943
C	-2.049870	-1.423551	0.387430
C	-3.389583	-1.230002	-0.284216
C	-4.344221	-0.947480	0.699456
C	-2.475814	-1.520082	1.886028
C	1.604732	-0.268948	0.573720
C	2.201061	0.387203	1.653375
H	1.688601	0.413389	2.609960
C	3.439478	1.011116	1.537560
H	3.884623	1.510361	2.390170
C	4.097140	0.974772	0.316430
C	3.530876	0.339191	-0.782126
H	4.048444	0.316356	-1.733994
C	2.291424	-0.272518	-0.645814

H	1.877030	-0.762041	-1.520133
C	0.995814	-2.946948	-0.665161
C	3.233560	-3.570715	-1.123575
C	-5.112405	-1.036515	-1.945542
H	-5.426311	-1.090891	-2.981807
C	-3.781626	-1.304941	-1.611832
H	-3.079267	-1.583277	-2.391398
C	-3.126356	4.439257	-0.838386
H	-3.550366	5.328141	-1.293575
H	-1.708589	4.132213	-2.431170
C	-6.032871	-0.703948	-0.955957
C	-5.663290	-0.664168	0.390414
H	-6.386068	-0.439574	1.166553
H	-7.060856	-0.489270	-1.228284
H	-4.280926	-1.074534	2.831265
C	4.552630	-3.391976	-0.407136
H	3.082337	-4.597077	-1.467589
H	3.143918	-2.902898	-1.983634
H	4.620362	-4.051751	0.461115
H	5.371968	-3.632144	-1.089010
H	4.668158	-2.356505	-0.075712
Br	5.798639	1.799311	0.141663

Zero-point correction= 0.464111 (Hartree/Particle)

Electronic energy = -4460.296984

Internal energy = -4460.255220

Enthalpy= -4460.254096

Gibbs energy = -4460.379361

- *exo*-**5d**

Coordinates (Angstroms)

Atom	X	Y	Z
S	-2.078624	-1.679389	-1.707428
O	-1.731377	-0.553533	2.575046
O	-0.011589	1.588211	-2.049592

O	1.288800	3.477599	-0.044424
O	2.619317	2.326227	1.358827
N	-2.281270	1.806552	-2.241588
N	-0.977312	2.112272	0.906025
C	-0.612382	-1.194432	-0.763830
H	-0.153638	-2.092529	-0.340449
H	0.077753	-0.785830	-1.500292
C	-0.878447	-0.189954	0.360278
C	-1.838294	-0.831985	1.392682
C	-2.928630	-1.746727	0.946202
C	-3.147446	-2.136955	-0.385215
C	-3.831245	-2.175979	1.932229
H	-3.647520	-1.866744	2.954588
C	-4.934829	-2.945268	1.612998
H	-5.623420	-3.261103	2.388607
C	-4.268556	-2.910446	-0.706665
C	0.444892	0.200164	1.123396
H	0.343401	-0.249998	2.108857
C	0.341838	1.739324	1.364973
H	0.409253	1.912445	2.446629
H	-1.510626	1.193365	-0.084563
H	-3.026806	1.318985	-0.142540
H	-3.415376	1.672943	-1.434693
H	-1.129359	1.541437	-1.565804
C	1.761630	-0.315354	0.573094
C	2.484593	-1.227351	1.342728
H	2.076291	-1.580876	2.284893
C	3.739800	-1.680047	0.945209
H	4.296236	-2.374164	1.564149
C	4.272256	-1.211734	-0.246876
C	3.565984	-0.323773	-1.050763
H	3.991961	0.033027	-1.981447
C	2.316160	0.116348	-0.636403
H	1.767989	0.820839	-1.253280

C	1.453439	2.605547	0.779977
C	3.791636	2.958919	0.812597
C	-5.323826	1.437773	0.534640
H	-6.080217	1.358819	1.307792
C	-3.979464	1.240779	0.859317
H	-3.688442	1.024440	1.881189
C	-5.156829	-3.304253	0.281795
H	-6.020920	-3.903668	0.014882
H	-4.433602	-3.211069	-1.736440
C	-5.696075	1.741040	-0.772928
C	-4.740084	1.878099	-1.781724
H	-5.022415	2.145206	-2.793923
H	-6.743578	1.890885	-1.013252
H	-2.289792	2.059235	-3.218127
C	4.997901	2.220968	1.349656
H	3.739534	2.906078	-0.278200
H	3.789890	4.012519	1.105899
H	4.997588	1.181208	1.011765
H	5.909421	2.702040	0.985793
H	5.012093	2.238597	2.442329
Br	6.015977	-1.752560	-0.768192
H	-1.031316	3.082066	0.615110

Zero-point correction= 0.463138 (Hartree/Particle)

Electronic energy = -4460.303788

Internal energy = -4460.261894

Enthalpy= -4460.260770

Gibbs energy = -4460.385069

- *endo*-**5d**

Coordinates (Angstroms)

Atom	X	Y	Z
S	-0.749237	-1.735560	-2.100891
O	1.475936	-2.123031	1.668831
O	2.359750	0.735478	2.388968

O	0.418737	3.254479	1.375032
O	1.748977	3.995729	-0.289841
N	4.065583	-0.646107	1.723730
N	2.398582	1.225197	-0.630326
C	0.886948	-1.117120	-1.609989
H	1.600175	-1.924054	-1.784496
H	1.115912	-0.310109	-2.309332
C	1.047251	-0.631272	-0.159733
C	0.721386	-1.798328	0.767602
C	-0.579593	-2.494649	0.584625
C	-1.315739	-2.478599	-0.611057
C	-1.114439	-3.142747	1.708463
H	-0.528044	-3.143313	2.620638
C	-2.363614	-3.734627	1.666728
H	-2.770737	-4.216601	2.548358
C	-2.576704	-3.085613	-0.650813
C	0.244377	0.663332	0.230787
H	0.448330	0.792113	1.296721
C	1.042902	1.768547	-0.464817
H	0.648232	2.007100	-1.460058
C	2.510201	-0.072348	0.026068
C	3.670617	-0.888143	-0.513815
C	4.530070	-1.217266	0.539047
C	2.910633	0.054603	1.541829
C	-1.259425	0.722657	0.078856
C	-2.049290	0.304865	1.153549
H	-1.573088	-0.044508	2.064562
C	-3.436678	0.307923	1.083180
H	-4.031444	-0.035649	1.921268
C	-4.044502	0.763077	-0.079451
C	-3.291301	1.220998	-1.152495
H	-3.776961	1.584939	-2.050327
C	-1.903549	1.196481	-1.065146
H	-1.332009	1.537182	-1.921300

C	1.014452	3.069182	0.330480
C	1.854621	5.278349	0.362380
C	5.194365	-1.966280	-2.027611
H	5.472263	-2.245051	-3.038081
C	4.027721	-1.228408	-1.809743
H	3.424778	-0.912133	-2.653315
C	-3.096585	-3.698487	0.478049
H	-4.080007	-4.154416	0.430772
H	-3.149623	-3.072462	-1.572667
C	6.005492	-2.332953	-0.956602
C	5.691300	-1.949081	0.348123
H	6.340688	-2.197360	1.180250
H	6.908446	-2.907161	-1.136764
H	4.536475	-0.694876	2.613834
C	2.727754	6.156997	-0.504125
H	0.849556	5.691047	0.482524
H	2.283230	5.129675	1.357022
H	2.285912	6.289350	-1.494728
H	2.830217	7.139965	-0.037858
H	3.724424	5.723654	-0.618310
Cl	-5.941171	0.767925	-0.200408
H	3.133653	1.872875	-0.374106

Zero-point correction= 0.463203 (Hartree/Particle)

Electronic energy = -4460.301209

Internal energy = -4460.259197

Enthalpy= -4460.258073

Gibbs energy = -4460.384203

- TS-*exo*-**4d**

Coordinates (Angstroms)

Atom	X	Y	Z
S	2.613874	-2.227575	1.059995
O	2.668773	-0.085394	-2.827445
O	-0.352143	0.756748	2.956203

O	-2.454088	2.666181	-1.518943
O	-4.036507	1.928731	-0.075270
N	1.931123	1.031142	2.570766
N	-0.565888	2.036871	0.279125
C	1.581335	-2.560509	-0.401629
H	2.077869	-3.342308	-0.985247
H	0.631162	-2.956304	-0.045546
C	1.422641	-1.325468	-1.232115
C	2.683139	-0.763161	-1.809251
C	3.961186	-1.012854	-1.076713
C	4.029316	-1.589100	0.206140
C	5.142279	-0.558030	-1.676235
H	5.066777	-0.112808	-2.661828
C	6.361746	-0.647924	-1.027418
H	7.265314	-0.291286	-1.509349
C	5.259329	-1.660969	0.866226
C	0.274289	-0.689907	-1.509388
H	0.350618	0.146548	-2.201803
C	-1.834927	1.871061	0.613641
H	-2.089777	1.433699	1.564767
C	0.559603	1.742684	0.930216
C	1.916054	1.974093	0.493180
C	2.738106	1.527742	1.548921
C	0.590007	1.127329	2.253598
C	-1.071949	-0.991730	-0.998093
C	-2.166487	-0.912504	-1.867938
H	-2.006478	-0.660461	-2.911355
C	-3.457594	-1.156698	-1.417959
H	-4.297820	-1.107631	-2.100852
C	-3.656963	-1.457352	-0.075463
C	-2.594789	-1.516022	0.817355
H	-2.768040	-1.722229	1.866912
C	-1.306124	-1.282733	0.350952
H	-0.486074	-1.263310	1.061712

C	-2.782567	2.204509	-0.432470
C	-5.051306	2.168494	-1.066982
C	3.881249	2.602530	-0.723968
H	4.345890	3.012976	-1.613965
C	2.493177	2.510314	-0.654779
H	1.881498	2.847919	-1.485755
C	6.413452	-1.189429	0.256751
H	7.360609	-1.260356	0.781542
H	5.311052	-2.104468	1.855497
C	4.680847	2.163487	0.333178
C	4.119276	1.611978	1.484383
H	4.741213	1.251416	2.296093
H	5.760320	2.231532	0.252693
H	2.259907	0.674985	3.453965
C	-6.388678	1.922355	-0.406312
H	-4.950425	3.190593	-1.440533
H	-4.884583	1.481964	-1.902780
H	-6.570194	2.643003	0.394791
H	-7.186705	2.016626	-1.146809
H	-6.417839	0.912452	0.010134
Br	-5.419713	-1.774625	0.550495
H	-0.465160	2.451187	-0.652068

Zero-point correction= 0.458859 (Hartree/Particle)

Electronic energy = -4460.270780

Internal energy = -4460.227758

Enthalpy= -4460.226634

Gibbs energy = -4460.355380

- TS-*endo*-**4d**

Coordinates (Angstroms)

Atom	X	Y	Z
S	-3.726483	-2.701971	-0.327423
O	-2.718690	0.703718	-2.735385
O	-0.501433	3.237532	-1.037480

O	3.825718	1.192712	1.230763
O	4.326600	2.587606	-0.488762
N	-2.215519	2.307656	0.240893
N	1.217614	1.686644	0.826219
H	1.668451	1.095822	1.532237
C	-2.121444	-1.869506	-0.115100
H	-2.116468	-1.415121	0.880084
H	-1.382738	-2.673371	-0.125277
C	-1.810475	-0.853850	-1.182266
C	-2.919602	-0.058456	-1.798691
C	-4.282198	-0.137122	-1.192867
C	-4.736996	-1.254155	-0.476590
C	-5.125361	0.967853	-1.343989
H	-4.763751	1.815995	-1.915911
C	-6.393997	0.977570	-0.781500
H	-7.034292	1.845101	-0.895881
C	-6.017066	-1.249658	0.077447
C	-0.568177	-0.522453	-1.583246
H	-0.527048	0.218198	-2.379100
C	2.100239	2.249885	0.019638
H	1.767408	2.895085	-0.777533
C	-0.115439	1.752367	0.845626
C	-1.019785	1.065996	1.735148
C	-2.318407	1.430153	1.316172
C	-0.895676	2.530216	-0.110803
C	0.738848	-0.977189	-1.101422
C	1.838359	-0.827269	-1.958580
H	1.686353	-0.430509	-2.957451
C	3.121150	-1.179317	-1.559633
H	3.955733	-1.077172	-2.243293
C	3.318593	-1.650309	-0.267451
C	2.254197	-1.793358	0.614962
H	2.423018	-2.143215	1.626489
C	0.972469	-1.467699	0.192397

H	0.164820	-1.544105	0.909154
C	3.488311	1.942043	0.324225
C	5.736352	2.364362	-0.280805
C	-1.998901	-0.346804	3.409193
H	-1.888791	-1.046470	4.230463
C	-0.860580	0.176187	2.795472
H	0.127307	-0.115403	3.139553
C	-6.838208	-0.137003	-0.071360
H	-7.826935	-0.139453	0.375162
H	-6.363565	-2.112778	0.635981
C	-3.276252	0.016384	2.975167
C	-3.453528	0.915602	1.922262
H	-4.445241	1.185653	1.574513
H	-4.149084	-0.408714	3.459045
H	-2.990971	2.721509	-0.251228
C	6.238963	1.169285	-1.094415
H	6.201241	3.297221	-0.599046
H	5.909124	2.228545	0.787246
H	6.927506	1.482807	-1.881646
H	6.740692	0.436050	-0.461320
H	5.393559	0.668481	-1.570901
Br	5.066938	-2.076794	0.316303

Zero-point correction= 0.459248 (Hartree/Particle)

Electronic energy = -4460.267383

Internal energy = -4460.224147

Enthalpy= -4460.223023

Gibbs energy = -4460.352871

- TS-*exo*-**5d**

Coordinates (Angstroms)

Atom	X	Y	Z
S	-1.563994	-2.255020	-1.621656
O	-1.977362	-0.742732	2.512934
O	-0.194533	1.173831	-1.697761

O	0.785487	3.457045	-0.492043
O	2.515296	2.314700	0.415253
N	-2.506721	1.322418	-1.922726
N	-0.856806	1.682687	1.200198
C	-0.478025	-2.690145	-0.233791
H	-0.803456	-3.667366	0.139011
H	0.528455	-2.804462	-0.634596
C	-0.528358	-1.702696	0.894374
C	-1.869943	-1.354960	1.449773
C	-3.086704	-1.688416	0.657460
C	-3.061811	-2.066052	-0.699647
C	-4.326033	-1.526076	1.291277
H	-4.325236	-1.210359	2.327883
C	-5.513845	-1.733279	0.613334
H	-6.462273	-1.596685	1.120397
C	-4.265014	-2.256682	-1.384493
C	0.549702	-1.166198	1.492758
H	0.371664	-0.649367	2.431911
C	0.440364	1.894103	1.278218
H	0.920908	1.628960	2.208733
C	-1.694869	1.704134	0.146211
C	-3.131222	1.697937	0.235237
C	-3.601316	1.462332	-1.073876
C	-1.307299	1.396641	-1.223820
C	1.934735	-1.179269	0.991558
C	3.001824	-1.435442	1.859031
H	2.806348	-1.682452	2.897764
C	4.314457	-1.388653	1.405674
H	5.137961	-1.593181	2.079682
C	4.555534	-1.062777	0.075452
C	3.515874	-0.787487	-0.804334
H	3.723645	-0.508724	-1.830756
C	2.207733	-0.843096	-0.340472
H	1.391940	-0.556477	-0.997595

C	1.220967	2.628439	0.282493
C	3.441514	2.973900	-0.464947
C	-5.399617	1.879034	0.985656
H	-6.118016	2.041249	1.782340
C	-4.035546	1.918303	1.270520
H	-3.690213	2.123404	2.279327
C	-5.479094	-2.091725	-0.733587
H	-6.403102	-2.251193	-1.279728
H	-4.244450	-2.548621	-2.429574
C	-5.850273	1.641000	-0.313856
C	-4.955186	1.434383	-1.365255
H	-5.307533	1.254620	-2.375234
H	-6.916307	1.615940	-0.515220
H	-2.548385	0.934565	-2.851879
C	4.825545	2.829640	0.131632
H	3.376790	2.491483	-1.445116
H	3.143939	4.018455	-0.577719
H	4.964524	3.505590	0.979735
H	4.977840	1.803167	0.472133
H	5.585153	3.053914	-0.621515
Br	6.347641	-0.920939	-0.533094
H	-1.284682	1.330868	2.059967

Zero-point correction= 0.458765 (Hartree/Particle)

Electronic energy = -4460.250304

Internal energy = -4460.207166

Enthalpy= -4460.206042

Gibbs energy = -4460.334171

- TS-*endo*-**5d**

Coordinates (Angstroms)

Atom	X	Y	Z
S	5.250379	-1.233058	-1.714644
O	4.551015	0.932232	2.032186
O	-4.608737	-0.245295	0.213170

O	-6.445577	0.901366	-2.401918
O	-5.497306	2.140781	-0.771261
N	-2.802347	0.220662	1.600371
N	-2.985434	1.344620	-1.756597
C	3.638027	-0.540734	-1.223546
H	3.469305	0.372653	-1.802848
H	2.904446	-1.283498	-1.537159
C	3.557793	-0.279531	0.252784
C	4.665734	0.490359	0.894756
C	5.929494	0.734441	0.134224
C	6.296079	0.013053	-1.014953
C	6.815116	1.692178	0.644416
H	6.515489	2.239124	1.531396
C	8.042390	1.925990	0.045356
H	8.714712	2.673620	0.451416
C	7.539029	0.241867	-1.610872
C	2.548809	-0.663149	1.056083
H	2.687615	-0.446606	2.113021
C	-4.119044	1.265604	-2.434640
H	-4.039367	1.180582	-3.509001
C	-2.732157	1.163688	-0.446592
C	-1.502592	1.491598	0.229335
C	-1.589349	0.893075	1.504831
C	-3.541464	0.325565	0.432552
C	1.278408	-1.319358	0.730128
C	0.691145	-2.141660	1.702536
C	1.206955	-2.308136	2.643144
C	-0.541550	-2.740132	1.491445
H	-0.985221	-3.373449	2.250615
C	-1.208603	-2.499977	0.294428
C	-0.663988	-1.677512	-0.681937
H	-1.209208	-1.475936	-1.596388
C	0.573761	-1.086199	-0.456767
H	0.954293	-0.387267	-1.191886

C	-5.462956	1.373360	-1.860197
C	-6.742458	2.189645	-0.056664
C	0.657775	2.348725	0.818694
H	1.550191	2.909835	0.562385
C	-0.374364	2.233909	-0.111452
H	-0.294892	2.718924	-1.079886
C	8.406240	1.188886	-1.081550
H	9.365210	1.360340	-1.559335
H	7.818692	-0.316166	-2.498539
C	0.553483	1.756448	2.078665
C	-0.577795	1.023947	2.441940
H	-0.656506	0.557785	3.417985
H	1.369591	1.860295	2.786120
H	-3.067731	-0.397398	2.350151
C	-6.462828	2.828688	1.285175
H	-7.465117	2.766854	-0.641055
H	-7.121337	1.171044	0.052458
H	-6.064740	3.839226	1.162678
H	-7.388352	2.890279	1.863227
H	-5.740251	2.231719	1.847178
Br	-2.911369	-3.278404	0.008976
H	-2.157436	1.502971	-2.328441

Zero-point correction= 0.458306 (Hartree/Particle)

Electronic energy = -4460.245343

Internal energy = -4460.201869

Enthalpy= -4460.200745

Gibbs energy = -4460.334528
